# Evaluating the Efficacy of a Social Media–Based Intervention (Warna-Warni Waktu) to Improve Body Image Among Young Indonesian Women: Parallel Randomized Controlled Trial

**DOI:** 10.2196/42499

**Published:** 2023-04-03

**Authors:** Kirsty M Garbett, Sharon Haywood, Nadia Craddock, Caterina Gentili, Kholisah Nasution, L Ayu Saraswati, Bernie Endyarni Medise, Paul White, Phillippa C Diedrichs, Heidi Williamson

**Affiliations:** 1 Centre for Appearance Research University of the West of England Bristol United Kingdom; 2 Faculty of Medicine Universitas Indonesia Jakarta Indonesia; 3 Department of Women, Gender, and Sexuality Studies University of Hawai’i at Mānoa Honolulu, HI United States; 4 Faculty of Environment and Technology University of the West of England Bristol United Kingdom

**Keywords:** body image, body dissatisfaction, Indonesia, adolescent mental health, randomized controlled trial, eHealth intervention, Southeast Asia, social media, lower- and middle-income countries, LMICs

## Abstract

**Background:**

Body dissatisfaction is a global issue, particularly among adolescent girls and young women. Effective body image interventions exist but face barriers to scaling up, particularly in lower- and middle-income countries, such as Indonesia, where a need exists.

**Objective:**

We aimed to evaluate the acceptability and efficacy of *Warna-Warni Waktu*, a social media–based, fictional 6-episode video series with self-guided web-based activities for improving body image among young Indonesian adolescent girls and young women. We hypothesized that *Warna-Warni Waktu* would increase trait body satisfaction and mood and decrease internalization of appearance ideals and skin shade dissatisfaction relative to the waitlist control condition. We also anticipated improvements in state body satisfaction and mood immediately following each video.

**Methods:**

We conducted a web-based, 2-arm randomized controlled trial among 2000 adolescent girls and young women, aged 15 to 19 years, recruited via telephone by an Indonesian research agency. Block randomization (1:1 allocation) was performed. Participants and researchers were not concealed from the randomized arm. Participants completed self-report assessments of trait body satisfaction (primary outcome) and the internalization of appearance ideals, mood, and skin shade dissatisfaction at baseline (before randomization), time 2 (1 day after the intervention [T2]), and time 3 (1 month after the intervention [T3]). Participants also completed state body satisfaction and mood measures immediately before and after each video. Data were evaluated using linear mixed models with an intent-to-treat analysis. Intervention adherence was tracked. Acceptability data were collected.

**Results:**

There were 1847 participants. Relative to the control condition (n=923), the intervention group (n=924) showed reduced internalization of appearance ideals at T2 (*F*_1,1758_=40.56, *P*<.001, partial η^2^=0.022) and T3 (*F*_1,1782_=54.03, *P*<.001, partial η^2^=0.03) and reduced skin shade dissatisfaction at T2 (*F*_1,1744_=8.05, *P*=.005, partial η^2^=0.005). Trait body satisfaction improvements occurred in the intervention group at T3 (*F*_1, 1781_=9.02, *P*=.005, partial η^2^=0.005), which was completely mediated by the internalization change scores between baseline and T2 (indirect effect: β=.03, 95% CI 0.017-0.041; direct effect: β=.03, *P*=.13), consistent with the Tripartite Influence Model of body dissatisfaction. Trait mood showed no significant effects. Dependent sample *t* tests (2-tailed) found each video improved state body satisfaction and mood. Cumulative analyses found significant and progressive improvements in pre- and poststate body satisfaction and mood. Intervention adherence was good; participants watched an average of 5.2 (SD 1.66) videos. Acceptability scores were high for understandability, enjoyment, age appropriateness, usefulness, and likelihood to recommend.

**Conclusions:**

*Warna-Warni Waktu* is an effective eHealth intervention to reduce body dissatisfaction among Indonesian adolescent girls and young women. Although the effects were small, *Warna-Warni Waktu* is a scalable, cost-effective alternative to more intense interventions. Initially, dissemination through paid social media advertising will reach thousands of young Indonesian women.

**Trial Registration:**

ClinicalTrials.gov NCT05383807, https://clinicaltrials.gov/ct2/show/NCT05383807 ; ISRCTN Registry ISRCTN35483207, https://www.isrctn.com/ISRCTN35483207

**International Registered Report Identifier (IRRID):**

RR2-10.2196/33596

## Introduction

See Multimedia Appendix 1 for a full Bahasa Indonesia translation of this paper.

### Background

Globally, many young people, particularly adolescent girls and young women [1,2], experience negative thoughts and feelings about their body. This is often termed body dissatisfaction [3]. Prospectively, studies have found that body dissatisfaction among young women predicts negative mental and physical health consequences, such as disordered eating behaviors, low mood, and low self-esteem [4]. Therefore, action to reduce body dissatisfaction is warranted.

Interventions have been developed and rigorously evaluated to reduce body dissatisfaction among adolescent girls and young women, with promising findings [5-9]. These interventions typically rely on face-to-face provision, often in small-group settings, led by professionals or facilitators who have been professionally trained. This approach reduces the scalability of interventions because of the costly nature of this format, coupled with a global shortage of mental health professionals to deliver such interventions to those in need [10]. Furthermore, other barriers related to attending face-to-face care include the lack of anonymity and privacy in addressing sensitive issues [11] and logistical challenges, such as geographical distance and limited availability [12]. These barriers are amplified in lower- and middle-income countries (LMICs), where health care provision is even sparser; stigma attached to mental health is heightened; and in geographically diverse contexts such as Indonesia, such care is inaccessible to many [10,13]. Relatedly, most effective interventions to reduce body dissatisfaction have been developed and evaluated in high-income English-speaking regions, mainly Australia, the United States, and Western Europe [14]. Few have been specifically designed or culturally adapted for non-Western countries [15-17]. Research indicates that the nature of appearance concerns differs across world regions [18-20]. Therefore, it is imperative that cultural norms and standards are upheld and addressed in intervention design to ensure effectiveness and acceptability [21,22]. The design of culturally specific interventions is critical for combating the global burden of body dissatisfaction among girls and young women.

### Harnessing Social Media for Body Image Interventions

A viable solution to increase the scalability of mental health interventions, including those to reduce body dissatisfaction, is the use of social media platforms [23,24]. Despite the widely evidenced risks of social media for young people [25-27], one of the main advantages of these platforms is their ability to access information [28]. Social media use has surged in recent years, with global use among young people being almost ubiquitous [29]. Social media–based interventions circumvent several barriers to accessing care in more traditional face-to-face methods by reducing costs and overreliance on mental health care professionals, overcoming the need for physical proximity, and lessening the social stigma of participation given the relative anonymity afforded on social network sites [30]. Furthermore, social media–based interventions allow for targeting those in need within easily accessed digital spaces typically visited daily, a crucially important consideration given the high attrition rates often seen across other eHealth interventions, such as those delivered via apps [31]. With regard to body dissatisfaction, extensive research has documented the detrimental impact of viewing idealized images on social media [32]. However, little research has examined the potential of using social media as a tool to disseminate interventions to reduce body dissatisfaction, despite promising findings from the broader field of mental health [33].

### The Indonesian Context

Indonesia, a Southeast Asian nation, is the world’s largest archipelago and fourth most populous country [34]. Body dissatisfaction has been shown to affect at least half of young Indonesian women [35] (KM Garbett et al, unpublished data, March 2021), which is associated with disordered eating behaviors in this demographic [36]. Skin shade dissatisfaction and a desire to have lighter skin shade is also common [37,38], putting young Indonesian women at risk of using unsafe skin-lightening products [39] and experiencing low self-esteem [40]. Girls and young women in Indonesia express a desire to learn ways to feel better about their appearance [41], yet to the best of the authors’ knowledge, no eHealth intervention has been rigorously developed and evaluated for this population. Internet penetration across Indonesia is high [42], with many older girls and young women owning their own device to access their social media accounts [43]. This, along with the aforementioned benefits of using the internet for intervention delivery (ie, low cost, accessibility, reduced social stigma, and overcoming physical barriers to engagement), suggests that interventions delivered via social media are a particularly fruitful option for reducing body dissatisfaction among young Indonesian women.

### The Intervention

*Warna-Warni Waktu* (English translation: The Colors of Time) is a stand-alone, social media–based intervention designed to reduce body dissatisfaction among young Indonesian women aged between 15 and 19 years. The creation of the intervention was a collaborative effort among the academic authors of this paper with Girl Effect, an international nonprofit organization that creates empowering media content for girls; the Dove Self-Esteem Project, the social mission for Unilever’s personal care brand Dove; Percolate Galactic, an Indonesian creative agency focused on youth marketing; and young Indonesian women. The intervention comprised 6 short videos telling a fictitious story of a young woman named Putri as she navigates societal appearance pressures throughout adolescence and early adulthood. Each video is supplemented with several short interactive activities formatted for dissemination on social media platforms. The intervention targets the sociocultural risk factors for body dissatisfaction proposed by the Tripartite Influence Model of body dissatisfaction (ie, media, friends, and family), which can increase body dissatisfaction through the internalization of appearance ideals and social comparisons [44]. Furthermore, the intervention incorporates change techniques, including psychoeducation, media literacy, and cognitive dissonance, each of which has shown effectiveness in previous body image interventions [5,6,14,45]. The development of the intervention is described in detail in a study protocol paper [46] and is summarized in this paper.

### This Study

In this study, we evaluated the impact of *Warna-Warni Waktu* on improving body satisfaction and related outcomes among adolescent girls and young Indonesian women. We conducted a parallel randomized (1:1) efficacy trial to compare participation in *Warna-Warni Waktu* to a waitlist control condition, which was selected given that the primary interest of the study was the absolute impact of the intervention, and there is no alternative support for body dissatisfaction in Indonesia [47]. Our hypotheses were as follows: (1) participants randomized to the intervention condition would experience increased trait body satisfaction and mood and decreased internalization of appearance ideals and skin shade dissatisfaction at 1 day after the intervention, and at 1 month follow-up, relative to the waitlist control condition; (2) each video would elicit immediate state-based improvements in body satisfaction and mood; and (3) greater engagement and adherence to the intervention would result in greater improvements in trait and state body satisfaction and mood and greater reductions in the internalization of appearance ideals and skin shade dissatisfaction.

## Methods

### Trial Design

A 2-arm, web-based parallel randomized controlled trial was conducted to evaluate the efficacy of *Warna-Warni Waktu* using an intervention group and a waitlist control group (NCT05023213 and ISRCTN35483207). Block randomization was performed with a 1:1 allocation with blocks of 4, 6, and 8.

### Ethics Approval

Ethics approval was obtained from the Faculty of Medicine at Universitas Indonesia (588/UN2.F1/ETIK/PPM.00.002/2021) and the University of the West of England, Bristol (United Kingdom; HAS.21.04.138). The trial protocol was registered (International Registered Report Identifier PRR1-10.2196/33596) [46].

### Participants

A Jakarta-based research agency recruited and enrolled a sample of adolescent girls and young Indonesian women from 10 cities across the western, central, and eastern regions of Indonesia (Balikpapan, Bandung, Jakarta, Makassar, Manado, Medan, Palembang, Pontianak, Semarang, and Surabaya) offline, evenly split across age and socioeconomic status. Adolescent girls and young women were invited to participate if they met the following inclusion criteria: (1) were between the ages of 15 and 19 years, (2) had their own mobile phone, and (3) visited Facebook or Instagram daily. They were excluded from the study if they (1) followed the Girl Effect brand (Springster) on social media, (2) had previously accessed the Springster website, or (3) did not provide written consent from a parent or guardian (if they were aged <18 years).

The research agency recruited participants via phone using an existing database of previous adult participants aged >40 years. Individuals who had a daughter aged between 15 and 17 years were read the parental information sheet. If they had >1 eligible daughter, only one who met the needs of this trial’s age quota was selected; if >1 met the age quota needs, the daughter whose date of birth was closest to the date of the call was selected. Parents who provided verbal consent then supplied information regarding their socioeconomic status. Daughters were then screened by the recruiter to determine if they met the social media use inclusion criteria and whether they had previously accessed the Springster brand on social media and its website (exclusion criteria). If she provided verbal assent to participate, parents once again provided consent for their daughter’s participation, this time written via WhatsApp. A similar process was executed for eligible daughters aged 18 or 19 years, except that they themselves provided verbal and written consent. The identities of the parents and eligible daughters were confirmed on video calls through official photo-based IDs.

### The Intervention

As the development process and content of *Warna-Warni Waktu* have been described in detail in our protocol paper [46], we only provide a brief summary in this paper. This stand-alone, self-guided (ie, no human involvement or assistance is required) intervention (version 1), frozen during the trial, consists of 6 sequential short videos, each approximately 5 minutes in length. The narrative running through the videos centers on a young Indonesian woman experiencing common body image concerns, who acquires strategies to combat appearance-related pressures (eg, skin-lightening products disseminated on social media, appearance-based comparisons, and comments by friends) with the assistance of animated time travelers. The videos target key risk factors known to contribute to the development of body dissatisfaction, such as social media and influencers (video 2), appearance-based comparisons (video 3), appearance-based teasing (video 4), and body talk (video 5). Videos 1 and 6 do not address specific risk factors but introduce and conclude the overarching story of *Warna-Warni Waktu*. In addition, each video is supplemented with web-based activities designed to reinforce the video’s key message. Most of the activities are short, such as completing a comic strip or identifying changes to an edited photo. However, in videos 2 to 5, one activity for each video asks participants to write up to 250 words in response to a question about the video’s main message, referred to as “Your Own Words.” To incentivize engagement with these longer activities, participants with the best response from each of the “Your Own Words” activities were awarded Rp 50,000 (approximately US $3.50) in mobile phone credit, an effective tool Girl Effect has used with this demographic. Responses were assessed by the fifth author (KN). This incentive will also be used when the intervention is live.

*Warna-Warni Waktu* was informed by the Tripartite Influence Model [44] in that the videos target 3 sociocultural influences (ie, friends, family, and the media), which influence the psychological processes of the internalization of appearance ideals and appearance-based social comparisons. The videos also address these psychological processes directly through the delivery of media literacy education and by elucidating how appearance-based comparisons can lead to body dissatisfaction. Furthermore, supplementary activities use body image change techniques known to be effective in the reduction of body dissatisfaction, namely, cognitive dissonance and psychoeducation [5].

The videos and activities were designed to be disseminated on Facebook and Instagram through sequential marketing, targeting adolescent girls and young Indonesian women. The videos will also be hosted on YouTube for free. However, for the trial, the intervention was accurately recreated on the survey platform Qualtrics (Qualtrics International Inc) to ensure that adherence data at the individual level were obtained. Each of the 6 videos and corresponding activities were embedded into 6 different Qualtrics questionnaires; participants were expected to engage with 1 video and its associated activities per day (within 24 hours) in the order received.

### Procedure

All communication between the research agency and the participants took place on the web via WhatsApp. The day before the study was launched (day 0), the research agency distributed data packages to each of the participants to cover internet data costs incurred. Participants completed web-based self-report questionnaires hosted on Qualtrics at 3 time points, that is, baseline (day 1), 1 day after the intervention (day 9), and 1 month after the intervention (day 36). The items in the closed questionnaires were not randomized. Adaptive questioning was used only for the consent question at the beginning of each questionnaire. The questionnaires contained an average of 34 (SD 3.77) screens with 4 to 5 items per page. A “Back” button was included so that participants had the option of reviewing and changing their responses. Questionnaire functionality was tested and verified by the authors (KMG and SH) before sharing the Qualtrics links with the research agency.

At 8 AM (Coordinated Universal Time +7) on days 1 (time 1, baseline [T1]), 9 (time 2, 1 day after the intervention [T2]), and 36 (time 3, 1 month after the intervention [T3]), the research agency sent participants a link to the corresponding questionnaire as well as their unique participant identification number (PIN), which participants entered at the beginning of each questionnaire. The participants had 24 hours to complete each self-report assessment. Reminder messages were sent to participants who had not completed the questionnaire within the first 8 hours (these prompts will not be used or available when the intervention is disseminated on social media). Demographic information was collected using the baseline questionnaire.

After the participants completed the baseline questionnaire, a researcher not involved in this project was concealed from participant information and condition and generated the allocation sequence (based on a block design) using an automated web-based randomizer [48] to assign participants to the intervention or waitlist control group. The allocation was shared with the research agency, who made participants aware of which group they were in via WhatsApp, the day after completing the baseline questionnaire. Those in the intervention group were informed when to expect to receive the intervention; those in the control group were informed they would receive a link to the second self-report assessment in a week’s time. The research agency was not concealed from the participant’s randomized arm.

Participants randomized to the intervention condition were sent their PIN and a Qualtrics link with one video and its associated activities on days 3 to 8 at 8 AM (Coordinated Universal Time +7). Included in each link were state measures of body satisfaction and mood to be completed immediately before and after each of the intervention’s videos. Participants had 24 hours to engage with each link (ie, watch the video and complete its associated web-based activities), with reminder messages sent to those who had not engaged with the intervention after 8 hours. Acceptability information was obtained from the intervention group participants at the end of the day 9 questionnaire.

The day after the final questionnaire (day 37), all participants received a certificate of participation and debrief document that outlined the study’s objective, included contacts for mental health support, and were provided a link to access the intervention video series. Thereafter, incentives of Rp 125,000 (approximately US $8.75) were given to participants who completed all 3 questionnaires. Winners of the “Your Own Words” activities were awarded their prize within 2 weeks of trial completion. As specified in the trial protocol [46], the aforementioned process (with the exception of sending reminder messages to intervention participants on days 3 through 8; see *Pilot Study and Protocol Amendments* in the *Results* section) was first executed as an internal pilot with 150 participants to identify any procedural amendments necessary for the main trial.

### Measures

#### Primary Outcome Measure

The primary outcome measure for assessing trait body satisfaction was the Body Esteem Scale for Adolescents and Adults (BESAA) [49], which was validated on the web among young people in Indonesia (KM Garbett et al, unpublished data, December 2022). Three subscales (positive appearance, negative appearance, and weight) were administered and combined to form a total scale score. Higher scores indicate greater trait body satisfaction. The internal consistency at baseline was good (Cronbach α=.80).

#### Secondary Outcome Measures

Three secondary outcome measures were used. The internalization-general subscale of the Sociocultural Attitudes Towards Appearance Questionnaire (SATAQ-3) [50], validated on the web with Indonesian young people (KM Garbett et al, unpublished data, December 2022), was used. Higher scores indicate greater internalization of appearance ideals. The internal consistency was good (Cronbach α=.95). The Positive and Negative Affect Schedule for Children (PANAS-C) [51], also validated on the web with Indonesian adolescents (S Haywood et al, unpublished data, December 2022), was included to assess trait mood. The validated Indonesian version consists of 2 subscales similar to the original PANAS-C: a positive affect subscale, in which higher scores indicate greater positive mood, and a negative affect subscale, in which higher scores indicate greater negative mood. Internal consistency was good with Cronbach α=.90 for positive affect and Cronbach α=.88 for negative affect. A purpose-built measure to assess skin shade dissatisfaction was used, as no validated measure currently exists. Using a skin color chart depicting 9 shades from dark (1) to light (9) via The Pantone Skin Tone Guide [52], participants indicated the shade that best reflected their “current skin shade” and “ideal skin shade.” The skin shade dissatisfaction score was the absolute discrepancy between the 2 descriptions, which ranged from 0 (*satisfied with skin shade*) to 8 (*very dissatisfied with skin shade*).

#### State Outcome Measures

To assess state body satisfaction and mood, single-item measures on a 101-point visual analog scale [53] were used immediately before and after each of the intervention’s videos. Higher scores indicate greater body satisfaction and mood.

#### Intervention Acceptability

On day 9, the intervention condition participants received 6 self-report items at the end of the T2 questionnaire that assessed the intervention in relation to their overall enjoyment, likability of the characters, understandability, age appropriateness, usefulness, and likelihood of recommending the intervention to a peer. The response options ranged from 1 (*strongly disagree*) to 5 (*strongly agree*).

#### Adherence

The level of adherence to the intervention was assessed using various metrics that examined video and activity engagement. The following metrics were collected: the percentage of participants who watched each video and all 6 videos (calculated by the number of participants whose dwell time on the Qualtrics page hosting each video was equal to or longer than the video length), the average number of videos watched, the percentage of participants who completed each activity, the average number of activities completed, and the average amount of time participants engaged in the entire intervention (ie, all 6 videos and their corresponding activities).

### Sample Size

Similar randomized controlled trials assessing body dissatisfaction using the same outcome measure reported a range of small to medium standardized effect sizes, with Hedges *g* ranging from 0.25 to 0.4, exceeding the minimum important clinical differences [54]. To detect the minimum important clinical differences or larger, our proposed sample size of n=900 per group would provide >90% power (2-sided, Cronbach α=.05) for between-group differences at either T2 or T3. This assumes that the dropout rate does not exceed 20% in any 1 arm.

### Analyses

#### Overview

Analyses were performed using SPSS 28 (IBM Corp). Any duplicate questionnaire entries were identified by the participants’ unique PIN, and the first entry was retained for analysis. To avoid interpretation bias, condition allocation was concealed from the data analyst throughout data preparation and trait outcome hypothesis testing. However, the concealment of condition allocation was not possible during state outcome hypothesis testing because of the within-group design of this study. The data analyst was provided with the state-based measures data file only when the analyses of the trait-based measures were complete.

#### Hypotheses Testing and Post Hoc Analyses

##### Trait Outcomes

The effect of the intervention on trait outcomes was examined by running 4 linear mixed models (LMMs) on an intention-to-treat basis, with baseline measures at T1 as a covariate, randomized group as a 2-level between-subjects factor, study phase (T2 and T3) as a 2-level repeated measures factor, an unstructured covariance matrix, and the restricted maximum likelihood estimation method. The statistical model was hierarchically balanced, with one 3-way interaction among covariate, phase, and randomized group, three 2-way interactions (covariate × phase, covariate × randomized group, phase × randomized group), and 3 main effects (covariate, phase, and randomized group). For the effect size estimation, we calculated partial eta-squared for each model factor.

To estimate whether randomized group significantly contributed to the LMM, we ran four −2 log likelihood (LL) tests comparing the complete LMMs described earlier to LMMs without randomized group and its interactions. This model included baseline measures at T1 as a covariate, the study phase (T2 and T3) as a 2-level repeated measures factor, and one 2-way interaction (covariate × phase). For the −2 LL test, the 2 models were run using an unstructured covariance matrix and the maximum likelihood estimation method.

For each trait outcome, 2 preplanned analyses of covariance (ANCOVAs) were run to separately verify the effect of randomized group at T2 and T3. The models included baseline measures as a covariate, randomized group as a fixed factor, and measures at either T2 or T3 as dependent variables. The main conclusions were drawn from these preplanned ANCOVAs.

We also ran 2 post hoc repeated measures ANOVAs for each trait outcome, one for the control and one for the intervention group, testing for time trends across all 3 time points.

Finally, preplanned dose-response effects were tested in the intervention condition by running multiple regression analyses with each trait outcome at T2 and T3 as a dependent variable and Helmert-coded engagement scores (ie, the number of videos watched) as independent variables. Helmert coding allows the comparison of each level of engagement against the remaining higher levels, thereby identifying potential jump points in the data [55].

##### State Outcomes

For each state outcome, 6 dependent sample *t* tests (2-tailed) were run to compare the levels of state body satisfaction and mood immediately before and after watching each of the 6 intervention videos.

To test for cumulative effects across the 6 videos, we ran two 2 × 6 ([prevideo vs postvideo] × [6 videos]) fully repeated measures ANOVA for state body satisfaction and state mood, checking for linear, quadratic, cubic trends, and repeated contrasts. For both state measures, gain scores for each video were calculated (subtracting the prevideo state score from the postvideo state score), and then post hoc dependent sample *t* tests were run on adjacent gain scores. Finally, we conducted 2 post hoc repeated measures ANOVAs for each state outcome: one for the prevideo scores and one for the postvideo scores to check for time trends in the pre- and poststate scores separately.

##### Exploratory Analyses

After testing the hypotheses and post hoc analyses, we tested an exploratory mediation model with PROCESS Macro for SPSS (model 4) [56]. We calculated the gain score of internalization between T1 and T2 by subtracting internalization scores at posttest from internalization scores at pretest (with higher scores indicating a larger drop in internalization from preintervention to postintervention). In the model, randomized group was included as the independent variable, change in internalization from pretest to posttest as a mediator, trait body satisfaction at follow-up as a dependent variable, and trait body satisfaction at pretest as a covariate.

#### Data Preparation

The trait outcomes data set across conditions had 0.32% (356/112,667) missing item responses at T1, 4.31% (4858/112,667) missing item responses at T2, and 3.12% (3517/112,667) missing item responses at T3 (Multimedia Appendix 2; the formula for percentage of missing items: (N missing item responses for each scale at each time point / [total scale items × N participants] × 100).

Independent sample *t* tests confirmed that missing data for the primary outcome measure (BESAA) at T2 and T3 were not dependent on the baseline values of trait body satisfaction (t_1845_=0.93, 2-sided *P*=.35 at T2; t_1845_=1.0, *P*=.32 at T3). Chi-square analyses showed that missing data at either T2 or T3 did not differ significantly between the randomized arms (*χ*^2^_1_=3.48, 2-sided *P*=.06). Analysis using the Little Missing Completely at Random test indicated that missingness was consistent with data missing completely at random between T1 and T2 (*χ*^2^_1_=0.87, 2-sided *P*=.35) and between T1 and T3 (*χ*^2^_1_=1.0, 2-sided *P*=.31).

Moreover, the dropout rate never exceeded 5% between time points for any of the variables, supporting the conclusion that missing data were not concerning for this data set, given the high retention rates [57]. Participants who failed to complete at least 80% of the items on a given scale were omitted from the analyses for that particular scale. Attrition rates between time points were low, with a maximum loss of responses totaling 5.1% ([918/924] – [871/924]) between T1 and T2 for skin shade dissatisfaction in the intervention group. Some participants with missing responses at T2 provided complete responses at T3. Compared with T1, at follow-up, we observed a maximum loss of responses totaling 3.6% ([924/924] – [893/924]) for trait body satisfaction in the intervention group (Multimedia Appendix 3; the percentage represents a subtraction between fractions. The first fraction refers to the total number of participants who completed the questionnaire at T1 divided by the total number of participants randomized in the condition. The second fraction refers to the total number of participants who completed the questionnaire at either T2 or T3 divided by the total number of participants randomized in the condition).

For hypothesis testing, LMMs and post hoc ANCOVAs were conducted on an intention-to-treat basis without performing data imputation. As the percentage of missingness was <5%, LMMs and ANCOVAs were considered robust against a noncomplete data set [58]. Data imputation and per-protocol analyses were not conducted to avoid introducing bias in the distributions of the outcome variables with such a minimal percentage of missing data [59].

The LMMs and ANCOVAs assumptions of the linearity of residuals, continuous dependent variables, homogeneity of regression slopes, homogeneity of covariance matrices, and absence of collinearity were all met for the trait outcomes. The assumption of homoscedasticity was met for all trait outcomes, with only a slight violation of the internalization scores. Despite not meeting perfect normality, all residuals of the trait outcomes at T2 and T3 presented only minimal skewness (−2<skewness<+2) and minimal kurtosis (excess kurtosis <5). Given that ANCOVA is robust against the violation of the normal distribution of residuals when conducted with an appropriate sample size and groups of equal size, the data were neither transformed nor were outlier scores substituted [60].

Attrition rates were also low for state outcomes. For the intervention group only, missing responses on the 1-item state measures ranged between 12.1% (112‬/924; before video 1) and 13.8% (128/924; after video 6) for body satisfaction and between 12% (111‬/924; before video 1) and 14% (129/924; after video 6) for mood (Multimedia Appendix 4).

## Results

### Pilot Study and Protocol Amendments

Recruitment for the pilot study was conducted from September 13 to 16, 2021, and executed between September 18 and 26, 2021. A sample spread evenly across age and socioeconomic status (n=150) was obtained from the cities of Jakarta, Medan, and Semarang. As outlined in the trial protocol [46], we assessed the following criteria to guide our decision to progress to the main trial: participant retention at T1 and T2, intervention adherence (ie, viewing all 6 videos), data quality (ie, accurate completion of survey attention checks), and the assessment of harm (ie, change in trait body satisfaction in intervention participants between T1 and T2 compared with control participants). Using a traffic light system, the criteria were assessed and categorized as green (continue with the main trial), amber (consult the research team and make changes), or red (reconsider protocol or consider trial termination). The following 3 criteria were classified as green: participant retention was 98.7% (148/150); data quality was categorized as strong in that 97.3% (144/148) of participants correctly responded to all attention checks at T1 and T2; and, relative to the control condition, no indication of harm was witnessed across any outcome measure. Complete adherence to the intervention was 67% (49/73), which was categorized as amber. To increase intervention adherence in the main trial, reminder messages were incorporated on days 3 to 8 to those who had not completed the intervention 8 hours after receiving the intervention link. Pilot data were included in the main trial analysis, as no substantial changes were made to the study design or intervention. This process is encouraged to ensure that research funding is used both sparingly and efficiently [61].

### Main Trial

**Figure 1:**
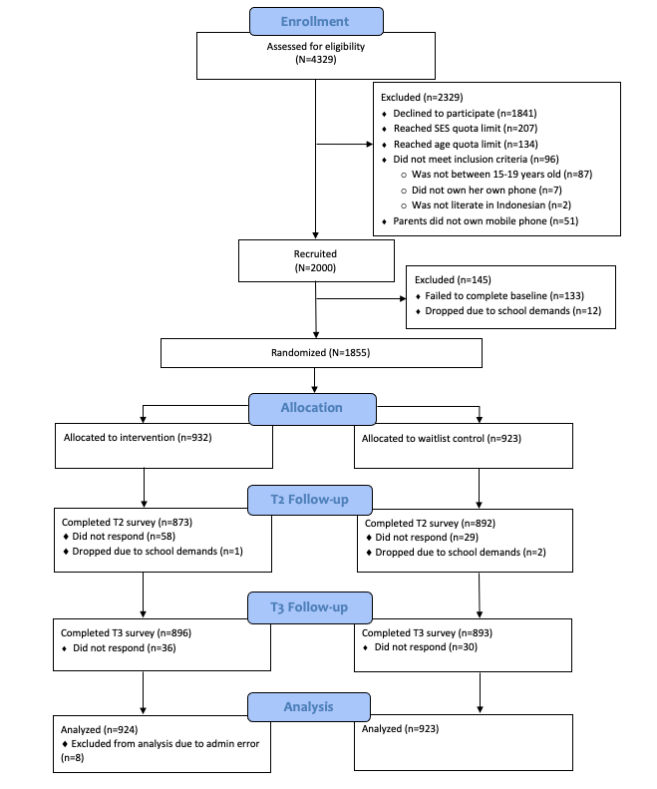
Participant flowchart. SES: socioeconomic status; T2: time 2, 1 day after the intervention; T3: time 3, 1 month after the intervention.

Recruitment was conducted between October 12 and November 5, 2021. The main trial was conducted from November 6 to December 12, 2021. The participant flow diagram for the main study is shown in Figure 1. Participants were aged between 15 and 19 years (intervention: mean 16.94, SD 1.40 years; control: mean 17, SD 1.41 years). There were no significant differences between the groups (t_1845_=0.87, 2-sided *P*=.38), and the sample was evenly stratified by age. The 2 groups were similar in terms of all other demographic characteristics. Most participants (1846/1857, 99.9%) were born in Indonesia. They were recruited from 10 Indonesian cities, with most participants (401/1847, 21.7%) based in Jakarta (Table 1). The most common ethnicity was Javanese (665/1847, 36%), followed by Sundanese (255/1857, 13.8%), which together accounted for half (920/1847, 49.8%) of the total sample; 7 other common ethnicities in Indonesia comprised one-fifth (399/1847, 21.6%) of the sample (Table 1 and Multimedia Appendix 5). Two-thirds of the participants (1166/1847, 63%) belonged to middle socioeconomic groups, followed by more than a quarter of the participants (511/1847, 27.7%) belonging to the upper socioeconomic groups (Table 1). Most participants (1691/1847, 91.9%) identified as Muslim (Islam religion; Table 1). The 2 groups did not differ in any of the trait outcome variables at baseline (Table 2). The mean completion time ranged between 19 and 39 minutes, with no significant difference between the groups at any time point (Multimedia Appendix 6). Therefore, we concluded that randomization was successful.

A power calculation showed that considering the total sample of 1847‬ participants, 3 time points, 5 outcomes, and a correlation among repeated measurements ranging between *r*=0.5 and *r*=0.8, we achieved a power ranging between 99% and 100% to detect small (partial η^2^=0.10) and medium (partial η^2^=0.6) effect sizes, considering a .05 α error (η^2^=0.01 indicates a small effect; η^2^=0.06 indicates a medium effect; and η^2^=0.14 indicates a large effect [62]). Therefore, the analyses were adequately powered.

**Table 1:** Participant baseline demographic data.

			Total (N=1847)		Control (n=923)		Intervention (n=924)
Age (years), mean (SD)			16.96 (1.38)		17 (1.41)		16.94 (1.40)
**Age (years), n (%)**							
	15	371 (20.1)		183 (19.8)		188 (20.3)	
	16	363 (19.7)		174 (18.9)		189 (20.5)	
	17	411 (22.3)		197 (21.3)		214 (23.2)	
	18	376 (20.4)		201 (21.8)		175 (18.9)	
	19	326 (17.7)		168 (18.2)		158 (17.1)	
**Country of birth, n (%)**							
	Indonesia	1846 (99.9)		922 (99.9)		924 (100)	
	Malaysia	1 (0.1)		1 (0.1)		0 (0)	
**City, n (%)**							
	Balikpapan	98 (5.3)		45 (4.9)		53 (5.7)	
	Bandung	259 (14)		140 (15.2)		119 (12.9)	
	Greater Jakarta	401 (21.7)		190 (20.6)		211 (22.8)	
	Makassar	102 (5.5)		48 (5.2)		54 (5.8)	
	Manado	94 (5.1)		48 (5.2)		46 (5)	
	Medan	176 (9.5)		90 (9.8)		86 (9.3)	
	Palembang	144 (7.8)		75 (8.1)		69 (7.5)	
	Pontianak	97 (5.3)		49 (5.3)		48 (5.2)	
	Semarang	219 (11.9)		109 (11.8)		110 (11.9)	
	Surabaya	257 (13.9)		129 (14)		128 (13.9)	
**Ethnicity, n (%)**							
	Bataknese	74 (4)		28 (3)		46 (5)	
	Betawi	84 (4.6)		46 (5)		38 (4.1)	
	Buginese	39 (2.1)		19 (2.1)		20 (2.2)	
	Javanese	665 (36)		344 (37.3)		321 (34.7)	
	Makassarese	47 (2.5)		23 (2.5)		24 (2.6)	
	Malay	53 (2.9)		26 (2.8)		27 (2.9)	
	Minangnese	34 (1.8)		17 (1.8)		17 (1.8)	
	Palembangese	68 (3.7)		30 (3.3)		38 (4.1)	
	Sundanese	255 (13.8)		123 (13.3)		132 (14.3)	
	Other ethnicity or ethnicities^a^	157 (8.5)		79 (8.6)		78 (8.4)	
	Did not know	140 (7.6)		79 (8.6)		61 (6.6)	
	Incorrect response (eg, city of residence)	148 (8)		65 (7)		83 (9)	
	Did not respond	83 (4.5)		44 (4.8)		39 (4.3)	
**Religion, n (%)**							
	Christianity, Adventist	2 (0.1)		1 (0.1)		1 (0.1)	
	Christianity, Catholicism	20 (1.1)		15 (1.6)		5 (0.5)	
	Christianity, Protestantism	126 (6.8)		69 (7.5)		57 (6.2)	
	Confucianism	2 (0.1)		1 (0.1)		1 (0.1)	
	Hindu	1 (0.05)		1 (0.1)		0 (0)	
	Islam	1691 (91.9)		834 (90.4)		857 (92.7)	
	Prefer not to say	5 (0.3)		2 (0.2)		3 (0.3)	
**Socioeconomic status, n (%)**							
	Lower I	167 (9)		87 (9.4)		80 (8.7)	
	Lower II	3 (0.2)		3 (0.3)		0 (0)	
	Middle I	854 (46.2)		408 (44.2)		446 (48.3)	
	Middle II	312 (16.9)		163 (17.7)		149 (16.1)	
	Upper I	158 (8.6)		79 (8.6)		79 (8.5)	
	Upper II	353 (19.1)		183 (19.8)		170 (18.4)	

^a^See Multimedia Appendix 5 for a complete reporting of ethnicity.

**Table 2:** Trait outcomes values for both groups at each time point.

Trait outcome measure			T1^a^									T2^b^					T3^c^		
			Control, mean (SD)		Int^d^, mean (SD)		*t* test (*df*)^e^		*P* value		Control, mean (SD)			Int, mean (SD)		Control, mean (SD)			Int, mean (SD)
**Primary outcome measure**																			
	BESAA^f^ (range 1-5)	3.46 (0.63)		3.45 (0.61)		0.29 (1845)		.77		3.46 (0.63)			3.43 (0.61)		3.44 (0.60)			3.49 (0.59)	
**Secondary outcome measure**																			
	Internalization subscale of SATAQ-3^g^ (range 1-5)	2.83 (0.82)		2.85 (0.83)		−0.62 (1843)		.53		2.82 (0.77)			2.66 (0.84)		2.84 (0.75)			2.65 (0.78)	
	Positive affect subscale of PANAS-C^h^ (range 1-5)	3.74 (0.67)		3.75 (0.66)		−0.03 (1823)		.97		3.73 (0.70)			3.70 (0.67)		3.72 (0.70)			3.74 (0.67)	
	Negative affect subscale of PANAS-C^h^ (range 1-5)	2.87 (0.61)		2.89 (0.62)		−0.97 (1833)		.33		2.76 (0.65)			2.81 (0.66)		2.83 (0.67)			2.81 (0.66)	
	Skin shade dissatisfaction (range 0-8)	1.34 (1.17)		1.29 (1.10)		0.94 (1835)		.35		1.28 (1.10)			1.14 (1.02)		1.24 (1.06)			1.16 (1.02)	

^a^T1: time 1, baseline.

^b^T2: time 2, 1 day after the intervention.

^c^T3: time 3, 1 month after the intervention.

^d^Int: intervention group.

^e^2-tailed.

^f^BESAA: Body Esteem Scale for Adolescents and Adults.

^g^SATAQ: Sociocultural Attitudes Towards Appearance Questionnaire.

^h^PANAS-C: Positive and Negative Affect Schedule for Children.

### Adherence

Intervention adherence metrics were also assessed. On average, intervention participants watched 5 of the 6 videos and completed 14 of the 18 activities. See Multimedia Appendix 7 for the adherence to each video and activity. It was not possible to accurately calculate the average amount of time that intervention participants engaged with the entire intervention, as time spent on the intervention for each of the 6 videos for many participants (n=401) exceeded an hour, suggesting that participants did not close the survey tab after watching the video and completing the corresponding activities.

### Hypotheses Testing and Post Hoc Analyses

#### Trait Outcomes

##### Body Satisfaction

The LMM with baseline body satisfaction as a covariate, randomized group as a 2-level between-subjects factor, study phase (T2 and T3) as a 2-level repeated measures factor, three 2-way interactions, and one 3-way interaction found a nonsignificant effect of randomized group (*P*=.26), a nonsignificant group-by-time interaction (*P*=.26), and a significant effect of time (*P*<.001). The −2 LL test comparing the full model and the model without the effect of randomized group and its interaction was significant (*P*<.001), indicating that randomized group significantly contributed to the model (Multimedia Appendix 8).

**Figure 2:**
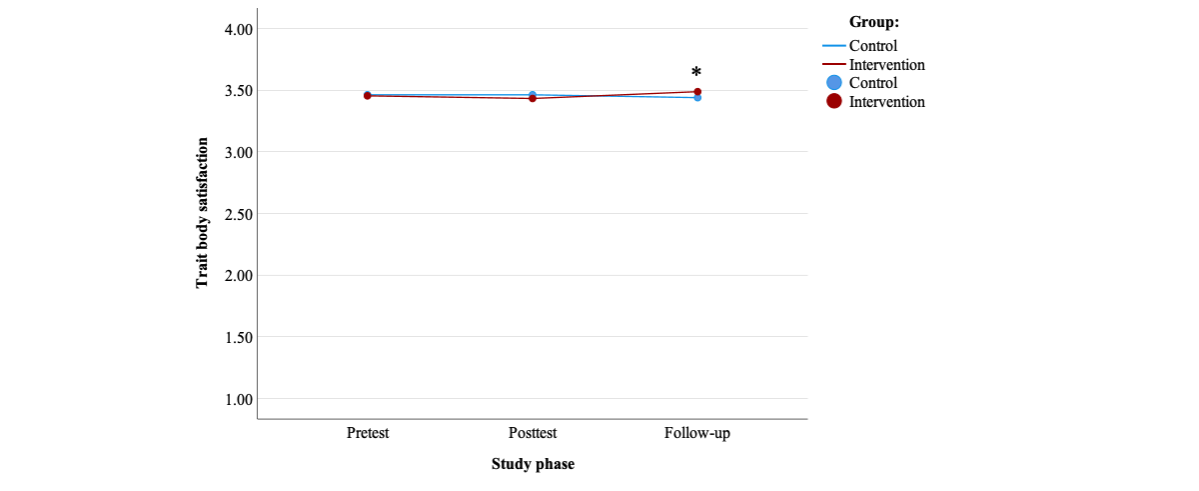
Trait body satisfaction over time for both groups. *Significant analysis of covariance effect of the randomized group at a specific time point.

The preplanned ANCOVAs showed a nonsignificant effect of randomized group on mean body satisfaction at T2 (*F*_1,1760_=0.388, *P*=.53, partial η^2^=0.000) when controlling for body satisfaction at T1 (*F*_1,1760_=2694.31, *P*<.001, partial η^2^=0.605). However, the ANCOVA showed a significant effect of randomized group on mean body satisfaction at T3 (*F*_1,1781_=9.02, *P*=.005, partial η^2^=0.005) when controlling for body satisfaction at T1 (*F*_1,1781_=1868.83, *P*<.001, partial η^2^=0.512), and the intervention group showed significantly higher levels of body satisfaction when compared with the control group (Table 2; Figure 2). The effect sizes for randomized group were small.

The post hoc repeated measures ANOVAs showed a nonsignificant effect of time on body satisfaction in the control group (*F*_2, 870_=1.56, *P*=.20, partial η^2^=0.004), whereas it showed a significant effect in the intervention group (*F*_2, 851_=9.24, *P*<.001, partial η^2^=0.021), confirming the previous results. In particular, in the intervention group, the post hoc repeated measures ANOVA showed a significant increase in mean body satisfaction between T1 and T3 (95% CI −0.074 to −0.011) as well as between T2 and T3 (95% CI −0.076 to −0.028), in line with previous analyses (Table 2).

##### Internalization

The LMM with baseline internalization as a covariate, randomized group as a between-subjects factor, study phase (T2 and T3) as a within-subjects factor, three 2-way interactions, and one 3-way interaction found a nonsignificant effect of group (*P*=.16), a significant group-by-time interaction (*P*=.03), and a significant effect of time (*P*<.001). The −2 LL test was significant (*P*<.001), indicating that randomized group significantly contributed to the model (Multimedia Appendix 8).

**Figure 3:**
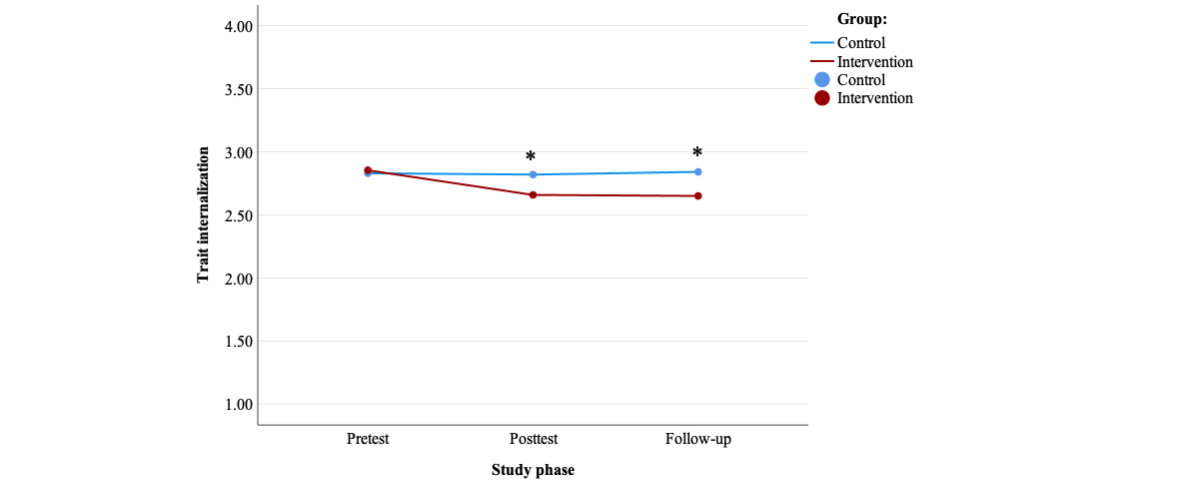
Trait internalization over time for both groups. *Significant analysis of covariance effect of the randomized group at a specific time point.

The preplanned ANCOVAs showed a significant effect of randomized group on internalization both at T2 (*F*_1,1758_=40.56, *P*<.001, partial η^2^=0.022) and T3 (*F*_1,1782_=54.03, *P*<.001, partial η^2^=0.03) while controlling for body satisfaction levels at T1 (*F*_1,1758_=1514.30, *P*<.001, partial η^2^=0.46; *F*_1,1782_=1332.45, *P*<.001, partial η^2^=0.428). The intervention group showed lower levels of internalization at T2 and T3, compared with the control group (Table 2; Figure 3). The effect sizes for randomized group were small.

In line with previous results, the post hoc repeated measures ANOVAs showed no significant effect of time in the control group (*F*_2,871_=0.58, *P*=.56, partial η^2^=0.001), whereas it showed a significant effect in the intervention group (*F*_2,849_=41.24, *P*<.001, partial η^2^=0.09). In particular, the post hoc repeated measures ANOVAs showed a significant decrease in mean internalization between T1 and T2 (95% CI 0.15-0.24) as well as between T1 and T3 (95% CI 0.15-0.25) in the intervention condition (Table 2).

##### Skin Shade Dissatisfaction

The complete LMM conducted on skin shade dissatisfaction showed a nonsignificant effect of group (*P*=.25), a nonsignificant effect of group-by-time interaction (*P*=.66), and a significant effect of time (*P*=.07). The −2 LL test comparing the models with and without randomized group was significant (*P*=.03), indicating that randomized group significantly contributed to explaining the variance in the dependent variable (Multimedia Appendix 8).

**Figure 4:**
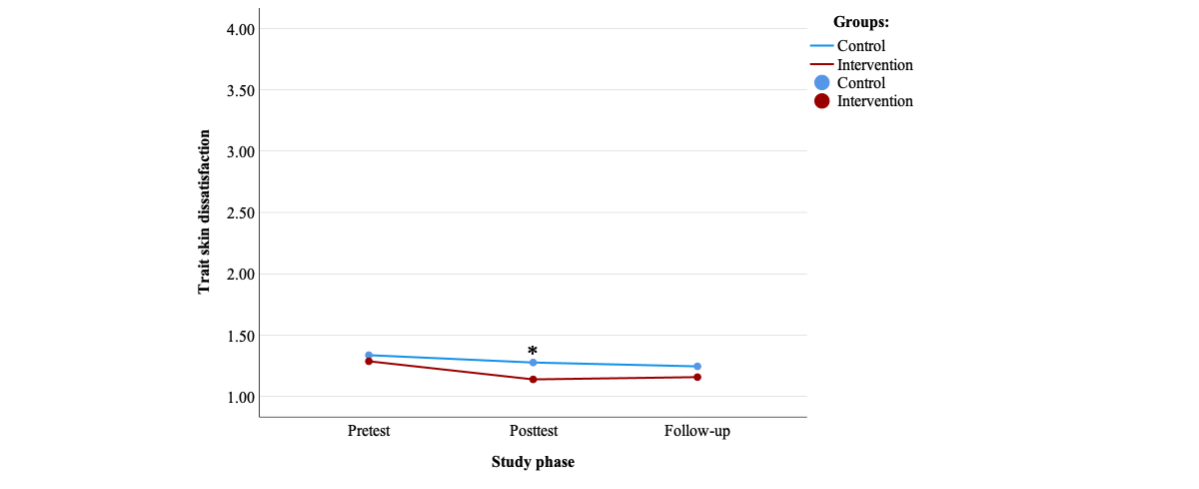
Trait skin shade dissatisfaction over time for both groups. *Significant analysis of covariance effect of the randomized group at a specific time point.

The preplanned ANCOVAs showed a significant effect of randomized group on skin shade dissatisfaction at T2 (*F*_1,1744_=8.05, *P*=.005, partial η^2^=0.005) while controlling for T1 scores (*F*_1,1744_=813.27, *P<*.001, partial η^2^=0.318), and the intervention group showed lower levels of skin shade dissatisfaction when compared with the control group (Table 2; Figure 4). The effect sizes for randomized group were small. Randomized group did not have a significant effect at T3 (*F*_1,1771_=2.77, *P*=.09, partial η^2^=0.002) when controlling for T1 scores (*F*_1,1771_=640.36, *P*<.001, partial η^2^=0.266).

The post hoc repeated measures ANOVAs showed no significant effect of time in the control group (*F*_2,861_=2.13, *P*=.12, partial η^2^=0.005) and a significant effect in the intervention group (*F*_2,844_=10.51, *P*<.001, partial η^2^=0.024). In the intervention group, the post hoc repeated measures ANOVA showed a significant reduction in skin shade dissatisfaction between T1 and T2 (95% CI 0.08-0.22), as well as between T1 and T3 (95% CI 0.06-0.20; Table 2).

##### Negative Mood

The LMM with baseline negative mood as a covariate, randomized group as a 2-level between-subjects factor, study phase (T2 and T3) as a 2-level repeated measures factor, three 2-way interactions, and one 3-way interaction showed a nonsignificant effect of group (*P*=.97), as well as a nonsignificant group-by-time interaction (*P*=.51), and a significant effect of time (*P*=.41). The −2 LL test comparing the full model and the model without the effect of randomized group and its interaction was nonsignificant (*P*=.06), indicating that randomized group did not significantly contribute to the model (Multimedia Appendix 8).

The preplanned ANCOVAs confirmed the previous results, finding a nonsignificant effect of randomized group on negative mood at T2 (*F*_1,1742_=1.47, *P*=.23, partial η^2^=0.001) and T3 (*F*_1,1772_=1.85, *P*=.17, partial η^2^=0.001) when controlling for T1 scores (*F*_1,1742_=1367.26, *P*<.001, partial η^2^=0.440; *F*_1,1772_=1316.09, *P*<.001, partial η^2^=0.426).

The post hoc repeated measures ANOVAs showed a significant effect of time in the control group (*F*_2,865_=19.32, *P*<.001, partial η^2^=0.043), as well as a significant effect in the intervention group (*F*_2,840_=10.70, *P*<.001, partial η^2^=0.025). In particular, the post hoc repeated measures ANOVA showed a significant reduction in negative mood between T1 and T2 (95% CI 0.07-0.14), as well as between T1 and T3 (95% CI 0.001-0.072) in the control group. A similar significant reduction in negative mood was shown between T1 and T2 (95% CI 0.04-0.11) as well as between T1 and T3 (95% CI 0.04-0.11) in the intervention group (Table 2).

##### Positive Mood

The LMM conducted on positive mood showed a nonsignificant effect of group (*P*=.33), a nonsignificant effect of time (*P*<.001), and a nonsignificant group-by-time interaction (*P*=.24). The −2 LL test was also nonsignificant (*P*=.41), indicating that randomized group did not significantly contribute to the model (Multimedia Appendix 8).

The preplanned ANCOVAs confirmed these results, finding a nonsignificant effect of randomized group on positive mood at both T2 (*F*_1,1734_=0.61, *P*=.43, partial η^2^=0.000) and T3 (*F*_1,1758_=1.20, *P*=.27, partial η^2^=0.001) while controlling for T1 scores (*F*_1,1734_=1665.95, *P*<.001, partial η^2^=0.490; *F*_1,1758_=1306.27, *P*<.001, partial η^2^=0.426). The post hoc repeated measures ANOVAs showed no significant effect of time for the control group (*F*_2,860_=0.82, *P*=.44, partial η^2^=0.002) or the intervention group (*F*_2,834_=1.67, *P*=.18, partial η^2^=0.004), confirming previous results.

##### Dose-Response Analyses

Dose-response effects on trait outcomes at T2 and T3 in the intervention group were examined by running multiple regression analyses with Helmert-coded engagement scores (ie, the number of videos watched) as independent variables. The analyses showed no dose-response for all trait outcomes at any time point: body satisfaction at T2 (*F*_5,865_=0.894, *P*=.48) and T3 (*F*_5,887_=0.665, *P*=.65); internalization at T2 (*F*_5,864_=1.62, *P*=.15) and T3 (*F*_5,887_=0.95, *P*=.45); skin shade dissatisfaction at T2 (*F*_5, 859_=1.4, *P*=.23) and T3 (*F*_5,884_=1.07, *P*=.38); negative mood at T2 (*F*_5,860_=0.424*, P*=.83) and T3 (*F*_5,886_=0.64, *P*=.67); and positive mood at T2 (*F*_5,859_=1.65, *P*=.14) and T3 (*F*_5,884_=1.56, *P*=.17).

#### State Outcomes

##### State Body Satisfaction

**Figure 5:**
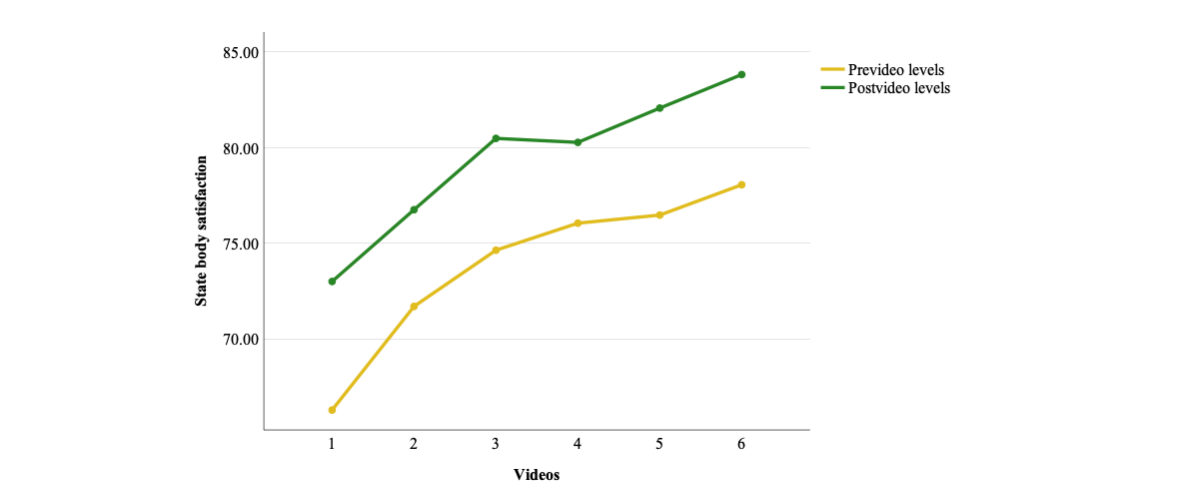
Plotted means for state body satisfaction in the intervention group with prevideo and postvideo means for each video.

Six dependent sample *t* tests compared levels of state body satisfaction before and immediately after watching each of the 6 *Warna-Warni Waktu* videos. The *t* tests revealed that each video was successful at increasing state body satisfaction in the intervention group (Table 3; Figure 5).

Cumulative analyses showed a significant interaction effect in the 2 × 6 ANOVA (*P*<.001), indicating that the magnitude of improvement in body satisfaction differed between videos. The interaction term also exhibited a significant quadratic trend (*P*=.003) (Multimedia Appendix 9). Post hoc dependent sample *t* tests comparing pre-post gain scores between adjacent time points showed that improvements in body satisfaction were significantly larger for videos 1 (*P*=.004) and 3 (*P*=.050) than for video 2. Similarly, body satisfaction improvements were significantly larger for videos 3 (*P*<.001) and 5 (*P*=.006) compared with video 4. Video 6 and 5 showed nonsignificant differences in pre-post gain scores (*P*=.432) (Multimedia Appendix 9).

The repeated measures ANOVA on prevideo scores was significant (*P*<.001), and repeated contrasts showed a significant and progressive improvement in prevideo body satisfaction scores as participants proceeded through the videos (Table 3; Multimedia Appendix 9; Figure 5). Similarly, the repeated measures ANOVA on postvideo scores was significant (*P*<.001), also finding a progressive improvement in postvideo body satisfaction scores as participants proceeded through the intervention, apart from a plateau between videos 3 and 4 (Table 3; Multimedia Appendix 9; Figure 5).

**Table 3:** State outcomes.

Video			Before, mean (SD)^a^		After, mean (SD)		Paired-sample *t* test (*df*)^a^			*P* value	
**State body satisfaction**											
	1	66.71 (23.61)		73.65 (21.47)		−12.75 (811)			<.001		
	2	72.17 (21.40)		77.11 (19.72)		−13.32 (801)			<.001		
	3	74.87 (19.94)		80.62 (18.43)		−15.40 (799)			<.001		
	4	76.17 (19.52)		80.18 (18.62)		−12.82 (798)			<.001		
	5	76.97 (20.10)		82.20 (18.38)		−15.07 (796)			<.001		
	6	77.92 (19.98)		83.61 (18.11)		−15.47 (795)			<.001		
**State mood**											
	1	69.45 (23.67)		73.76 (22.60)				−10.04 (812)			<.001
	2	71.35 (23.16)		75.10 (22.69)				−9.97 (801)			<.001
	3	73.92 (22.22)		78.30 (21.18)				−12.87 (798)			<.001
	4	74.46 (21.89)		78.54 (21.27)				−12.34 (798)			<.001
	5	75.16 (22.04)		79.64 (21.18)				−13.33 (796)			<.001
	6	75.66 (22.49)		81.42 (20.60)				−13.79 (794)			<.001

^a^2-tailed.

##### State Mood

**Figure 6:**
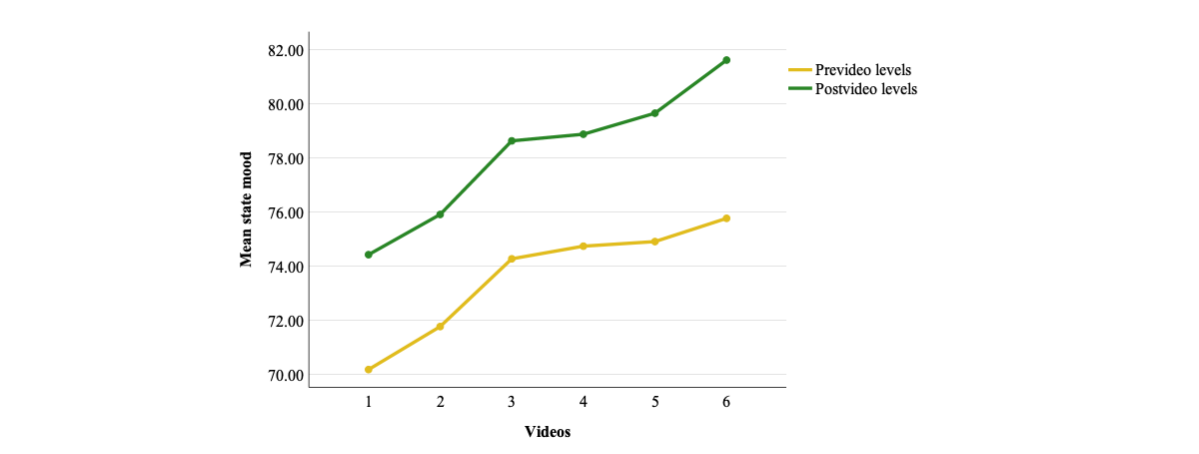
Plotted means for state mood in the intervention group with prevideo and postvideo means for each video.

Six dependent sample *t* tests comparing levels of state mood immediately before and after watching each of the 6 videos showed that each video was successful in increasing state mood in the intervention group (Table 3; Figure 6).

Cumulative analyses showed a significant interaction effect (*P*<.001) in the 2 × 6 ANOVA, suggesting that the magnitude of change in mood differed between videos. The interaction term also showed a significant quadratic (*P*<.001) and cubic (*P*=.016) trend (Multimedia Appendix 9). Post hoc dependent sample *t* tests comparing pre-post gain scores between adjacent time points were all nonsignificant, except for video 6, which showed significantly larger improvements in mood when compared with video 5 (*P*=.007) (Multimedia Appendix 9).

The repeated measures ANOVA on prevideo scores was significant (*P*<.001), and repeated contrasts showed a significant and progressive improvement in prevideo mood scores for videos 1, 2, and 3 (Table 3; Multimedia Appendix 9; Figure 6). The repeated measures ANOVA on postvideo scores was also significant, finding a similar progressive and significant improvement in postvideo state mood scores for all videos, apart from a plateau between videos 3 (*P*=0.63) and 5 (*P*=0.12) (Table 3; Multimedia Appendix 9; Figure 6).

### Exploratory Analyses

On the basis of these findings, exploratory analyses were conducted to test whether the observed delayed effect on trait body satisfaction could be mediated by the immediate effect that the intervention had on internalization. Such a finding would provide support for the relationship between these variables as defined in the Tripartite Influence Model [44], the theoretical underpinning of *Warna-Warni Waktu*. The exploratory mediated model with randomized group as a dichotomous independent variable, body satisfaction at T1 as a covariate, change in internalization from T1 to T2 as a mediator, and body satisfaction at T3 as a dependent variable was significant (*R*^2^=.54, *F*_3,1719_=680.58, *P*<.001).

**Figure 7:**
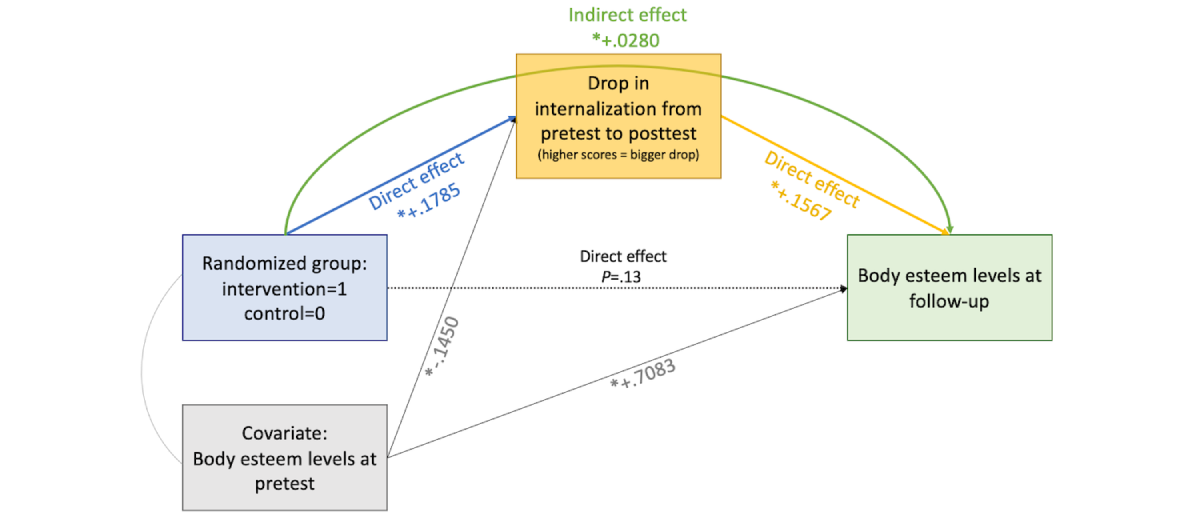
Exploratory mediation analysis. *Significant effect (*P*<.001) of the specific effect. For significant effects, the β is reported in the figure. For nonsignificant effects, the *P* value is reported in the figure.

Randomized group was significantly associated with the mediator (*R*^2^=.04, *F*_2,1720_=34.25, β=.18, *P*<.001), and participants in the intervention condition experienced a significantly larger drop in internalization between T1 and T2 compared with the control group. The covariate (body satisfaction at T1) also significantly predicted the mediator (β=−.1450, *P*<.001). The mediator significantly predicted the dependent variable (β=.16, *P*<.001), with participants experiencing a greater drop in internalization from T1 to T2 also experiencing higher body satisfaction at T3. The covariate also significantly predicted the dependent variable (β=.71, *P*<.001; Figure 7).

The model resulted in complete mediation, with the indirect effect of randomized group on body satisfaction at T3 being positive and significant (β=.03, 95% CI 0.017-0.041), and the direct effect was nonsignificant (β=.03, *P*=.13; Figure 7). Overall, this mediated model indicates that participants in the intervention condition experienced significantly higher body satisfaction at T3, which was completely mediated by the drop in internalization between T1 and T2 attributable to the intervention.

## Discussion

### Principal Findings

*Warna-Warni Waktu*, a social media–based intervention aimed at improving body image among adolescent girls and young Indonesian women, significantly improved trait body satisfaction and reduced internalization of appearance ideals and skin shade dissatisfaction relative to a waitlist control condition. The intervention showed no impact on trait mood relative to the waitlist control condition. State improvements in body satisfaction and mood were evident in participants in the intervention condition immediately after watching each video.

### Intervention Effectiveness on Trait Outcomes

Scalable, cost-effective, and evidence-based interventions to curb body dissatisfaction are necessary and in demand among adolescent girls and young Indonesian women [41]. *Warna-Warni Waktu* meets this need, providing an accessible, web-based, and culturally appropriate option for adolescent girls and young women in Indonesia to learn ways to resist the appearance pressures faced in their everyday lives. *Warna-Warni Waktu*, developed by local and international body image experts, industry leaders in creating body image content for young people, creative and digital agencies based in Jakarta and the United Kingdom, and young Indonesian women, has already shown strong acceptability among the target audience [46].

In this study, we found *Warna-Warni Waktu* significantly improved the primary outcome measure of trait body satisfaction 1 month after intervention participation, significantly reduced internalization of appearance ideals at 1 day and 1 month after the intervention, and significantly reduced skin shade dissatisfaction at 1 day after the intervention relative to a waitlist control condition. The magnitude of effect sizes for each of these positive outcomes was small, in line with previous research. Specifically, body image interventions of similar length designed for universal samples also have small effect sizes [5,7]. In body image research, small effects of universal samples are of considerable practical significance. Research, albeit limited among Indonesian samples, indicates that over half of young people globally experience low levels of body dissatisfaction [1,63,64]. Thus, the development and dissemination of scalable interventions to alleviate minor concerns as part of a stepped-care approach is an imperative step forward, particularly among those in LMICs.

Interestingly, the impact of *Warna-Warni Waktu* on trait body satisfaction was only evident at 1 month after engagement with the intervention, rather than at both 1 day and 1 month after the intervention, as hypothesized. Exploratory analyses of this pattern of findings, based on the intervention’s underpinning theory, provide a useful explanation. Specifically, as per the Tripartite Influence Model [44], mediation analyses explored whether the delayed impact on body satisfaction could be mediated by the intervention’s immediate impact on the internalization of appearance ideals. This was found to be the case; the impact of the intervention on body satisfaction at 1-month follow-up was completely mediated by the change scores in internalization between baseline and 1-day follow-up. Delayed or improved intervention effects over time on body image measures are documented in the literature [65-67]. By exploring the mediation effect of body image risk factors (eg, internalization of appearance ideals) in explaining these delayed effects with complete mediation effects witnessed, our findings contribute to an important conversation around delayed intervention impacts and highlight the importance of follow-up measures.

In addition to the role that internalization played as a mediator of body satisfaction, our hypothesis that internalization as an outcome would decrease at 1 day and 1 month after the intervention was confirmed, reinforcing that the intervention successfully targets this key risk factor. Similarly, skin shade dissatisfaction decreased at 1 day after the intervention, as predicted; however, these effects were not maintained at 1-month follow-up. This might be attributed to the purpose-built measure we used, given the lack of an appropriate validated measure of skin shade dissatisfaction. In addition, it could be that our intervention did not have sufficient potency to deliver sustained change given how deeply embedded skin shade dissatisfaction is among girls and women in Indonesian society.

Contrary to the hypotheses, the intervention had no significant impact on positive or negative trait mood. This may be because *Warna-Warni Waktu* was specifically designed to target risk factors for body dissatisfaction, not mood. Similar results have been found in other body image interventions [8,15,54], suggesting that greater consideration of the risk factors for low trait mood is required during intervention development to instigate a significant and sustained shift.

Finally, some exploratory post hoc analyses (see Multimedia Appendix 10 for a complete overview) showed that participants in the intervention group with lower levels of body satisfaction at baseline presented a significantly larger improvement in body satisfaction from pretest to posttest, as well as from pretest and follow-up, when compared with participants with higher levels of body satisfaction at baseline. Similarly, participants with lower levels of body satisfaction at baseline showed larger drops in internalization both between pretest and posttest, as well as from pretest to follow-up when compared with participants with higher levels of body satisfaction at baseline. These exploratory post hoc results suggest the potential presence of “ceiling effects” for participants who entered the intervention with relatively high levels of body satisfaction. In contrast, these results also suggest potentially higher intervention efficacy for adolescent girls and young women experiencing lower levels of body satisfaction.

### Dose-Response Effects

Intervention adherence was excellent, with intervention participants having watched an average of 5 out of the 6 videos and completed, on average, 14 of the 18 activities. These adherence results are much higher than those often observed in web-based psychological interventions [68]. High adherence rates may be owing to gender, with women more likely to adhere than men [68]; the high acceptability and likeability of the intervention among the target group [46]; the end users’ involvement in the development of *Warna-Warni Waktu,* which has been associated with strong adherence in other eHealth interventions [69,70]; and strong “buy in” from participants because of their familiarity with the research agency and financial incentives for the completion of other aspects of the study. Although high adherence is ideal to test the effectiveness of an intervention, it hampers the ability to run reliable dose-response effects. The heavily skewed distribution toward high levels of engagement resulted in a lack of power when comparing participants based on the number of videos watched [71]. Even though we found no dose-response effects on trait outcomes, it is important to note that nonsignificant findings may be owing to the lack of variation in engagement scores in the intervention condition.

### Intervention Effectiveness on State Outcomes

Overall, the effectiveness of *Warna-Warni-Waktu* on state outcomes was good, in that every video resulted in an immediate and significant increase in state body satisfaction and mood, indicating that each video positively impacted the viewer, however briefly.

Gain score comparisons within cumulative analyses showed that state-based improvements in body satisfaction were particularly strong for videos 1 (setting the scene), 3 (targeting appearance-based comparisons), 5 (targeting self-body talk), and 6 (concluding the story), and for state mood, video 6. Interestingly, of the 4 videos targeting specific risk factors for body dissatisfaction (videos 2-5), the 2 that targeted internal cognitive changes were the most effective at generating state change in body satisfaction. This may be because participants felt they could take immediate control of their thought processes to combat these risk factors for body dissatisfaction (ie, they had learned how to stop the chain of comparisons and view their body more compassionately in the moment), whereas the skills learned in videos 2 (targeting media literacy) and 4 (targeting appearance-based teasing) may take longer to put into practice and benefit from. Video 6 was also particularly effective in improving body satisfaction and mood. This final video presents an alternative world to the dystopian future seen in video 1, where society lives free from appearance pressures owing to the everyday positive changes made by just one individual (ie, Putri). Therefore, it is perhaps unsurprising that this new reality boosts both body satisfaction and mood among the audience, as it showcases the impact of individual-level changes.

It is noteworthy that we also found significant and progressive improvements in pre- and poststate body satisfaction scores for videos 1 to 6 (with a plateau between videos 4 and 5 for prevideo scores and a plateau between videos 3 and 4 for postvideo scores). Similarly, we observed a progressive increase in pre- and poststate mood scores, specifically between videos 1 and 3 for prevideo scores, which were maintained until video 6, and between videos 1 and 6, with a plateau between videos 3 and 5 for postvideo scores. A possible reason for the state score increases before each video could be that after watching the first video, participants felt progressively more invested in the narrative of *Warna-Warni Waktu* and identified with the struggle of the main character. This might have led participants to experience a boost in body satisfaction and mood, as they anticipated watching each video. The results suggest that this anticipation effect became stronger as participants progressed through the series. Similarly, we observed progressively higher postvideo scores in body image and mood as participants watched the series, which was also potentially linked to familiarization and investment with the characters and storyline. Research related to entertainment-education serial dramas has found that they positively affect attitudinal and behavioral health change, partly attributable to the emotions elicited by the story, repetition that facilitates identification with characters, and the characters’ conversion into role models for the viewer [72]. Furthermore, the extended elaboration likelihood model of persuasion posits that character identification and storyline engagement are key elements in having viewers process and incorporate the intended messages [73,74], which may explain the progressive and cumulative positive effects observed in our study.

### Strengths

Notable strengths of our study include excellent participant retention and intervention adherence, which are unusual for eHealth intervention trials [31], thus reinforcing the validity of the findings. In addition, as *Warna-Warni Waktu* was designed for delivery via social media, it engages adolescent girls and young Indonesian women in a familiar environment where they spend considerable time on a regular basis, so those in need are not required to access an unfamiliar platform or download an app, which is known to contribute to high attrition [31]. The scalability of health interventions in LMICs has been challenging and limited to small-scale interventions, primarily owing to a lack of a clear strategy for scalability before dissemination [75]. The multidisciplinary collaborative development in conjunction with adolescent girls and young Indonesian women and subsequent evaluation of *Warna-Warni Waktu* was grounded in the overarching goal of large-scale dissemination; our initial plan to reach hundreds of thousands of adolescent girls and young women in Indonesia in 2022 via social media marketing is a clear strength of this work.

### Limitations

There are a few key areas that future research could explore. First, funding constraints prevented us from evaluating the independent impact of the intervention’s videos versus the combined impact of videos and activities. This is an area for future research. Second, although social comparisons are a potentially important change mechanism in this work, as per the Tripartite Influence Model [44], no relevant measures presently exist that have been validated among Indonesian adolescents or young adults. Therefore, it would be advantageous for an appropriate measure, such as the revised Physical Appearance Comparison Scale [76] or the Upward and Downward Appearance Scales [77], to be validated in the Indonesian context and incorporated into a replication study. Third, it would be useful to understand the individual impact of each video; however, owing to our study’s excellent adherence, it was not possible to identify dose-response effects. Fourth, as there was no appropriate active control option, a waitlist control group was used; thus, it was not possible to conceal participants from their condition. Should a similar and culturally appropriate intervention be developed in Indonesia, it would be useful to replicate our study by using an active control. Fifth, given that participants were recruited via parents who had already been involved and interested in research (ie, via the research agency’s established research panels), there is a risk of selection bias; the parents and daughters by extension were self-selected as opposed to a randomly generated sample. Sixth, the findings at 1-month follow-up for *Warna-Warni Waktu* were encouraging. This follow-up time period is typical of body image intervention evaluations given the duration of the effects typically seen [5,14,54,78]. However, a replication study with a longer follow-up would be advantageous to determine the possible long-term effects *Warna-Warni Waktu* generates. Finally, despite accurately reproducing the intervention’s activities as they would be presented on social media to collect adherence data, our study lacks ecological validity, as we did not evaluate *Warna-Warni Waktu* on the social media platforms where it will be disseminated (ie, Facebook, Instagram, and YouTube). Evidence suggests that increased intervention adherence and an additive impact of shared learning can occur when interventions are disseminated on social media [79,80]. Furthermore, the role of friends on social networking sites has been shown to increase dissemination and act as positive reinforcement for eHealth interventions [30,81], further supporting the future evaluation of *Warna-Warni Waktu* in the environment where it will be delivered to the target group.

### Conclusions

This parallel randomized controlled trial showed that *Warna-Warni Waktu* is an effective, scalable intervention that can be implemented on social media to improve body satisfaction among adolescent girls and young Indonesian women. To our knowledge, this social media–based intervention is the first eHealth intervention of its kind. As such, it can act as a blueprint for intervention development in other LMIC contexts where face-to-face and one-to-one provision is lacking but internet penetration is high. Given that effect sizes were small, *Warna-Warni Waktu* should not be considered a panacea for Indonesian adolescent girls and young women who experience severe body image–related issues and may require more intensive treatment. Rather, our intervention should be regarded as an effective tool that can be used in conjunction with other interventions aimed at addressing body image concerns in Indonesian adolescent girls and young women.

## Appendix

Multimedia Appendix 1 Bahasa Indonesia translation of “Evaluating the Efficacy of a Social Media–Based Intervention (Warna-Warni Waktu) to Improve Body Image Among Young Indonesian Women: Parallel Randomized Controlled Trial”.

Multimedia Appendix 2 Missing items for each trait outcome per condition at each time point.

Multimedia Appendix 3 The number of participants who completed at least 80% of the items on each trait measure.

Multimedia Appendix 4 Retention rates for state outcomes in the intervention group (n=924).

Multimedia Appendix 5 Complete participant ethnicity data.

Multimedia Appendix 6 Number of minutes to complete each time point survey.

Multimedia Appendix 7 Intervention adherence: videos viewed and activities completed.

Multimedia Appendix 8 Trait outcomes as per linear mixed models executed with 21 dimensions and an unstructured covariance structure.

Multimedia Appendix 9 Cumulative analyses for state outcomes.

Multimedia Appendix 10 Exploratory post hoc analyses tested the differential effect of the intervention on participants with higher and lower levels of body satisfaction at baseline.

Multimedia Appendix 11 CONSORT-eHEALTH checklist V 1.6.1.

## Abbreviations

ANCOVA: analysis of covariance
BESAA: Body Esteem Scale for Adolescents and Adults
LL: log likelihood
LMIC: lower- and middle-income country
LMM: linear mixed model
PANAS-C: Positive and Negative Affect Schedule for Children
PIN: participant identification number
SATAQ-3: Sociocultural Attitudes Towards Appearance Questionnaire
T1: time 1, baseline
T2: time 2, 1 day after the intervention
T3: time 3, 1 month after the intervention
