# Supplementary material for: Evaluating the Efficacy of a Social Media–Based Intervention (Warna-Warni Waktu) to Improve Body Image Among Young Indonesian Women: Parallel Randomized Controlled Trial
Source: J Med Internet Res. 2023 Apr 3;25:e42499. doi: 10.2196/42499 (PMC10131926; doi:10.2196/42499)
Supplement: Multimedia Appendix 1 [file jmir_v25i1e42499_app1.pdf]

Multimedia Appendix 1. Bahasa Indonesia translation of "Evaluating the Efficacy of a Social Media–Based Intervention (*Warna-Warni Waktu*) to Improve Body Image Among Young Indonesian Women: Parallel Randomized Controlled Trial"

Artikel Penelitian

Efikasi Intervensi Berbasis Media Sosial (*Warna-Warni Waktu*) dalam Meningkatkan Citra Tubuh Remaja Perempuan di Indonesia: Uji Acak Terkontrol Paralel

Penulis: Garbett KM, Haywood S, Craddock N, Gentili C, Nasution K, Saraswati LA, Medise BE, White, P, Diedrichs PC, & Williamson H

Korespondensi: Kirsty Garbett, Centre for Appearance Research, University of the West of England, Coldharbour Lane, Bristol, BS16 1QY, UK; +44 11732 86224 Kirsty.Garbett@uwe.ac.uk

Kontribusi penulis: Kirsty M Garbett: Konseptualisasi, Metodologi, Penulisan – Draf awal, Administrasi penelitian, Kurasi data, Pendanaan. Sharon Haywood: Penulisan – Draf awal, Administrasi penelitian, Kurasi data, Tenaga ahli. Nadia Craddock: Konseptualisasi, Metodologi, Penulisan – peninjauan & penyuntingan, Pendanaan. Caterina Gentili: Metodologi, Analisis formal, Penulisan – draf awal. Kholisah Nasution: Tenaga ahli, Administrasi penelitian, Penulisan – peninjauan & penyuntingan. L Ayu Saraswati: Tenaga ahli, Penulisan – peninjauan & penyuntingan. Bernie Endyarni Medise: Tenaga ahli, Penulisan – peninjauan & penyuntingan. Paul White: Metodologi, Analisis formal, Penulisan – peninjauan & penyuntingan. Phillippa C Diedrichs: Pengawasan, Konseptualisasi, Penulisan – peninjauan & penyuntingan, Pendanaan (Utama). Heidi Williamson: Pengawasan, Metodologi, Penulisan – peninjauan & penyuntingan.

Pendanaan: Studi ini mendapatkan hibah penelitian dari Dove Self-Esteem Project (Unilever). Sponsor tidak ikut serta dalam analisis data, pembuatan keputusan untuk memublikasikan, atau penyusunan naskah. Dove Self-Esteem Project (Unilever) diberikan kesempatan untuk meninjau naskah dan

menyarankan perubahan, tetapi keputusan akhir tentang isi naskah berada di tangan penulis sepenuhnya. Pandangan yang dikemukakan dalam artikel ini adalah pandangan penulis dan belum tentu sama dengan pandangan Unilever. Perlu diketahui bahwa Unilever tidak menyarankan perubahan apa pun dalam tulisan ini.

Abstrak

Latar Belakang: Ketidakpuasan terhadap tubuh *(body* dissatisfaction) merupakan masalah global, khususnya pada kelompok remaja perempuan. Meskipun ada, intervensi citra tubuh yang efektif sulit untuk disebarluaskan, khususnya di negara-negara berpenghasilan menengah dan rendah, seperti Indonesia, yang membutuhkan intervensi efektif tersebut.

Tujuan: Studi ini mengevaluasi akseptabilitas dan efikasi *Warna-Warni Waktu*, sebuah seri video yang terdiri dari enam episode cerita fiksi berbasis media sosial yang dilengkapi dengan kegiatan interaktif yang dapat dikerjakan sendiri (*self-guided*) dan didesain untuk meningkatkan citra tubuh remaja perempuan di Indonesia. Hipotesis penulis adalah *Warna-Warni Waktu* akan meningkatkan *trait body satisfaction* (kepuasan terhadap tubuh permanen) dan *trait mood* (suasana hati permanen) serta mengurangi internalisasi penampilan ideal dan ketidakpuasan terhadap warna kulit dibandingkan dengan kelompok kontrol. Selain itu, penulis memerkirakan *state body satisfaction* (kepuasan terhadap tubuh sementara) dan *state mood* (suasana hati sementara) akan meningkat sesaat setelah menonton masing-masing video.

Metode: Uji acak terkontrol dilakukan secara daring yang melibatkan dua kelompok (intervensi vs. kontrol) dengan peserta 2.000 orang remaja perempuan di Indonesia berusia 15 hingga 19 tahun yang direkrut melalui telepon oleh peneliti rekanan di Indonesia. Pengacakan blok dilakukan dengan alokasi 1:1. Pengacakan kelompok ini diketahui oleh peserta dan peneliti. Peserta melakukan penilaian diri tentang *trait body satisfaction* (luaran primer), internalisasi penampilan ideal, suasana hati *(mood)*, dan ketidakpuasan terhadap warna kulit (luaran sekunder) di awal (pra-pengacakan), 1 hari pasca-intervensi (T2), dan 1 bulan pasca-intervensi (T3). Peserta juga mengisi pengukuran *state body satisfaction* dan *state mood*, sesaat sebelum dan setelah menonton masing-masing video. Data dievaluasi dengan menggunakan

linear mixed model dengan metode *intent-to-treat analysis*. Adherensi terhadap intervensi juga dimonitor dan pertanyaan akseptabilitas diberikan pada T2.

Hasil: Sebanyak 1847 orang remaja perempuan di Indonesia ikut serta dalam penelitian ini. Dibandingkan dengan kelompok kontrol (n=923), kelompok intervensi (n=924) menunjukkan pengurangan internalisasi penampilan ideal pada T2 ($F_{1,\,1758}$=40,56, P<0,001, η2 parsial=0,022) dan T3 ($F_{1,\,1782}$=54,03, P<0,001, η2 parsial=0,03), serta pengurangan ketidakpuasan terhadap warna kulit pada T2 ($F_{1,\,1744}$=8,05, P<0,01, η2 parsial=0,005). Peningkatan *trait body satisfaction* terjadi di kelompok intervensi pada T3 ($F_{1,\,1781}$=9,02, P<0,01, η2 parsial=0,005). Temuan ini sepenuhnya dimediasi oleh skor perubahan internalisasi antara T1 dan T2 (efek tidak langsung: β=0,03, 95% CI 0,017-0,041; efek langsung: β=0,03, P=0,13), yang sesuai dengan *Tripartite Influence Model* untuk ketidakpuasan terhadap tubuh. Tidak terdapat temuan efek yang signifikan terhadap *trait mood*. Uji *t* sampel dependen menunjukkan bahwa masing-masing video efektif dalam meningkatkan *state body satisfaction* dan *state mood*. Analisis kumulatif menemukan bahwa terjadi peningkatan yang signifikan dan progresif pada skor *state body satisfaction* dan *state mood* antara sebelum dan setelah menonton video. Adherensi intervensi penelitian ini baik; peserta rata-rata menonton 5,2 video (SD=1,66). Skor akseptabilitas tinggi dicapai untuk kemudahan dipahami, kesenangan, kesesuaian dengan usia, manfaat, dan kemungkinan untuk merekomendasikan intervensi.

Kesimpulan: *Warna-Warni Waktu* adalah intervensi *eHealth* (layanan kesehatan secara elektronik) yang efektif dalam mengurangi ketidakpuasan terhadap tubuh remaja perempuan di Indonesia. Walaupun efeknya tidak besar, *Warna-Warni Waktu* adalah alternatif hemat biaya yang dapat disebarluaskan dibandingkan berbagai intervensi yang lebih intensif. Hal ini menjadikan *Warna-Warni Waktu* dapat dipertimbangkan sebagai salah satu pilihan di antara upaya-upaya intervensi yang ada saat ini, khususnya bagi negara yang belum menganggap ketidakpuasan terhadap tubuh merupakan masalah kesehatan.

Diseminasi intervensi *Warna-Warni Waktu* rencananya dilakukan melalui iklan media sosial berbayar agar dapat menjangkau ratusan ribu remaja perempuan di Indonesia.

Registrasi Penelitian: ClinicalTrials.gov NCT05383807; https://clinicaltrials.gov/ct2/show/NCT05383807; *International Registered Report Identifier* (IRRID) PRR1-10.2196/33596; ISRCTN35483207; https://www.isrctn.com/ISRCTN35483207

Kata Kunci: Citra Tubuh; Ketidakpuasan terhadap Tubuh; Indonesia; Kesehatan Mental Remaja; Uji Acak Terkontrol; Intervensi *eHealth*; Asia Tenggara; Media Sosial; LMIC

Pendahuluan

Banyak remaja di seluruh dunia, terutama remaja perempuan [1, 2], memiliki pikiran dan perasaan negatif tentang tubuhnya. Pikiran dan perasaan negatif ini disebut sebagai ketidakpuasan terhadap tubuh [3]. Studi mengungkapkan bahwa ketidakpuasan terhadap tubuh remaja perempuan ini di kemudian hari menimbulkan konsekuensi negatif terhadap kesehatan mental dan fisik, seperti gangguan perilaku makan, suasana hati yang buruk, dan rasa percaya diri yang rendah [4]. Oleh karena itu, diperlukan tindakan untuk mengurangi ketidakpuasan terhadap tubuh ini.

Berbagai intervensi telah dikembangkan dan dievaluasi secara ketat untuk mengurangi ketidakpuasan terhadap tubuh remaja perempuan dengan hasil yang menjanjikan [5-9]. Intervensi ini biasanya dilakukan secara tatap muka, dalam kelompok kecil, dan dipandu oleh profesional atau fasilitator yang telah mendapatkan pelatihan profesional. Pendekatan semacam ini membuat intervensi tidak dapat disebarluaskan karena mahal dan tenaga profesional kesehatan mental yang tidak banyak tersedia [10]. Hambatan lainnya dalam menghadiri intervensi tatap muka meliputi kurangnya anonimitas dan privasi ketika membahas isu-isu sensitif [11], serta hambatan logistik, seperti jarak geografis dan terbatasnya ketersediaan logistik yang diperlukan [12]. Hambatan ini lebih kuat terasa di negara-negara berpenghasilan menengah dan rendah (*Lower- and Middle-Income Country*/LMIC). Di negara-negara tersebut, layanan kesehatan yang tersedia bahkan lebih terbatas, stigma yang melekat pada kesehatan mental lebih kental, dan dalam konteks geografis yang beragam seperti di Indonesia, intervensi semacam ini hanya dapat diakses oleh segelintir orang [10, 13]. Sedangkan kebanyakan intervensi yang efektif mengurangi ketidakpuasan terhadap tubuh dikembangkan dan dievaluasi dalam konteks negara-negara berpenghasilan tinggi yang penduduknya berbahasa Inggris, terutama Australia, Amerika Serikat, dan Eropa Barat [14]. Hanya sedikit intervensi yang didesain secara khusus atau diadaptasi sesuai dengan budaya bukan negara Barat (misalnya, lihat [15-17]). Penelitian menunjukkan bahwa wilayah yang berbeda memiliki masalah penampilan yang berbeda pula [18-20]. Oleh karena itu, norma dan standar budaya perlu dipegang teguh dan disertakan dalam desain intervensi untuk memastikan efektivitas dan

akseptabilitas intervensi tersebut [21, 22]. Intervensi yang didesain secara khusus sesuai dengan budaya sangat penting untuk memerangi beban global ketidakpuasan terhadap tubuh remaja perempuan.

Solusi yang berpotensi meningkatkan cakupan intervensi kesehatan mental, termasuk intervensi untuk mengurangi ketidakpuasan terhadap tubuh adalah menggunakan *platform* media sosial [23, 24]. Terlepas dari risiko media sosial yang banyak terbukti bagi remaja [25-27], salah satu keunggulan utama *platform* ini adalah mudahnya informasi diakses oleh orang-orang yang membutuhkan [28]. Selama beberapa tahun terakhir, penggunaan media sosial melonjak drastis, dan hampir seluruh remaja di dunia menggunakannya [29]. Melalui intervensi berbasis media sosial, sebagian besar hambatan dalam mengakses pelayanan kesehatan mental dengan metode tradisional tatap muka dapat dihindari karena cara ini lebih hemat dan tidak bergantung pada tenaga profesional kesehatan mental, tidak membutuhkan kehadiran fisik, serta mengurangi stigma sosial berkat anonimitas yang ditawarkan situs jejaring sosial [30]. Selain itu, dengan intervensi berbasis media sosial, penentuan target intervensi dapat dilakukan melalui ruang digital yang mudah diakses dan dikunjungi setiap hari. Hal ini patut dipertimbangkan mengingat tingginya tingkat atrisi intervensi *eHealth* lainnya, seperti intervensi melalui aplikasi [31]. Sehubungan dengan ketidakpuasan tubuh, terdapat banyak penelitian yang mendokumentasikan dampak negatif dari melihat gambar penampilan yang dianggap ideal di media sosial [32]. Hanya sedikit penelitian yang mempelajari potensi penggunaan media sosial sebagai sarana diseminasi intervensi untuk mengurangi ketidakpuasan terhadap tubuh, sementara hasil penelitian penggunaan media sosial di bidang kesehatan mental secara umum menunjukkan hasil yang menjanjikan [33].

Terletak di Asia Tenggara, Indonesia adalah negara kepulauan terbesar dan memiliki penduduk terbanyak keempat di dunia [34]. Data menunjukkan bahwa ketidakpuasan terhadap tubuh dialami oleh setidaknya setengah dari remaja perempuan Indonesia [35] (KM Garbett, N Craddock, LA Saraswati, PC Diedrichs, data belum dipublikasikan, 2021), yang dikaitkan dengan gangguan perilaku makan pada kelompok umur ini [36]. Ketidakpuasan terhadap warna kulit dan keinginan untuk memiliki warna kulit yang lebih cerah juga terjadi [37, 38]. Hal ini membuat perempuan muda di Indonesia berisiko menggunakan produk pencerah kulit yang tidak aman [39] dan memiliki rasa percaya diri yang rendah

[40]. Remaja perempuan di Indonesia memiliki keinginan untuk belajar cara merasa lebih positif tentang penampilannya [41]. Namun, belum ada intervensi *eHealth* yang dikembangkan dan dievaluasi secara matang untuk kelompok populasi ini. Tingkat penetrasi internet di Indonesia tinggi [42], serta banyak remaja perempuan yang memiliki gawainya sendiri untuk mengakses akun media sosial [43]. Fakta ini beserta manfaat penggunaan internet yang telah disebutkan di atas dalam menyampaikan intervensi (yaitu, hemat biaya, mudah diakses, stigma sosial berkurang, hambatan fisik keikutsertaan teratasi) menunjukkan bahwa intervensi yang disampaikan melalui media sosial menjadi pilihan yang sangat berpotensi untuk mengurangi ketidakpuasan terhadap tubuh remaja perempuan di Indonesia.

Warna-Warni Waktu adalah intervensi berbasis media sosial yang dibuat khusus dan didesain untuk mengurangi ketidakpuasan terhadap tubuh remaja perempuan berusia 15-19 tahun di Indonesia. Intervensi ini terbentuk atas kerja sama peneliti dan Girl Effect, organisasi nirlaba internasional yang membuat konten media pemberdayaan bagi remaja perempuan; Dove Self-Esteem Project, kegiatan sosial yang dijalankan oleh Dove yang merupakan merek produk perawatan diri di bawah Unilever; Percolate Galactic, agensi kreatif di Indonesia yang berfokus pada pemasaran yang menargetkan kaum muda; dan para remaja perempuan Indonesia. Intervensi ini terdiri dari enam video pendek yang menceritakan karakter fiktif seorang perempuan muda bernama Putri serta kisahnya menghadapi tekanan di masyarakat terkait dengan penampilan selama masa remaja dan dewasa awal. Tiap video dilengkapi dengan sejumlah kegiatan interaktif singkat yang diformat untuk disebarkan di *platform* media sosial. Intervensi ini menargetkan faktor risiko sosial budaya pada ketidakpuasan terhadap tubuh yang diajukan *Tripartite Influence Model* (yaitu, media, teman, dan keluarga), yang dapat memperparah ketidakpuasan terhadap tubuh melalui proses internalisasi penampilan ideal dan perbandingan sosial [44]. Intervensi ini juga menerapkan beberapa teknik pengubah citra tubuh yang meliputi psikoedukasi, literasi media, dan disonansi kognitif, yang masing-masing telah terbukti efektif pada berbagai intervensi citra tubuh sebelumnya [5, 6, 14, 45]. Pengembangan intervensi ini dijelaskan secara rinci dalam makalah protokol studi [46] dan diringkas dalam artikel ini.

Pada studi ini, peneliti mengevaluasi dampak *Warna-Warni Waktu* dalam meningkatkan kepuasan terhadap tubuh dan luaran-luaran terkait remaja perempuan di Indonesia. Penulis melakukan uji efikasi acak paralel (1:1) untuk membandingkan keikutsertaan dalam *Warna-Warni Waktu* dengan kondisi kontrol. Cara ini dipilih karena luaran utama penelitian ini adalah dampak mutlak dari intervensi dan tidak adanya bantuan lain terhadap masalah ketidakpuasan terhadap tubuh di Indonesia [47]. Hipotesis penulis adalah: (1) peserta yang dipilih secara acak di kelompok intervensi akan mengalami peningkatan *trait body satisfaction* dan *trait mood*, dan penurunan internalisasi penampilan ideal dan ketidakpuasan terhadap warna kulit pada 1 hari pasca-intervensi dan 1 bulan setelahnya dibandingkan dengan kelompok kontrol; (2) tiap video akan langsung meningkatkan *state body satisfaction* dan *state mood*; dan (3) keterlibatan dan adherensi yang lebih tinggi dengan intervensi akan meningkatkan *trait body satisfaction* dan *state body satisfaction* serta *trait mood* dan *state mood*, dan menurunkan internalisasi penampilan ideal dan ketidakpuasan terhadap warna kulit.

Metode

Desain

Studi dilakukan dengan acak terkontrol paralel berbasis internet untuk menilai efikasi *Warna-Warni Waktu* pada kelompok intervensi dan kelompok kontrol (NCT05023213 dan ISRCTN35483207). Pengacakan blok dilakukan dengan alokasi 1:1 dan tiap blok terdiri dari 4, 6, dan 8 orang peserta. Surat keterangan kelaikan etik diperoleh dari Fakultas Kedokteran Universitas Indonesia (588/UN2.F1/ETIK/PPM.00.002/2021) dan University of the West of England, Bristol (Inggris) (HAS.21.04.138). Protokol studi juga telah dilakukan registrasi (*International Registered Report Identifier* PRR1-10.2196/33596) [46].

Peserta

Perekrutan dan pendaftaran remaja perempuan dalam penelitian ini dilakukan oleh peneliti rekanan di Jakarta secara luring di sepuluh kota di Indonesia bagian barat, tengah, dan timur (Balikpapan, Bandung, Jakarta, Makassar, Manado, Medan, Palembang, Pontianak, Semarang, dan Surabaya), dengan

komposisi usia dan status sosial ekonomi yang terbagi rata. Remaja perempuan yang direkrut dapat ikut serta jika memenuhi kriteria inklusi sebagai berikut: (1) berusia antara 15 dan 19 tahun, (2) memiliki telepon genggam sendiri, dan (3) mengunjungi Facebook atau Instagram setiap hari. Adapun kriteria eksklusi penelitian ini adalah jika remaja perempuan tersebut (1) mengikuti *(follow)* Girl Effect (Springster) di media sosial, (2) pernah mengakses situs web Springster sebelumnya, atau (3) tidak mendapatkan izin tertulis dari orang tua atau wali (jika berusia di bawah 18 tahun).

Perekrutan peserta dilakukan melalui telepon dengan menggunakan basis data peserta dewasa berusia di atas 40 tahun yang telah dimiliki sebelumnya. Peserta dewasa yang memiliki anak perempuan berusia antara 15 dan 17 tahun dibacakan lembar informasi orangtua. Jika orang tua memiliki lebih dari satu anak perempuan yang memenuhi syarat, hanya dipilih satu anak yang usianya memenuhi kuota usia ; jika lebih dari satu anak perempuan memenuhi kebutuhan kuota usia, dipilih anak perempuan yang ulang tahunnya paling dekat dengan tanggal panggilan telepon. Orangtua yang memberikan izin lisan selanjutnya memberikan informasi tentang status sosial ekonominya. Anak perempuan tersebut kemudian dinilai apakah memenuhi kriteria inklusi dan eksklusi. Jika anak memberikan persetujuan lisan untuk ikut serta, orangtua diminta persetujuannya secara tertulis melalui WhatsApp. Proses serupa dijalankan untuk anak perempuan berusia 18 atau 19 tahun yang memenuhi syarat dan mereka memberikan persetujuan lisan dan tertulis untuk dirinya sendiri. Identitas orangtua dan anak yang memenuhi kriteria kemudian dikonfirmasi melalui panggilan video berdasarkan tanda pengenal resmi yang mencantumkan foto.

Intervensi

Karena proses pembuatan dan isi dari *Warna-Warni Waktu* telah dijelaskan secara rinci dalam makalah protokol [46], penulis hanya menuliskan ringkasan singkat dalam artikel ini. Intervensi ini dibuat khusus, telah diuji, dapat digunakan tanpa bantuan orang lain, dan terdiri dari enam video pendek berurutan yang masing-masing berdurasi sekitar lima menit (Versi 1). Cerita dalam video berfokus pada seorang remaja perempuan, asli Indonesia yang sedang mengalami masalah citra tubuh yang umum dirasakan oleh sebayanya. Ia menemukan strategi untuk mengatasi tekanan yang terkait dengan

penampilan *(appearance-related pressure),* misalnya, produk pencerah kulit yang ditawarkan di media sosial, perbandingan penampilan, dan komentar dari teman, dengan bantuan dari karakter animasi penjelajah waktu. Video intervensi menargetkan faktor risiko utama yang diketahui berkontribusi pada timbulnya ketidakpuasan terhadap tubuh, yaitu media sosial dan *influencer* (video 2), perbandingan penampilan*/appearance-based comparison* (video 3), ejekan mengenai penampilan*/appearance-based teasing* (video 4), dan obrolan mengenai tubuh/*body talk* (video 5). Video 1 dan 6 tidak membahas faktor risiko tertentu tetapi memperkenalkan dan menyimpulkan keseluruhan cerita dalam *Warna-Warni Waktu*. Selain itu, tiap video dilengkapi dengan kegiatan daring yang didesain untuk memperkuat pesan utama video. Sebagian besar kegiatan dapat diselesaikan dalam waktu singkat, seperti melengkapi komik atau menemukan perubahan pada foto yang diedit. Di video 2-5 juga terdapat satu kegiatan untuk tiap video yang meminta peserta menulis jawaban hingga 250 kata untuk pertanyaan tentang pesan utama video. Kegiatan ini bernama “Dengan Kata-Katamu Sendiri” (dalam bahasa Inggris “*Your Own Words*”). Sebagai insentif atas keterlibatan dalam kegiatan yang membutuhkan waktu lebih panjang ini, peserta dengan jawaban terbaik di tiap kegiatan “Dengan Kata-Katamu Sendiri” diberikan pulsa senilai Rp50.000 (sekitar US$3,50). Cara ini telah terbukti efektif digunakan Girl Effect untuk remaja seperti pada penelitian ini. Jawaban peserta kemudian dinilai oleh penulis kelima (KN). Insentif ini juga akan diberikan saat penelitian utama berjalan.

Warna-Warni Waktu dikembangkan berdasarkan *Tripartite Influence Model* [44] yang menargetkan tiga faktor sosial budaya (yaitu, teman, keluarga, dan media) yang memengaruhi proses psikologis internalisasi penampilan ideal dan perbandingan sosial berdasarkan penampilan *(appearance-based social comparisons)*. Video juga membahas proses psikologis ini secara langsung bahwa diperlukan literasi media dan perbandingan berdasarkan penampilan dapat menyebabkan ketidakpuasan terhadap tubuh. Selain itu, teknik yang efektif mengurangi ketidakpuasan terhadap tubuh, yaitu disonansi kognitif dan psikoedukasi juga digunakan pada kegiatan tambahan setelah video [5].

Video dan kegiatan tambahan didesain untuk disebarluaskan melalui Facebook dan Instagram secara bertahap (*sequential marketing*) yang menargetkan remaja perempuan di Indonesia. Video juga

diunggah dan dapat diunduh gratis di YouTube. Namun, untuk keperluan penelitian, intervensi dibuat ulang sesuai dengan *platform* survei Qualtrics (Qualtrics International Inc) agar mendapatkan data adherensi di tingkat individu. Setiap video dan kegiatan terkait dituangkan ke dalam enam kuesioner Qualtrics yang berbeda; peserta diharapkan untuk menonton satu video dan menyelesaikan kegiatan terkait per satu hari (dalam waktu 24 jam) sesuai dengan urutan video diterima.

Prosedur

Semua komunikasi antara peneliti dan peserta dilakukan secara daring melalui WhatsApp oleh peneliti rekanan. Satu hari sebelum studi dimulai (Hari 0), paket data dibagikan kepada peserta untuk mengganti biaya data internet yang dikeluarkan. Peserta mengisi kuesioner laporan diri secara daring di Qualtrics pada tiga titik waktu: di awal (Hari 1), 1 hari setelah intervensi (Hari 9), dan 1 bulan setelah intervensi (Hari 36). Pertanyaan dalam kuesioner tertutup tidak diberikan secara acak. Pertanyaan adaptif hanya digunakan untuk menanyakan izin di awal tiap kuesioner. Kuesioner rata-rata berisi 31 tampilan layar, dengan 4-5 pertanyaan per laman. Tombol “Kembali” disertakan pada layar sehingga peserta memiliki pilihan untuk memeriksa dan/atau mengubah jawabannya. Fungsionalitas kuesioner telah diuji dan diverifikasi oleh penulis (KG, SH) sebelum tautan Qualtrics dibagikan.

Pada pukul 08.00 WIB (UTC +7) di hari 1 (Waktu 1/T1), hari 9 (Waktu 2/T2), dan hari 36 (Waktu 3/T3), peserta akan mendapatkan tautan kuesioner dan nomor unik identifikasi peserta (PIN) yang harus peserta masukkan di awal tiap kuesioner. Peserta diberikan waktu 24 jam untuk mengisi lembar penilaian diri sendiri. Pesan pengingat dikirimkan kepada peserta yang belum mengisi kuesioner dalam waktu 8 jam pertama (pesan ini tidak akan ada ketika intervensi di media sosial). Informasi demografis dikumpulkan melalui kuesioner awal (*baseline*).

Setelah peserta melengkapi kuesioner awal, peneliti yang tidak terlibat dalam proyek ini dan tidak mengetahui informasi serta kondisi peserta menentukan urutan alokasi (berdasarkan desain blok) dengan menggunakan pengacak otomatis berbasis web [48] yang membagi peserta ke dalam kelompok intervensi atau kelompok kontrol *(waitlist control group)*. Alokasi ini diberitahukan kepada peserta melalui

WhatsApp satu hari setelah peserta menyelesaikan kuesioner dasar. Peserta di kelompok intervensi diberitahukan tentang waktu pengiriman video intervensi dan peserta di kelompok kontrol diberitahukan bahwa tautan asesmen laporan diri kedua akan dikirimkan dalam waktu satu minggu. Peneliti rekanan mengetahui tentang pembagian acak kelompok peserta ini.

Peserta yang terbagi acak ke kelompok intervensi dikirimkan PIN dan tautan Qualtrics dengan satu video dan kegiatan terkait di Hari 3-8 pada pukul 8.00 WIB (UTC +7). Tiap tautan menyertakan pengukuran *state body satisfaction* dan *state mood* yang harus diisi sesaat sebelum dan setelah menonton masing-masing video intervensi. Peserta diberikan waktu 24 jam untuk mengerjakan tiap tautan (yaitu, menonton video dan menyelesaikan kegiatan daring terkait), dan pesan pengingat dikirimkan kepada peserta yang belum mulai mengerjakan intervensi setelah 8 jam. Informasi akseptabilitas diperoleh dari peserta kelompok intervensi pada akhir kuesioner Hari 9.

Satu hari setelah kuesioner terakhir (Hari 37), semua peserta diberikan sertifikat keikutsertaan dan dokumen tanya jawab yang menjelaskan tujuan studi, menyertakan kontak dukungan kesehatan mental, dan menyediakan tautan akses ke seri video intervensi. Sesaat setelahnya, insentif senilai Rp125.000 (sekitar US$8,75) dihadiahkan kepada para peserta yang berhasil menyelesaikan ketiga kuesioner. Hadiah bagi para pemenang kegiatan “Dengan Kata-Katamu Sendiri” dikirimkan dua minggu setelah penelitian selesai. Sebagaimana dijelaskan dalam protokol penelitian [46], proses yang disebutkan di atas (kecuali pesan pengingat yang dikirimkan kepada peserta intervensi pada Hari 3-8; lihat bagian Hasil Studi Percontohan) telah dicoba sebelumnya secara internal dengan melibatkan 150 orang peserta untuk mengidentifikasi perubahan prosedur yang diperlukan pada penelitian utama.

Pengukuran

Pengukuran Luaran Primer

Pengukuran luaran primer untuk menilai *trait body satisfaction* menggunakan kuesioner *Body Esteem Scale for Adolescents and Adults* (BESAA) [49] yang telah divalidasi secara daring pada remaja di Indonesia (KM Garbett, N Craddock, S Haywood, C Hayes, K Nasution, LA Saraswati, B Medise, S

Vitoratou, PC Diedrichs, data tidak dipublikasikan, 2022). Tiga subskala (Penampilan Positif, Penampilan Negatif, dan Berat Badan) diterapkan dan dikombinasikan untuk menghasilkan skor skala total. Skor yang semakin tinggi menunjukkan *trait body satisfaction* yang semakin besar. Konsistensi internal pada kondisi dasar baik; Cronbach α=0,80.

Pengukuran Luaran Sekunder

Luaran sekunder diukur dengan menggunakan tiga metode pengukuran. Pertama, pengukuran menggunakan kuesioner subskala *Internalization-General* dari skala *Sociocultural Attitudes Towards Appearance Questionnaire* (SATAQ-3) [50] yang telah divalidasi secara daring pada remaja di Indonesia (KM Garbett, N Craddock, S Haywood, C Hayes, K Nasution, LA Saraswati, B Medise, S Vitoratou, PC Diedrichs, data belum dipublikasikan, 2022). Skor yang semakin tinggi menunjukkan internalisasi penampilan ideal yang semakin besar. Konsistensi internal kuesioner ini baik; Cronbach α=0,95. Kedua, penilaian *trait mood* menggunakan kuesioner *Positive and Negative Affect Schedule for Children* (PANAS-C) [51] yang telah divalidasi secara daring pada remaja di Indonesia (S Haywood, KM Garbett, N Craddock, C Hayes, LA Saraswati, K Nasution, B Medise, S Vitoratou, PC Diedrichs, data belum dipublikasikan, 2022). Kuesioner versi Indonesia yang telah divalidasi terdiri dari dua subskala yang mirip dengan versi asli PANAS-C, yaitu subskala emosi positif (skor yang semakin tinggi menunjukkan suasana hati positif yang semakin besar) dan subskala emosi negatif (skor yang semakin tinggi menunjukkan suasana hati negatif yang semakin besar). Konsistensi internal kuesioner ini baik; Cronbach α=0,90 untuk emosi positif dan Cronbach α=0,88 untuk emosi negatif. Ketiga, pengukuran khusus untuk menilai ketidakpuasan terhadap warna kulit dibuat untuk penelitian ini karena tidak tersedianya pengukuran yang telah divalidasi. Dengan menggunakan bagan yang menggambarkan sembilan warna kulit, dari gelap (1) hingga cerah (9) pada *Pantone Skin Tone Guide* [52], peserta memilih warna yang paling mencerminkan 'warna kulitnya saat ini' dan 'warna kulit idealnya'. Skor ketidakpuasan terhadap warna kulit diperoleh dari selisih antara dua deskripsi tersebut dan berkisar dari 0 (*puas dengan warna kulit*) hingga 8 (*sangat tidak puas dengan warna kulit*).

Pengukuran Luaran Perasaan Sementara (State)

Untuk menilai *state body satisfaction* dan *state mood*, pengukuran dengan *single-item measure* pada skala analog visual 101 poin [53] digunakan sesaat sebelum dan setelah masing-masing video intervensi. Skor yang semakin tinggi menunjukkan masing-masing kepuasan terhadap tubuh yang semakin besar dan suasana hati yang semakin baik.

Akseptabilitas Intervensi

Pada Hari 9, peserta di kelompok intervensi diberikan enam pertanyaan penilaian diri di akhir kuesioner T2 untuk menilai apakah video intervensi dinikmati oleh peserta secara keseluruhan, kesukaan terhadap karakter, kemudahan dipahami, kesesuaian dengan usia, manfaat, dan kemungkinan intervensi direkomendasikan kepada teman sebaya. Pilihan jawaban berkisar dari 1 (*sangat tidak setuju*) hingga 5 (*sangat setuju*).

Adherensi

Tingkat adherensi terhadap intervensi dinilai melalui berbagai parameter yang melihat keterlibatan dalam video dan kegiatan. Parameter tersebut, yaitu persentase peserta yang menonton tiap video dan keenam video (dihitung dari jumlah peserta yang durasi kunjungannya ke video di laman Qualtrics sama panjang atau lebih panjang dari durasi video); jumlah rerata video yang ditonton; persentase peserta yang menyelesaikan tiap kegiatan; jumlah rerata kegiatan yang diselesaikan; dan jumlah rerata durasi peserta terlibat dengan keseluruhan intervensi (yaitu, keenam video dan kegiatan terkait).

Jumlah Sampel

Beberapa penelitian acak terkontrol serupa yang menilai ketidakpuasan terhadap tubuh dengan menggunakan pengukuran luaran yang sama melaporkan besaran efek (*effect size*) terstandardisasi dari kecil hingga sedang, dengan nilai Hedge's *g* mulai dari 0,25 hingga 0,4 yang melebihi *Minimum*

Important Clinical Difference (MICD) [misalnya, 54]. Untuk mendapatkan MICD atau nilai yang lebih besar, jumlah sampel N=900 per kelompok yang diajukan oleh penulis akan menghasilkan kekuatan (*power*) lebih dari 90% (2 sisi, α=0,05) untuk perbedaan antar kelompok pada T2 atau T3. Hal ini dengan asumsi angka *dropout* tidak lebih dari 20% di satu kelompok.

Analisis

Analisis dilakukan dengan menggunakan SPSS 28 (IBM Corp). PIN unik peserta digunakan untuk mengidentifikasi duplikasi entri kuesioner. Jika terdapat duplikasi, entri pertama disimpan untuk keperluan analisis. Guna menghindari bias dalam interpretasi, hal-hal saat alokasi dirahasiakan dari analis data selama penyusunan data dan pengujian hipotesis luaran *trait*. Namun, hal-hal saat alokasi tersebut tidak mungkin dirahasiakan selama uji hipotesis luaran *state* dikarenakan desain *within-group* yang diterapkan dalam studi ini. Data pengukuran luaran *state* baru dibagikan kepada analis data ketika analisis pengukuran luaran *trait* telah selesai.

Pengujian Hipotesis & Analisis Post-hoc

Luaran *Trait*

Efek intervensi pada luaran *trait* diperiksa dengan menjalankan empat *Linear Mixed Model* (LMM) secara *intention-to-treat* (ITT) dengan pengukuran dasar di T1 sebagai kovariat; kelompok acak *(randomized group)* sebagai faktor dua tingkat antar subjek; fase studi*/study phase* (T2, T3) sebagai faktor pengukuran berulang dua tingkat (matriks kovarians tak berstruktur); dan metode estimasi *Restricted Maximum Likelihood*. Model statistik ini seimbang secara hierarki, dengan satu *three-way interactions* antara kovariat, fase studi, dan *kelompok acak*; tiga *two-way interactions* (kovariat*fase studi; kovariat*kelompok acak; fase studi*kelompok acak); dan tiga efek utama (kovariat, *phase*, dan *kelompok acak*). Untuk estimasi ukuran efek, peneliti menghitung *partial eta squared* untuk setiap model faktor.

Untuk memperkirakan apakah kelompok acak berkontribusi secara signifikan terhadap LMM, dijalankan empat tes *–2 Log Likelihood* (–2LL) dengan membandingkan LMM lengkap yang telah dijelaskan di atas dengan LMM tanpa kelompok acak dan interaksinya. Model ini mencakup pengukuran dasar di T1 sebagai kovariat, fase studi (T2, T3) sebagai faktor pengukuran berulang dua tingkat, dan satu *two-way interaction* (kovariat*fase studi). Untuk uji –2LL, kedua model dijalankan dengan matriks kovarian tidak berstruktur dan metode estimasi *Maximum Likelihood.*

Untuk setiap hasil *trait*, dua ANCOVA *pre-planned* dijalankan untuk memverifikasi efek *kelompok acak* di T2 dan T3 secara terpisah. Model tersebut mencakup pengukuran dasar sebagai kovariat, *kelompok acak* sebagai faktor tetap, dan pengukuran pada T2 atau T3 sebagai variabel-variabel dependen. Kesimpulan-kesimpulan penelitian utama diambil dari ANCOVA *pre-planned* ini.

Peneliti juga menjalankan dua ANOVA pengukuran berulang post-hoc untuk setiap hasil *trait*, masing-masing satu untuk kontrol dan satu untuk kelompok intervensi, untuk menguji tren waktu pada ketiga titik waktu.

Terakhir, efek dosis-respons *pre-planned* diuji dalam kondisi intervensi melalui analisis regresi multipel dengan masing-masing hasil *trait* di T2 dan T3 sebagai variabel dependen, dan skor keterlibatan berkode Helmert (misalnya: jumlah video yang ditonton) sebagai variabel independen. Pengkodean Helmert memungkinkan perbandingan setiap tingkat keterlibatan terhadap tingkat yang lebih tinggi yang tersisa, sehingga memungkinkan identifikasi titik lompatan/*jump points* potensial dalam data [55].

Luaran *State*

Untuk masing-masing luaran *state*, enam sampel *t* dependen diteliti untuk membandingkan tingkat kepuasan tubuh dan *mood* segera sebelum dan sesudah menonton setiap dari enam video intervensi.

Untuk menguji efek kumulatif di enam video, kami melakukan dua 2x6 [(pra-video vs pasca-video) x (enam video)] pengukuran berulang penuh ANOVA untuk pernyataaan kepuasan tubuh dan *state mood,* memeriksa tren linear, kuadrat, dan kubik, serta kontras berulang. Untuk kedua ukuran *state*, *gain score* untuk setiap video dihitung (skor status pasca-video dikurangi skor status pra-video) dan kemudian uji

sampel *t* dependen post-hoc diujikan pada *gain score* yang berdekatan. Terakhir, dilakukan dua pengukuran berulang post-hoc ANOVA untuk setiap hasil *state*: satu untuk skor pra-video dan satu untuk skor pasca-video masing-masing untuk memeriksa tren waktu dalam skor pra-dan pasca-*state* secara terpisah.

Analisis Eksploratif

Setelah menjalankan pengujian hipotesis dan analisis post-hoc, peneliti menguji model mediasi eksploratif dengan PROCESS Macro untuk SPSS (Model 4) [56]. Kami menghitung *gain score* internalisasi antara T1 dan T2 dengan mengurangkan skor internalisasi pasca-tes dengan skor internalisasi pra-tes (skor yang lebih tinggi menunjukkan penurunan internalisasi yang lebih besar dari pra- ke pasca-intervensi). Pada model ini, kelompok acak dimasukkan sebagai variabel independen, perubahan internalisasi dari pra-tes ke pasca-tes sebagai mediator, *trait body dissatisfaction* pada tindak lanjut sebagai variabel dependen, dan *trait body dissatisfaction* pada pra-tes sebagai kovariat.

Persiapan Data

Data hasil *trait* yang ditetapkan di seluruh kelompok memiliki 0,33% (356/112667) butir respons tidak lengkap di T1; 4,61% (4858/112667) butir respons tidak lengkap di T2; dan 3,34% (3517/112667) butir respons tidak lengkap di T3 (Lampiran Multimedia 2).[1]

Uji sampel t independen memastikan data yang tidak lengkap untuk pengukuran luaran primer (BESAA) pada T2 dan T3 tidak tergantung pada nilai dasar *trait body dissatisfaction* (t_{1845}= 0,93, *P(two-sided)* =0,35 pada T2; t_{1845}= 1,0, *P*=0,32 apada T3). Analisis Chi-*square* menunjukkan bahwa data yang tidak lengkap pada T2 ataupun T3 tidak secara signifikan berbeda antar kelompok yang diacak (χ^2_1: = 3,48, *P(two-sided)* =0,06). Analisis menggunakan uji *MCAR Little* menunjukkan bahwa data yang tidak lengkap konsisten dengan data tidak lengkap secara acak baik antara T1 dan T2 (χ^2_1: = 0,87, *P(two-sided)* =0,35) dan antara T1 dan T3 (χ^2_1: = 1,0, *P(two-sided)*=0,31).

---

[1] Rumus untuk % butir yang hilang: [N tanggapan butir yang hilang untuk setiap skala pada setiap titik waktu/(butir skala pengukuran*N peserta)]*100.

Selain itu, tingkat *dropout* (peserta yang tidak menyelesaikan survei) tidak pernah melebihi 5% antar titik waktu untuk variabel mana pun, sehingga mendukung kesimpulan bahwa data yang hilang tidak perlu dikhawatirkan untuk kumpulan data ini mengingat tingkat retensi yang tinggi [57]. Peserta yang gagal menyelesaikan setidaknya 80% butir pada skala apapun dikeluarkan dari analisis untuk skala tersebut. Tingkat atrisi antar titik waktu rendah, dengan besar total maksimum respons hilang 5,77% ((918/924) - (871/924)) antara T1 dan T2 pada ketidakpuasan *skin shade* pada kelompok intervensi. Beberapa peserta dengan respons tidak lengkap di T2 memberikan respons lengkap di T3. Dibandingkan dengan T1, pada tindak lanjut kami mendapati respons tidak lengkap maksimum 3,35% ((924/924) - (893/924)) pada *trait body dissatisfaction* di kelompok intervensi (Lampiran Multimedia).[2]

Untuk pengujian hipotesis, LMM dan ANCOVA post-hoc dilakukan berdasarkan ITT, tanpa imputasi data. Karena persentase tidak lengkap *(missing)* di bawah 5%, LMM dan ANCOVA dianggap memadai untuk kumpulan data yang tidak lengkap [58]. Imputasi data dan analisis pra-protokol tidak dilakukan untuk menghindari bias dalam distribusi variabel hasil dengan persentase minimal dari data yang tidak lengkap (mis., [59]).

Asumsi LMM dan ANCOVA akan linearitas dari residu, variabel dependen kontinu, *homogeneity of regressions slopes*, *homogeneity of covariance matrixes*, dan tidak adanya kolinearitas terpenuhi semuanya untuk luaran *trait*. Asumsi homoskedastisitas terpenuhi untuk semua hasil *trait* dengan hanya sedikit pelanggaran untuk skor internalisasi. Meskipun tidak mencapai normalitas sempurna, semua residu dari hasil *trait* pada T2 dan T3 hanya menunjukkan kecondongan/*skewness* minimal (-2<*skewness*<+2) dan kurtosis minimal (kurtosis ekses<5). Data tidak diubah, maupun skor *outlier* diganti, mengingat ANCOVA sangat tegas terhadap pelanggaran distribusi normal residu ketika dijalankan untuk ukuran sampel yang memadai dan kelompok-kelompok berukuran sama [60].

---

[2] Persentase mewakili pengurangan antarpecahan. Pecahan pertama mengacu pada jumlah peserta yang menyelesaikan kuesioner di T1 dibagi dengan jumlah peserta yang diacak dalam kondisi tersebut. Pecahan kedua mengacu pada total peserta yang menyelesaikan kuesioner di T2 atau T3, dibagi dengan total peserta yang diacak dalam kondisi tersebut.

Tingkat atrisi juga rendah untuk hasil *state*. Untuk kelompok intervensi, respon tidak lengkap *(missing response)* pada pengukuran *state* sebanyak satu butir berkisar antara 12,13% (112/924) (pra-video 1) dan 13,85% (128/924) (pasca-video 6) untuk kepuasan tubuh dan antara 10,86 % (111/924) (pra-video 1) dan 13,97% (129/924) (pasca-video 6) untuk suasana hati (Lampiran Multimedia 4).

Hasil

Studi Pilot dan Perubahan Protokol

Rekrutmen untuk studi pilot dilakukan dari 13 sampai 16 September 2021, dan dilaksanakan antara 18 dan 26 September 2021. Sampel yang tersebar merata di seluruh usia dan status sosial ekonomi (N=150) diperoleh dari kota Jakarta, Medan, dan Semarang. Sebagaimana telah diuraikan dalam protokol penelitian [46], peneliti menggunakan kriteria berikut sebagai panduan memutuskan untuk melanjutkan ke penelitian utama: retensi peserta di T1 dan T2, kepatuhan intervensi (yaitu, melihat semua enam video), kualitas data (yaitu, penyelesaian akurat tiap ceklis survei), dan pengkajian bahaya (yaitu, perubahan *trait body dissatisfaction* pada peserta intervensi antara T1 dan T2 dibandingkan dengan peserta kontrol). Dengan menggunakan sistem sesuai lampu lalu lintas, kriteria tersebut dikaji dan dikategorikan sebagai hijau (lanjutkan uji coba utama), kuning (konsultasikan dengan tim peneliti dan buat perubahan), atau merah (pertimbangkan kembali protokol atau pertimbangkan penghentian penelitian). Tiga kriteria di atas diklasifikasikan sebagai hijau, yaitu retensi peserta adalah 98,67% (N=148/150); kualitas data dikategorikan kuat karena 97,3% peserta menjawab dengan benar semua pemeriksaan perhatian di T1 dan T2; dan tidak ada indikasi bahaya yang terlihat di setiap pengukuran luaran, dibandingkan dengan kelompok kontrol. Kepatuhan penuh terhadap intervensi adalah 67,12% (n=49/73), yang dikategorikan sebagai kuning. Untuk meningkatkan kepatuhan intervensi dalam penelitian utama, pesan pengingat diberikan pada Hari 3-8 kepada mereka yang belum menyelesaikan intervensi setelah 8 jam sejak menerima tautan intervensi. Data pilot dimasukkan ke dalam analisis penelitian utama karena tidak ada perubahan signifikan yang dilakukan terhadap desain atau intervensi

studi. Proses ini dilakukan untuk memastikan dana penelitian digunakan dengan hemat dan efisien [61].

Penelitian Utama

Rekrutmen berlangsung antara 12 Oktober dan 5 November 2021. Penelitian utama dilaksanakan dari 6 November sampai 12 Desember 2021. Diagram alur peserta studi utama digambarkan pada Gambar 1. Peserta berusia 15-19 tahun (rerata intervensi 16,94, SD 1,40 tahun; rerata kontrol 17, SD 1,41 tahun). Tidak ada perbedaan yang signifikan antarkelompok (t_{1845}=0,87, P(2-sided)=0,38) dan sampel dikelompokkan sama rata berdasarkan usia. Kedua kelompok serupa untuk semua karakteristik demografis lainnya. Sebagian besar peserta lahir di Indonesia. Mereka direkrut dari sepuluh kota di Indonesia dengan sebagian besar peserta tinggal di Jakarta (Tabel 1). Etnis yang paling umum adalah Jawa diikuti oleh Sunda, yang jika digabungkan menyumbang setengah dari total sampel; sembilan etnis lainnya di Indonesia menyumbang seperlima sampel (Tabel 1 dan Lampiran Multimedia 5). Dua pertiga peserta berasal dari kelompok sosioekonomi menengah, diikuti oleh lebih dari seperempat peserta dari kelompok sosioekonomi atas (Tabel 1). Sebagian besar peserta mengidentifikasi diri sebagai Muslim (agama Islam) (Tabel 1). Kedua kelompok tidak berbeda variabel luaran *trait* pada kuesioner awal (Tabel 2). Rerata waktu penyelesaian berkisar antara 19 dan 39 menit, dengan tidak ada perbedaan signifikan antarkelompok pada titik waktu manapun (Lampiran Multimedia 6). Oleh karena itu, kita dapat menyimpulkan bahwa pengacakan berhasil.

Gambar 1. Bagan alur peserta.

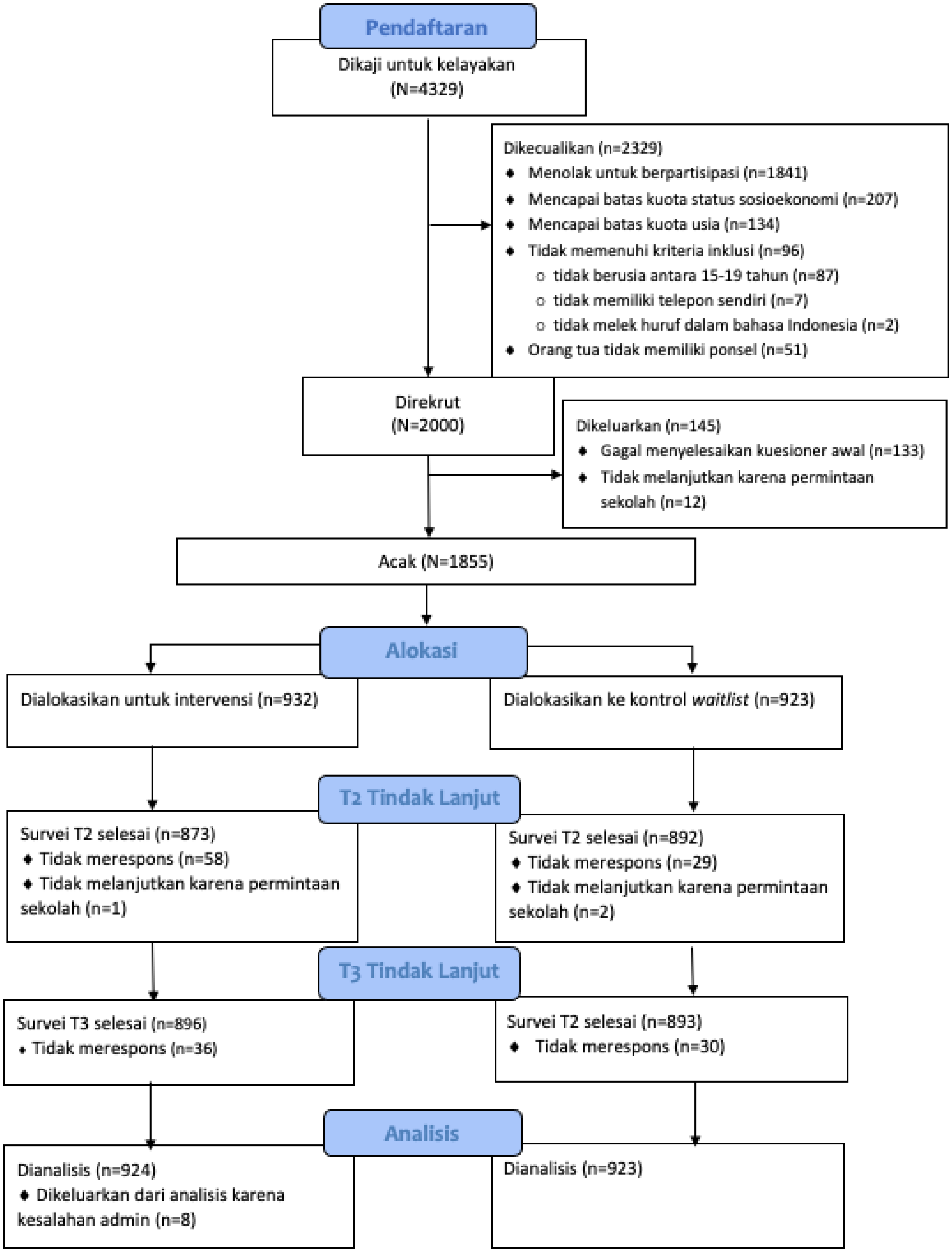

Tabel 1. Data demografi awal peserta.

	Total (N = 1847)	Kontrol (N = 923)	Intervensi (N = 924)
Umur tahun, rerata (SD)	16,96 (1,38)	17,0 (1,41)	16,94 (1,40)
**Usia (tahun), n (%)**			
15	371 (20,1)	183 (19,8)	188 (20,3)
16	363 (19,7)	174 (18,9)	189 (20,5)
17	411 (22,3)	197 (21,3)	214 (23,2)
18	376 (20,4)	201 (21,8)	175 (18,9)
19	326 (17,7)	168 (18,2)	158 (17,1)
**Negara kelahiran, n (%)**			
Indonesia	1846 (99,9)	922 (99,9)	924 (100,0)
Malaysia	1 (0,1)	1 (0,1)	0 (0)
**Kota, n (%)**			
Balikpapan	98 (5,3)	45 (4,9)	53 (5,7)
Bandung	259 (14,0)	140 (15,2)	119 (12,9)
Jakarta Raya	401 (21,7)	190 (20,6)	211 (22,8)
Makassar	102 (5,5)	48 (5,2)	54 (5,8)
Manado	94 (5,1)	48 (5,2)	46 (5,0)
Medan	176 (9,5)	90 (9,8)	86 (9,3)
Palembang	144 (7,8)	75 (8,1)	69 (7,5)
Pontianak	97 (5,3)	49 (5,3)	48 (5,2)
Semarang	219 (11,9)	109 (11,8)	110 (11,9)
Surabaya	257 (13,9)	129 (14,0)	128 (13,9)
**Etnisitas, n (%)**			
Batak	74 (4,0)	28 (3,0)	46 (5,0)
Betawi	84 (4,6)	46 (5,0)	38 (4,1)

Bugis	39 (2,1)	19 (2,1)	20 (2,2)
Jawa	665 (36,0)	344 (37,3)	321 (34,7)
Makassar	47 (2,5)	23 (2,5)	24 (2,6)
Melayu	53 (2,9)	26 (2,8)	27 (2,9)
Minang	34 (1,8)	17 (1,8)	17 (1,8)
Palembang	68 (3,7)	30 (3,3)	38 (4,1)
Sunda	255 (13,8)	123 (13,3)	132 (14,3)
Etnis/suku lain[a]	157 (8,5)	79 (8,6)	78 (8,4)
Tidak tahu	140 (7,6)	79 (8,6)	61 (6,6)
Jawaban salah (mis. kota tempat tinggal)	148 (8,0)	65 (7,0)	83 (9,0)
Tidak menanggapi	83 (4,5)	44 (4,8)	39 (4,3)
**Agama, n (%)**			
Kristen, Advent	2 (0,1)	1 (0,1)	1 (0,1)
Kristen, Katolik	20 (1,1)	15 (1,6)	5 (0,5)
Kristen, Protestan	126 (6,8)	69 (7,5)	57 (6,2)
Konfusianisme	2 (0,1)	1 (0,1)	1 (0,1)
Hindu	1 (0,05)	1 (0,1)	0 (0)
Islam	1691 (91,9)	834 (90,4)	857 (92,7)
Memilih untuk tidak menjawab	5 (0,3)	2 (0,2)	3 (0,3)
**Status sosioekonomi, n (%)**			
Bawah I	167 (9,0)	87 (9,4)	80 (8,7)
Bawah II	3 (0,2)	3 (0,3)	0 (0)
Tengah I	854 (46,2)	408 (44,2)	446 (48,3)
Tengah II	312 (16,9)	163 (17,7)	149 (16,1)
Atas I	158 (8,6)	79 (8,6)	79 (8,5)

Atas II	353 (19,1)	183 (19,8)	170 (18,4)

[a] Lihat Lampiran Multimedia 5 untuk laporan lengkap etnisitas.

Tabel 2. Nilai luaran *trait* untuk kedua kelompok pada setiap titik waktu.

Pengukuran luaran *trait*	T1[a]				T2[b]		T3[c]	
	Kontrol	Int.[d]	uji *t* (*df*)[e]	*Nilai P*	Kontrol	Int.[d]	Kontrol	Int.[d]
**Pengukuran luaran primer, rerata (SD)**								
BESAA[f] (kisaran 1-5)	3,46 (0,63)	3,45 (0,61)	0,29 (1845)	0,77	3,46 (0,63)	3,43 (0,61)	3,44 (0,60)	3,49 (0,59)
**Pengukuran luaran sekunder, rerata (SD)**								
Subskala internalisasi SATAQ-3[g] (kisaran 1-5)	2,83 (0,82)	2,85 (0,83)	– 0,62 (1843)	0,53	2,82 (0,77)	2,66 (0,84)	2,84 (0,75)	2,65 (0,78)
Subskala afek positif dari PANAS-C[h] (kisaran 1-5)	3,74 (0,67)	3,75 (0,66)	– 0,03 (1823)	0,97	3,73 (0,70)	3,70 (0,67)	3,72 (0,70)	3,74 (0,67)
Subskala afek negatif dari PANAS-C[h] (kisaran 1-5)	2,87 (0,61)	2,89 (0,62)	– 0,97 (1833)	0,33	2,76 (0,65)	2,81 (0,66)	2,83 (0,67)	2,81 (0,66)
Ketidakpuasan warna kulit (kisaran 0-8)	1,34 (1,17)	1,29 (1,10)	0,94 (1835)	0,35	1,28 (1,10)	1,14 (1,02)	1,24 (1,06)	1,16 (1,02)

[a]Waktu 1,awal
[b]Waktu 2, 1 hari pasca-intervensi.
[c]Waktu 3, 1 bulan pasca-intervensi.
[d]Kelompok intervensi.
[e]2-tailed.
[f]*Body Esteem Scale for Adolescents and Adults* [g]*Sociocultural Attitudes Towards Appearance Questionnaire.*
[h]*Positive and Negative Affect Schedule for Children.*

Perhitungan *power* menunjukkan bahwa dengan mempertimbangkan total sampel 1.847 peserta, tiga titik waktu, lima hasil, dan korelasi antara pengukuran berulang yang berkisar antara r=0,5 dan r=0,8, dicapai rentang *power* antara 99% dan 100% untuk mendeteksi ukuran efek kecil (sebagian η^2=0,10) dan sedang (sebagian η^2=0,6), dengan mempertimbangkan *error* α sebesar 0,05 (η^2=0,01 mengindikasikan efek kecil; η^2=0,06 mengindikasikan efek sedang; η^2=0,14 menunjukkan efek besar [62]). Oleh karena itu, analisis ini menunjukkan *power* yang cukupuntuk penelitian ini.

Adherensi

Selanjutnya dilakukan penilaian parameter adherensi terhadap intervensi.Rerata peserta intervensi menonton 5 dari 6 video dan melengkapi 14 dari 18 kegiatan. Lihat Lampiran Multimedia 7 untuk kepatuhan pada setiap video dan aktivitas. Penghitungan secara akurat jumlah rerata waktu yang digunakan peserta di kelompok intervensi untuk menyimak keseluruhan intervensi karena waktu yang dihabiskan untuk intervensi untuk masing-masing dari enam video untuk sebagian banyak peserta (n=401) melebihi satu jam, mengindikasikan peserta tidak menutup tab survei setelah menonton video dan menyelesaikan aktivitas terkait.

Pengujian Hipotesis dan Analisis Post-hoc

Luaran *Trait*

Kepuasan Tubuh

Tidak terdapat efek signifikan dari kelompok acak *(randomized group)* untuk *trait body dissatisfaction* menurut LMM dengan nilai awal kepuasan tubuh sebagai kovariat; faktor dua tingkat antarsubjek *(two-level between-subjects factor)*; waktu studi (T2, T3) sebagai faktor pengukuran berulang dua tingkat (*two-level repeated measures factor)*' tiga interaksi dua arah, dan satu interaksi tiga arah. Tidak terdapat efek signifikan berdasarkan interaksi waktu dan terdapat efek signifikan dari waktu. Tes –2LL membandingkan model lengkap dan model tanpa efek kelompok acak dan interaksinya signifikan sehingga menunjukkan bahwa kelompok acak berkontribusi signifikan terhadap model (Tabel 3).

Tabel 3. Hasil *trait* menurut Linear Mixed Models (LMM) yang dieksekusi dengan 21 dimensi dan struktur kovarian tidak berstruktur.

		Trait Body Dissatisfaction **(Kepuasan Tubuh Permanen)**		
−2 *Restricted Log Likelihood*	2778,823			
Uji efek tetap tipe III				
	Numerator, Denominator *df*	*F*	*P* value (REML) [a]	Partial η2
Kelompok	1. 1803,89	1,28	0,26	0,0007
Waktu (T2, T3)	1. 1743,05	46,47	<0,001	0,0259
Kelompok*Waktu	1. 1743,05	1,26	0,26	0,0007
Awal (kovariat)	1. 1804,98	2776,98	<0,0001	0,6060
Kelompok*Kovariat	1. 1804,98	0,81	0,37	0,0004
Waktu*Kovariat	1. 1744,18	43,07	<0,001	0,0241
Kelompok*Waktu*Kovariat	1. 1744,18	0,15	0,70	0,0001
−2 uji *Log Likelihood* untuk efek grup				
	Chi-square	df	*Critical Chi-square*	*P value*
	19,45	4	9,49	<0,001
		**Internalisasi *Trait***		
−2 *Restricted Log Likelihood*	5574,82			
Uji efek tetap tipe III				
	Numerator, Denominator *df*	*F* test	*P* value (REML)	Partial η2
Kelompok	1. 1808,10	2,00	0,16	0,0011
Waktu (T2, T3)	1. 1748,60	15,55	<0,001	0,0088
Kelompok*Waktu	1. 1748,60	4,92	0,03	0,0028
Awal (kovariat)	1. 1805,26	1816,57	<0,001	0,5015
Kelompok*Kovariat	1. 1805,26	0,64	0,42	0,0003
Waktu* Kovariat	1. 1746,10	15,21	<0,001	0,0086
Kelompok*Waktu*Kovariat	1. 1746,10	6,62	0,01	0,0037
− 2 uji *Log Likelihood* untuk efek grup				

	Chi square	df	*Critical Chi-square*	*P* value
	45,06	4	9,49	<0,001
***Trait* Ketidakpuasan Warna Kulit**				
−2 Restricted Log Likelihood	8789,92			
Uji efek tetap tipe III				
	Numerator, Denominator *df*	*F* test	*P* value (REML)	Partial η2
Group	1. 1810,38	1,33	0,25	0,0007
Time (T2, T3)	1. 1764,73	3,17	0,07	0,0017
Group*Time	1. 1764,73	0,19	0,66	0,0001
Baseline (kovariat)	1. 1812,04	1007,41	<0,001	0,3573
Group*Kovariat	1. 1812,04	0,60	0,44	0,0003
Time*Kovariat	1. 1766,30	6,37	0,01	0,0035
Group*Time*Kovariat	1. 1766,30	2,25	0,13	0,0012
−2 uji Log Likelihood untuk efek grup				
	Chi square	df	*Critical Chi-square*	*P* value
	11,2	4	9,49	0,03
***Trait Positive Mood* (Suasana Hati Positif Permanen)**				
−2 *Restricted Log Likelihood*	4490,78			
Uji efek tetap tipe III				
	Numerator, Denominator *df*	*F* test	*P* value (REML)	Partial η2
Kelompok	1. 1785,34	0,958	0,33	0,0005
Waktu (T2, T3)	1. 1729,25	11,56	<0,001	0,0066
Kelompok*Waktu	1. 1729,25	1,362	0,24	0,0007
Awal(kovariat)	1. 1788,42	1917,33	<0,001	0,5173
Kelompok*Kovariat	1. 1788,42	0,93	0,33	0,0005
Waktu*Kovariat	1. 1732,60	10,77	0,001	0,0061
Kelompok*Waktu*Kovariat	1. 1732,60	0,77	0,38	0,0004
− 2 uji Log Likelihood untuk efek grup				
	Chi square	df	Critical Chi-square	*P* value

	4,48	4	9,49	0,41
***Trait Negative Mood* (Suasana Hati Negatif)**				
− 2 *Restricted Log Likelihood*	4574,02			
Uji efek tetap tipe III				
	Numerator, Denominator *df*	*F* test	*P* value (REML)	Partial η2
Kelompok	1. 1812,85	0,00	0,97	0,0000
Waktu (T2, T3)	1. 1759,46	0,67	0,41	0,0004
Kelompok*Waktu	1. 1759,46	0,44	0,51	0,0002
Awal(kovariat)	1. 1812,47	1773,33	<0,001	0,4945
Kelompok*Kovariat	1. 1812,47	0,00	0,99	0,0000
Waktu* Kovariat	1. 1759,19	0,041	0,84	0,00002
Kelompok*Waktu*Kovariat	1. 1759,19	1,60	0,20	0,0009
− 2 uji Log Likelihood untuk efek grup				
	Chi square	*df*	*Critical Chi-square*	*P* value
	9,28	4	9,49	0,06

[a]*Restricted maximum likelihood*.

ANCOVA *pre-planned* menunjukkan efek non-signifikan dari *kelompok acak* pada rerata kepuasan tubuh di T2 ($F_{1, 1760}$=0,388, *P*=0,533, partial η2=0,000), dengan mengontrol kepuasan tubuh di T1 ($F_{1, 1760}$=2694,31, *P* <0,001, partial η^2=0,605). Namun, ANCOVA menunjukkan efek yang signifikan dari *kelompok acak* pada kepuasan tubuh di T3 ($F_{1, 1781}$=9,02 , *P*=0,005, partial η2=0,005) ketika mengontrol kepuasan tubuh di T1 ($F_{1, 1781}$=1868,83, *P*<0,001, partial η^2=0,512), dengan catatan kelompok intervensi menunjukkan tingkat kepuasan tubuh yang jauh lebih signifikan jika dibandingkan dengan kelompok kontrol (Tabel 2, Figur 2). Besaran efek untuk *kelompok acak* kecil.

Gambar 2. *Trait* kepuasan tubuh dari waktu ke waktu untuk kedua kelompok.

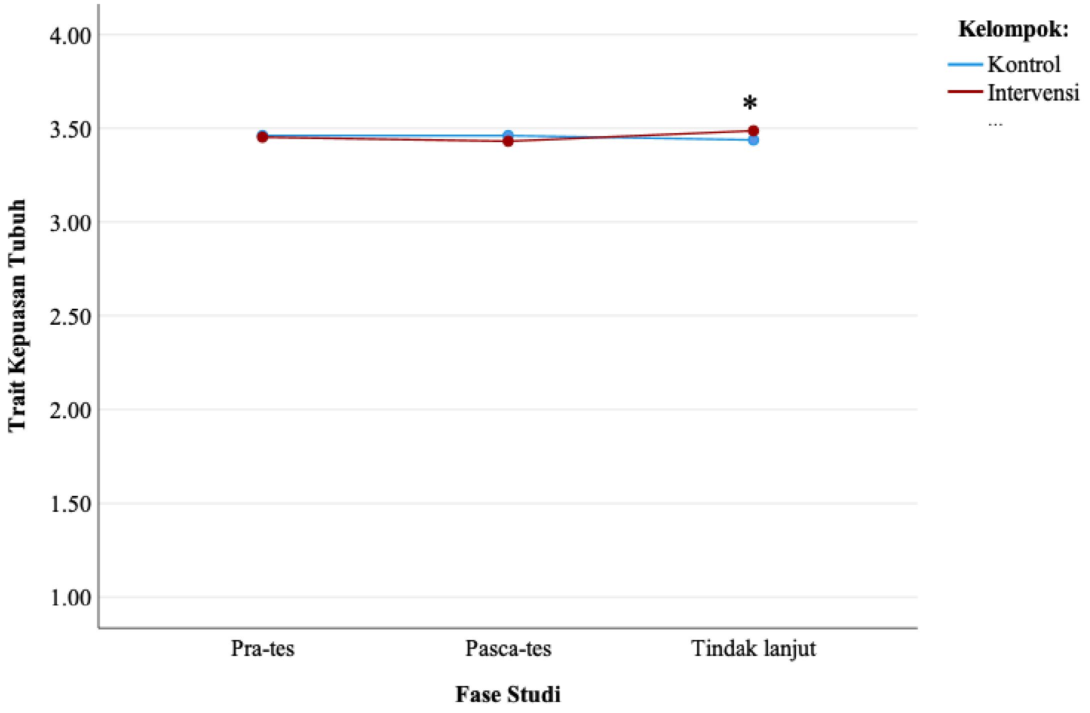

*Menunjukkan efek ANCOVA yang signifikan dari *kelompok acak* pada titik waktu tertentu.

Pengukuran ANOVA berulang post-hoc menunjukkan efek waktu yang tidak signifikan dari kepuasan tubuh pada kelompok kontrol ($F_{2, 870}$=1,56, P=0,20, partial η^2=0,004), meskipun terdapat efek yang signifikan pada kelompok intervensi $F_{2, 851}$=9,24, P<0,001, partial η^2=0,021), sehingga mengonfirmasi hasil sebelumnya. Secara khusus, pada kelompok intervensi, pengukuran berulang post-hoc ANOVA menunjukkan peningkatan signifikan rerata kepuasan tubuh antara T1 dan T3 (95% CI −0,074 hingga −0,011) serta antara T2 dan T3 (95% CI −0,076 hingga −0,028), sesuai dengan analisis sebelumnya (Tabel 2).

Internalisasi

Metode LMM dengan internalisasi awal sebagai kovariat, kelompok acak sebagai faktor antarsubjek, fase studi (T2, T3) sebagai faktor dalam-subjek, tiga *two-way interaction*, dan satu *three-way interaction* mendapatkan efek tidak signifikan satu kelompok, kelompok yang signifikan atas dasar waktu

interaksi, dan pengaruh waktu yang signifikan. Tes –2LL signifikan sehingga menunjukkan bahwa kelompok acak berkontribusi signifikan terhadap model (Tabel 3).

ANCOVA *pre-planned* menunjukkan efek yang signifikan dari kelompok acak pada internalisasi baik pada T2 ($F_{1,\,1758}$=40,56, P<0,001, partial η^2=0,022), dan pada T3 ($F_{1,\,1782}$=54,03, P<0,001, partial η^2=0,03), serta mengontrol tingkat kepuasan tubuh pada T1 $F_{1,\,1758}$=1514,30, P<0,001, partial η^2=0,46; $F_{1,\,1782}$=1332,45, P<0,001, partial η^2=0,428) · Kelompok intervensi menunjukkan tingkat internalisasi yang lebih rendah baik pada T2 maupun T3, dibandingkan dengan kelompok kontrol (Tabel 2, Gambar 3). Ukuran efek untuk kelompok acak kecil.

Gambar 3. Internalisasi *trait* dari waktu ke waktu untuk kedua kelompok.

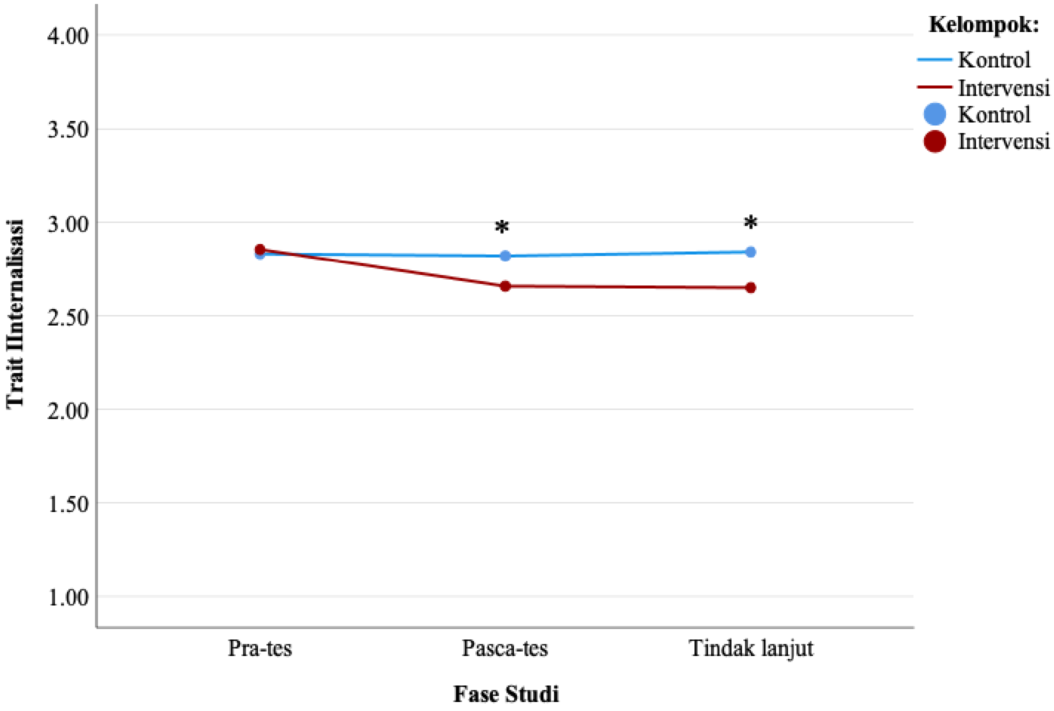

*Menunjukkan efek ANCOVA yang signifikan dari kelompok acak pada titik waktu tertentu.

Sejalan dengan hasil sebelumnya, pengukuran berulang post-hoc ANOVA tidak menunjukkan efek waktu yang signifikan pada kelompok kontrol ($F_{2,\,871}$=0,58, P=0,56, partial η^2=0,001), sementara terdapat juga efek signifikan yang hadir pada kelompok intervensi ($F_{2,\,849}$=41,24, P<0,001, partial η^2=0,09). Secara khusus, pengukuran berulang post-hoc ANOVA menunjukkan penurunan yang signifikan dalam

internalisasi rerata antara T1 dan T2 (95% CI 0,15-0,24) serta antara T1 dan T3 (95% CI 0,15-0,25) dalam kondisi intervensi (Tabel 2).

Ketidakpuasan Warna Kulit

LMM lengkap yang dilakukan terhadap ketidakpuasan warna kulit menunjukkan pengaruh kelompok yang tidak signifikan, kelompok pengaruh yang tidak signifikan berdasarkan waktu interaksi, dan pengaruh waktu yang signifikan. Uji –2LL yang membandingkan model-model dengan dan tanpa kelompok acak adalah signifikan, menunjukkan bahwa kelompok acak berkontribusi secara signifikan terhadap varians dalam variabel dependen (Tabel 3).

ANCOVA *pre-planned* menunjukkan pengaruh yang signifikan dari kelompok acak terhadap ketidakpuasan warna kulit pada T2 ($F_{1, 1744}$=8,05, P<0,01, partial η^2=0,005), serta mengontrol skor T1 ($F_{1, 1744}$=813,27, P<0,001, partial η^2=0,318), sedangkan kelompok intervensi menunjukkan tingkat ketidakpuasan warna kulit yang lebih rendah jika dibandingkan dengan kelompok kontrol (Tabel 2, Figur 4). Ukuran efek untuk kelompok acak kecil. Kelompok acak tidak memiliki pengaruh yang signifikan pada T3 ($F_{1, 1771}$=2,77, P=0,09, partial η^2=0,002) ketika mengontrol skor T1 ($F_{1, 1771}$=640,36, P<0,001, partial η^2=0,266).

Gambar 4. *Trait* ketidakpuasan warna kulit dari waktu ke waktu untuk kedua kelompok.

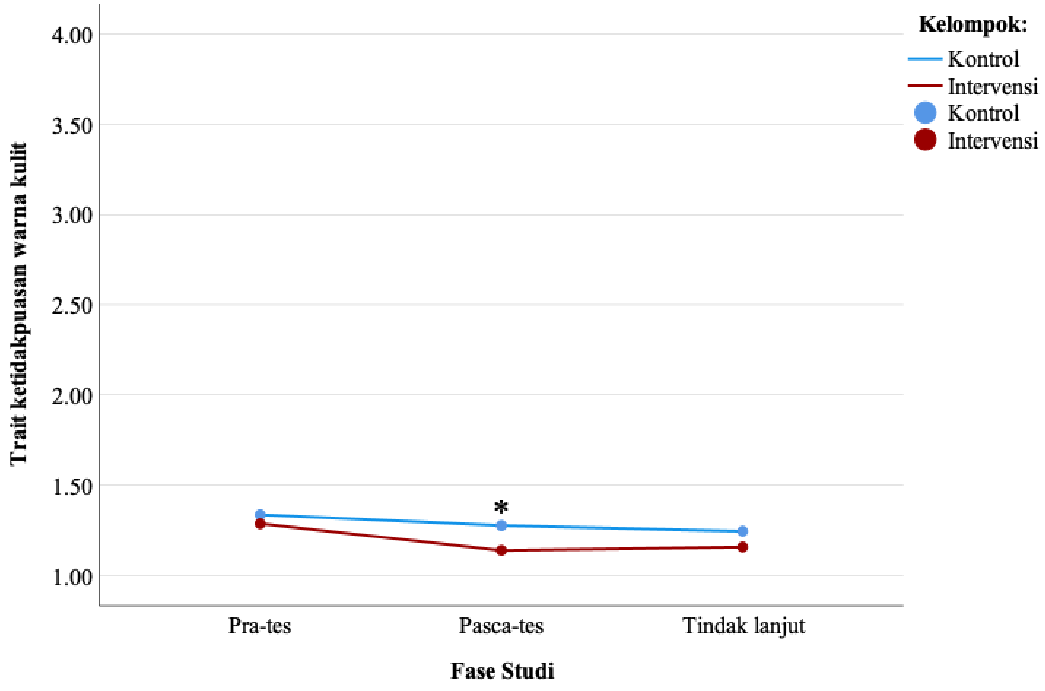

*Mengindikasikan efek ANCOVA yang signifikan dari *kelompok acak* pada titik waktu tertentu.

Pengukuran berulang post-hoc ANOVA menunjukkan tidak ada pengaruh waktu yang signifikan pada kelompok kontrol ($F_{2,\,861}$=2,13, P=0,12, partial η^2=0,005), dan pengaruh yang signifikan pada kelompok intervensi ($F_{2,\,844}$=10,51, P<0,001, partial η^2=0,024). Pada kelompok intervensi, pengukuran berulang post-hoc ANOVA menunjukkan penurunan ketidakpuasan warna kulit yang signifikan antara T1 dan T2 (95% CI 0,08-0,22) serta antara T1 dan T3 (95% CI 0,06-0,20) (Tabel 2).

Suasana Hati Negatif

LMM dengan *baseline* suasana hati negatif sebagai kovariat, kelompok acak sebagai faktor dua tingkat antarsubjek, fase studi (T2, T3) sebagai faktor pengukuran berulang dua tingkat, tiga *two-way interaction*, dan satu *three-way interaction* menunjukkan pengaruh kelompok yang non-signifikan, serta kelompok yang non-signifikan berdasarkan waktu interaksi, dan pengaruh waktu yang signifikan. Tes –2LL yang membandingkan model lengkap dan model tanpa efek kelompok acak dan interaksinya tidak

signifikan, mengindikasikan bahwa kelompok acak tidak berkontribusi signifikan terhadap model (Tabel 3).

ANCOVA *pre-planned* mengkonfirmasi hasil sebelumnya menemukan efek non-signifikan dari kelompok acak pada suasana hati negatif pada T2 ($F_{1, 1742}$=1,47, P=0,23, partial η^2=0,001) dan pada T3 ($F_{1, 1772}$=1.85, P=0,173, partial η^2=0,001) ketika mengontrol skor T1 ($F_{1, 1742}$=1367.26, P<0,001, partial η^2=0,440; $F_{1, 1772}$=1316,09, P<0,001, partial η^2=0,426).

Pengukuran berulang post-hoc ANOVA menunjukkan efek waktu yang signifikan pada kelompok kontrol ($F_{2, 865}$=19,32, P<0,001, partial η^2=0,043), serta efek signifikan pada kelompok intervensi ($F_{2, 840}$=10,70, P<0,001, parsial η^2=0,025). Secara khusus, pengukuran berulang post-hoc ANOVA menunjukkan penurunan suasana hati negatif yang signifikan antara T1 dan T2 (95% CI 0,07-0,14) serta antara T1 dan T3 (95% CI 0,001-0,072) pada kelompok kontrol. Penurunan signifikan suasana hati negatif yang serupa terlihat antara T1 dan T2 (95% CI 0,04-0,11) serta antara T1 dan T3 (95% CI 0,04-0,11) pada kelompok intervensi (Tabel 2).

Suasana Hati Positif

LMM yang dilakukan pada suasana hati positif menunjukkan efek kelompok yang non-signifikan, serta efek waktu yang non-signifikan, dan interaksi kelompok dengan waktu yang non-signifikan. Tes –2LL juga non-signifikan, mengindikasikan bahwa kelompok acak tidak berkontribusi secara signifikan terhadap model (Tabel 3).

ANCOVA *pre-planned* mengkonfirmasi hasil ini, yaitu ketika menemukan efek yang non-signifikan dari kelompok acak pada suasana positif di kedua T2 ($F_{1, 1734}$=0,61, P=0,43, partial η^2=0,000) dan T3 ($F_{1, 1758}$=1,20, P=0,27, partial η^2 =0,001), sambil mengontrol skor T1 ($F_{1, 1734}$=1665,95, P<0,001, parsial η^2=0,490; $F_{1, 1758}$=1306,27, P<0,001, partial η^2=0,426). Pengukuran berulang post-hoc ANOVA tidak menunjukkan efek waktu yang signifikan baik untuk kelompok kontrol ($F_{2, 860}$=0,82, P=0,44, partial η^2=0,002) maupun kelompok intervensi ($F_{2, 834}$=1,67, P=0,18, partial η^2=0,004) sehingga mengkonfirmasi hasil sebelumnya.

Analisis Dosis-respons

Efek dosis-respons pada hasil *trait* di T2 dan T3 pada kelompok intervensi diperiksa dengan menjalankan analisis regresimultipeldengan skor keterlibatan berkode Helmert (yaitu jumlah video yang ditonton) sebagai variabel independen. Analisis menunjukkan tidak ada dosis-respons untuk semua hasil *trait* pada semua titik waktu (kepuasan tubuh pada T2 $F_{5, 865}$=0,894, P=0,48) dan T3 ($F_{5, 887}$=0,665, P=0,65); internalisasi pada T2 ($F_{5, 864}$=1,62, P=**0,**15) dan T3 ($F_{5, 887}$=0,95, P=0,45); ketidakpuasan warna kulit pada T2 ($F_{5, 859}$=1,4, P=0,23) dan T3 ($F_{5, 884}$=1,07, P=0,38); suasana hati negatif pada T2 ($F_{5, 860}$=0,424, P=0,83) dan T3 ($F_{5, 886}$=0,64, P=0,67); suasana hati positif pada T2 ($F_{5, 859}$=1,65, P=0,14) dan T3 ($F_{5, 884}$=1,56, P=0,17)).

Luaran *State*

State Body Satisfaction

Enam sampel *t* dependen digunakan untuk membandingkan tingkat *state body satisfaction* sebelum dan segera sesudah menonton masing-masing dari enam video *Warna-Warni Waktu*. Uji *t* menemukan bahwa setiap video berhasil meningkatkan *state body satisfaction* pada kelompok intervensi (Tabel 4, Figur 5).

Tabel 4. Hasil *state*.

		State Body Satisfaction		
Video	Rerata sebelum (SD)[a]	Rerata sesudah (SD)	Sampel *t* berpasangan (*df*)[a]	*P* value
1	66,71 (23,61)	73,65 (21,47)	−12,75 (811)	<0,001
2	72,17 (21,40)	77,11 (19,72)	−13,32 (801)	<0,001
3	74,87 (19,94)	80,62 (18,43)	−15,40 (799)	<0,001
4	76,17 (19,52)	80,18 (18,62)	−12,82 (798)	<0,001
5	76,97 (20,10)	82,20 (18,38)	−15,07 (796)	<0,001
6	77,92 (19,98)	83,61 (18,11)	−15,47 (795)	<0,001
		State Mood		
1	69,45 (23,67)	73,76 (22,60)	−10,04 (812)	<0,001
2	71,35 (23,16)	75,10 (22,69)	−9,97 (801)	<0,001
3	73,92 (22,22)	78,30 (21,18)	−12,87 (798)	<0,001

4	74,46 (21,89)	78,54 (21,27)	−12,34 (798)	<0,001
5	75,16 (22,04)	79,64 (21,18)	−13,33 (796)	<0,001
6	75,66 (22,49)	81,42 (20,60)	−13,79 (794)	<0,001

[a]2-tailed.

Gambar 5. Plot rerata untuk *state body satisfaction* pada kelompok intervensi dengan rerata sebelum dan sesudah video untuk setiap video. Untuk *P* value, lihat Tabel 4 dan 5.

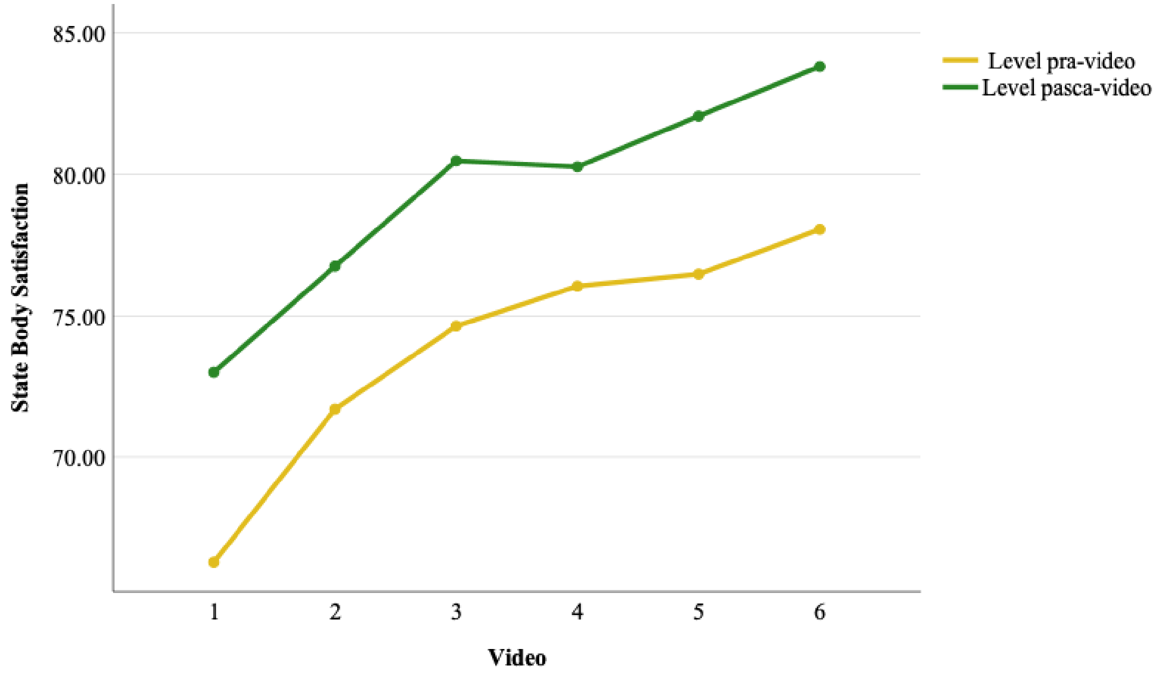

Analisis kumulatif menunjukkan efek interaksi yang signifikan pada ANOVA 2x6, mengindikasikan bahwa besarnya peningkatan kepuasan tubuh berbeda antara video. Syarat interaksi juga menunjukkan tren kuadratik yang signifikan (Tabel 5). Uji sampel *t* dependen post-hoc yang membandingkan *gain score* pra-pasca antara titik-titik waktu yang berdekatan menunjukkan bahwa peningkatan kepuasan tubuh secara signifikan lebih besar untuk video 1 dan 3 daripada video 2. Demikian juga, peningkatan kepuasan tubuh secara signifikan lebih besar untuk video 3 dan 5 dibandingkan dengan video 4. Video 6 menunjukkan *gain score* yang jauh lebih besar daripada video 5 (Tabel 5).

Tabel 5 . Analisis kumulatif untuk hasil *state*.

State Body Satisfaction					
**2x6 ANOVA (Efek dalam subjek : Greenhouse-Geisser)**					
			Numerator, Denominator *df*	*F*	*P* value
Pra-Pasca			1. 662	502,6	<0,001
Video			1. 662	150,08	<0,001
Pra-Pasca*Video			1. 662	4,86	<0,001
**Tren untuk efek interaksi**					
Linier			1. 662	1,48	0,22
Kuadratik			1. 662	8,85	0,003
Kubik			1. 662	0,20	0,65
**Poin *t* yang berdekatan untuk *gain score***					
	Rerata *(SD)*	Perbandingan perolehan skor	*df*	*t*	*P* value[a]
Gain score untuk video 1	6,93 (15,5)	Video 1 vs Video 2	764	2,86	0,004
Gain score untuk video 2	4,94 (10,5)	Video 2 vs Video 3	763	−1,96	0,050
Gain score untuk video 3	5,75 (10,6)	Video 3 vs Video 4	766	4,17	<0,001
Gain score untuk video 4	4,01 (8,8)	Video 4 vs Video 5	761	−2,74	0,006
Gain score untuk video 5	5,22 (9,8)	Video 5 vs Video 6	760	−0,78	0,432
Gain score untuk video 6	5,68 (10,4)				
**Skor pra-video RM ANOVA (Efek dalam subjek : Sphericity diasumsikan)**					
			Numerator, Denominator *df*	*F*	*P* value
Video			5. 3685	120,30	<0,001
**Kontras berulang**					
Video 1 vs Video 2			1. 737	71,93	<0,001
Video 2 vs Video 3			1. 737	52,05	<0,001
Video 3 vs Video 4			1. 737	12,03	<0,001
Video 4 vs Video 5			1. 737	3,20	0,057
Video 5 vs Video 6			1. 737	10,10	0,002
**Skor pasca-video RM ANOVA (Efek dalam subjek : Sphericity diasumsikan)**					
			Pembilang, Penyebut *df*	*F*	Nilai *P*

Video			5. 3325	118,43	<0,001
**Kontras berulang**					
Video 1 vs Video 2			1. 665	46,62	<0,001
Video 2 vs Video 3			1. 665	73,10	<0,001
Video 3 vs Video 4			1. 665	0,46	0,50
Video 4 vs Video 5			1. 665	26,10	<0,001
Video 5 vs Video 6			1. 665	20,61	<0,001
***State* Suasana Hati**					
**2x6 ANOVA (Efek dalam subjek : Greenhouse-Geisser)**					
			Numerator, Denominator *df*	*F*	*P* value
Pra-Pasca			1. 664	408,13	<0,001
Video			1. 664	48,14	<0,001
Pra-Pasca*Video			1. 664	11,16	<0,001
**Tren untuk efek interaksi**					
Linier			1. 664	3,77	0,053
Kuadratik			1. 664	30,22	<0,001
Kubik			1. 664	5,86	0,016
**Tes-T Poin yang berdekatan untuk *gain score***					
	Rerata *(SD)*	Perbandingan *gain score*	*df*	*t*	*P* value[a]
Gain score untuk video 1	4,31 (12,2)	Video 1 vs Video 2	765	1,25	0,21
Gain score untuk video 2	3,75 (10,6)	Video 2 vs Video 3	763	−1,34	0,18
Gain score untuk video 3	4,35 (9,6)	Video 3 vs Video 4	766	0,16	0,87
Gain score untuk video 4	4,07 (9,3)	Video 4 vs Video 5	761	−0,93	0,35
Gain score untuk video 5	4,47 (9,5)	Video 5 vs Video 6	760	−2,70	0,007
Gain score untuk video 6	5,75 (11,7)				
**Skor pra-video RM ANOVA (Efek dalam subjek : Sphericity diasumsikan)**					
			Numerator, *df*	*F*	*P* value
Video			5. 3690	46,67	<0,001
**Kontras berulang**					
Video 1 vs Video 2			1. 738	37,60	<0,001
Video 2 vs Video 3			1. 738	17,02	0,36

Video 3 vs Video 4	1. 738	0,84	0,38
Video 4 vs Video 5	1. 738	0,78	0,38
Video 5 vs Video 6	1. 738	0,76	
**Skor pasca-video RM ANOVA (Efek dalam subjek : Sphericity diasumsikan)**			
	Numerator, *df*	*F*	*P* value
Video	5. 3330	33,81	<0,001
**Kontras berulang**			
Video 1 vs Video 2	1. 666	4,80	0,029
Video 2 vs Video 3	1. 666	24,52	<0,001
Video 3 vs Video 4	1. 666	0,23	0,63
Video 4 vs Video 5	1. 666	2,402	0,12
Video 5 vs Video 6	1. 666	12,90	<0,001

[a]2-sided.

Pengukuran berulang ANOVA pada skor pra-video signifikan, dan kontras berulang menunjukkan peningkatan yang signifikan dan progresif dari skor kepuasan tubuh pra-video seiring peserta melanjutkan menonton video-videonya (Tabel 5, Tabel 4, Figur 5). Demikian halnya, pengukuran berulang ANOVA pada skor pasca-video signifikan, serta ditemukan adanya peningkatan progresif skor kepuasan tubuh pasca-video seiring peserta melanjutkan intervensi, dengan perkecualian dari gerak datar/*plateau* antara video 3 dan 4 (Tabel 5, Tabel 4 **,** Figur 5).

State Mood

Enam uji sampel *t* dependen yang digunakan untuk membandingkan tingkat suasana hati segera sebelum dan sesudah menonton masing-masing dari enam video menunjukkan bahwa setiap video berhasil meningkatkan suasana hati kelompok intervensi (Tabel 4, Gambar 6).

Gambar 6. Plot rerata untuk *state mood* pada kelompok intervensi dengan rerata sebelum dan sesudah video untuk setiap video. Untuk *P* value, lihat Tabel 4 dan 5.

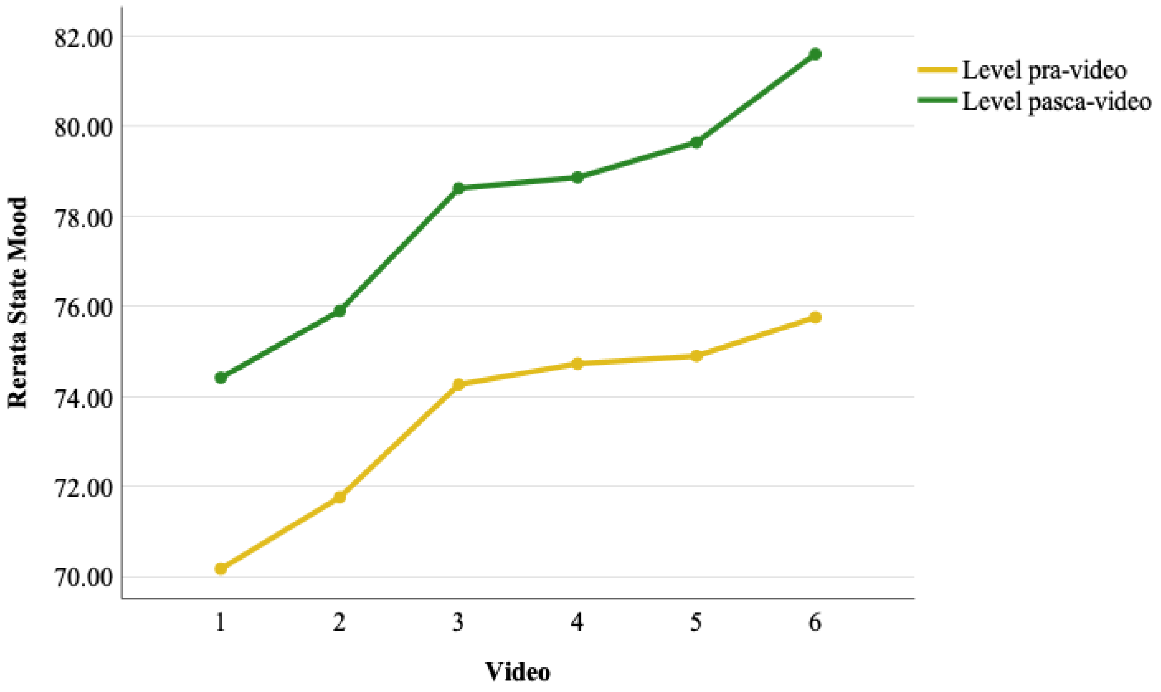

Analisis kumulatif menunjukkan efek interaksi yang signifikan dalam ANOVA 2x6, yang menunjukkan bahwa besarnya perubahan *mood* berbeda di antara video. Syarat interaksi juga menunjukkan tren kuadratik dan kubik yang signifikan (Tabel 5). Tes sampel *t* dependen post-hoc yang membandingkan *gain score* pra-pasca antara titik waktu yang berdekatan semuanya tidak signifikan, kecuali untuk video 6, yang menunjukkan peningkatan *mood* yang jauh lebih besar jika dibandingkan dengan video 5 (Tabel 5).

Pengukuran berulang ANOVA pada skor pra-video adalah signifikan, dan kontras berulang menunjukkan peningkatan skor *mood* pra-video yang signifikan dan progresif untuk video 1, 2, dan 3 (Tabel 5, Tabel 4, Gambar 6). Pengukuran berulang ANOVA pada skor pasca-video juga signifikan, dengan ditemukannya peningkatan progresif dan signifikan yang serupa dari skor *mood* pasca-video untuk semua video, dengan perkecualian dari gerak *plateau* antara video 3 dan 5 (Tabel 5, Tabel 4, Figur 6) .

Analisis Eksploratif

Berdasarkan hasil penelitian ini, analisis eksploratif dilakukan untuk menguji apakah efek tunda yang tampak pada *trait body dissatisfaction* dapat dimediasi oleh efek langsung intervensi terhadap internalisasi. Hasil ini mendukung hubungan variabel-variabel yang disebutkan dalam *Tripartite Influence Model* [44] yang menjadi landasan teoretis *Warna-Warni Waktu*. Model yang dimediasi eksplorasi *(exploratory mediated model)* dengan *kelompok acak* sebagai variabel independen dikotomis, kepuasan tubuh pada T1 sebagai kovariat, perubahan internalisasi dari T1 ke T2 sebagai mediator, dan kepuasan tubuh pada T3 sebagai variabel dependen menunjukkan efek yang signifikan (R^2=0,54, $F_{3, 1719}$=680,58, P<0,001).

Kelompok acak secara signifikan berhubungan dengan mediator (R^2=0,04, $F_{2, 1720}$=34,25, β=0,18, P<0,001), di mana peserta dalam kelompok intervensi mengalami penurunan internalisasi yang jauh lebih besar antara T1 dan T2 jika dibandingkan dengan kelompok kontrol. Kovariat (kepuasan tubuh pada T1) juga memprediksi mediator secara signifikan (β=-0,1450, P<0,001). Setelahnya, mediator secara signifikan memprediksi variabel dependen (β=0,16, P<0,001), dengan peserta yang mengalami penurunan internalisasi yang lebih besar dari T1 ke T2 juga mengalami kepuasan tubuh yang lebih tinggi di T3. Kovariat juga secara signifikan memprediksi variabel dependen (β=0,71, P<0,001) (Gambar 7).

Gambar 7. Analisis eksplorasi mediasi.

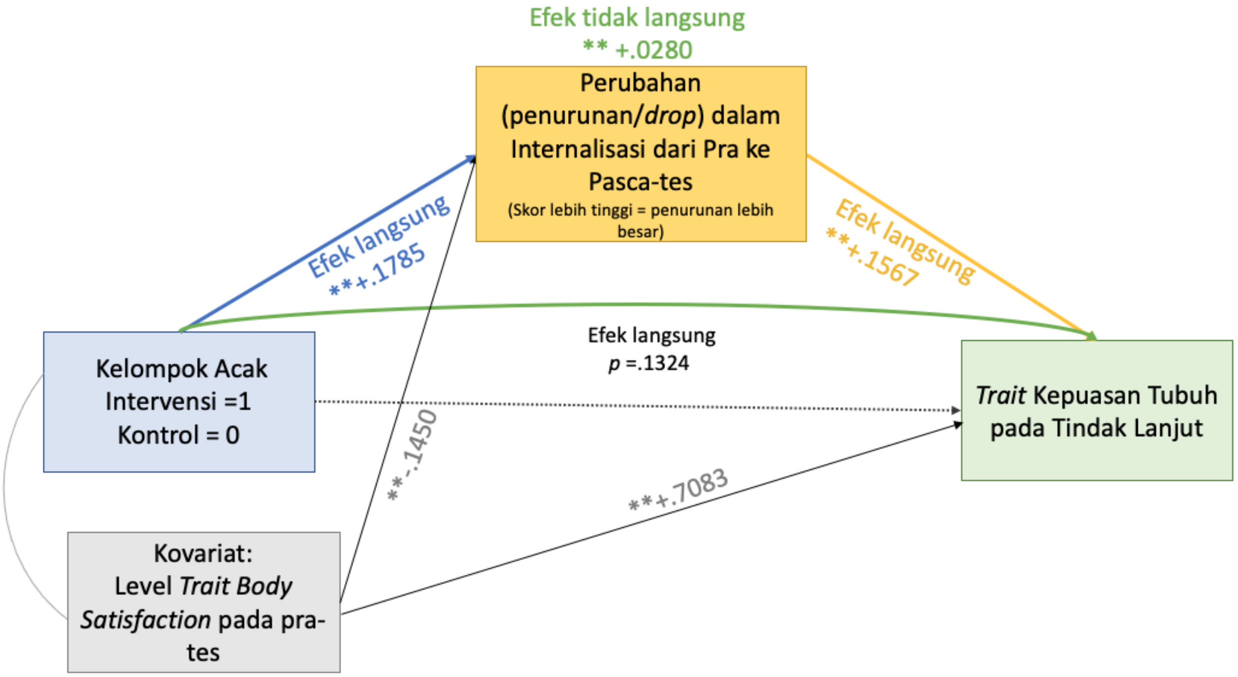

**Menunjukkan efek yang signifikan (*P*<0,001) dari efek spesifik tersebut.
Untuk efek yang signifikan, beta dilaporkan pada figur.
Untuk efek yang tidak signifikan, *P* value dilaporkan pada figur.

Model ini menunjukkan hubungan yang lengkap, dengan efek tidak langsung kelompok acak pada kepuasan tubuh di T3 menjadi positif dan signifikan (β=0,03, 95% CI 0,017-0,041), dan efek langsung menjadi tidak signifikan (β=0,03, *P*=0,13) (Figur 7). Secara keseluruhan, model mediasi ini mengindikasikan bahwa peserta dalam kondisi intervensi mengalami kepuasan tubuh yang jauh lebih tinggi pada T3, yang sepenuhnya dimediasi oleh penurunan internalisasi antara T1 dan T2 yang disebabkan oleh intervensi.

Diskusi

Temuan Utama

Warna-Warni Waktu, sebuah intervensi berbasis media sosial yang bertujuan meningkatkan citra tubuh di kalangan remaja perempuan Indonesia, secara signifikan meningkatkan *trait body satisfaction* dan mengurangi internalisasi penampilan ideal dan ketidakpuasan terhadap warna kulit dibandingkan

kelompok kontrol. Intervensi tidak menunjukkan dampak pada *trait mood* dibandingkan kelompok kontrol. Perbaikan *state body satisfaction* dan *state mood* terbukti bagi peserta dalam kelompok intervensi segera setelah menonton setiap video.

Efektivitas Intervensi terhadap Hasil *Trait*

Intervensi berbasis bukti yang terukur dan hemat biaya untuk mengendalikan rasa ketidakpuasan tubuh dibutuhkan dan diminati remaja perempuan Indonesia [41]. *Warna-Warni Waktu* memenuhi kebutuhan ini, menyediakan pilihan yang mudah diakses, berbasis web, sesuai budaya remaja perempuan di Indonesia untuk melawan tekanan akan penampilan yang dihadapi dalam kehidupan sehari-hari. *Warna-Warni Waktu*, yang dikembangkan oleh pakar citra tubuh lokal dan internasional, pemimpin industri penciptaan konten citra tubuh untuk anak muda, agensi kreatif dan digital yang berbasis di Jakarta dan Inggris, dan remaja perempuan Indonesia, telah menunjukkan penerimaan yang kuat oleh kalangan penonton target [46].

Studi ini menunjukkan bahwa *Warna-Warni Waktu* secara signifikan meningkatkan luaran utama *trait body satisfaction* 1 bulan setelah partisipasi intervensi, secara signifikan mengurangi internalisasi penampilan ideal pada 1 hari dan 1 bulan setelah intervensi, dan secara signifikan mengurangi ketidakpuasan warna kulit pada 1 hari pasca-intervensi dibandingkan kelompok kontrol. Efek untuk masing-masing hasil positif tersebut tidak besar sepertipada penelitian sebelumnya. Secara khusus, intervensi citra tubuh dengan panjang yang sama yang didesain untuk sampel-sampel universal juga menemukan ukuran efek yang kecil [5, 7]. Dalam penelitian citra tubuh, efek kecil dari berbagai sampel memiliki signifikansi implementasi yang cukup besar. Penelitian-penelitian tersebut, meskipun terbatas yang menggunakan sampel Indonesia, menunjukkan bahwa lebih dari separuh anak muda secara global mengalami tingkat ketidakpuasan tubuh yang rendah [1, 63, 64]. Dengan demikian, pengembangan dan penyebaran intervensi yang terukur untuk mengurangi kekhawatiran sebagai bagian dari pendekatan perawatan bertahap/*stepped-care* merupakan langkah maju yang penting, khususnya pada masyarakat di LMIC.

Menariknya, dampak *Warna-Warni Waktu* pada *trait body satisfaction* hanya terlihat satu bulan setelah keterlibatan dalam intervensi, bukan pada satu hari dan satu bulan pasca-intervensi, seperti yang dihipotesiskan. Analisis eksplorasi dari hasil ini, berdasarkan teori yang mendasari intervensi ini memberikan penjelasan yang sesuai. Secara khusus, menurut *Tripartite Influence Model* [44], analisis mediasi menilai apakah dampak tunda pada kepuasan tubuh dapat dimediasi oleh dampak langsung intervensi ini pada internalisasi penampilan ideal. Ternyata memang demikian adanya; dampak intervensi terhadap kepuasan tubuh pada tindak lanjut 1 bulan seluruhnya dimediasi oleh skor perubahan dalam internalisasi antara awal dan tindak lanjut 1 hari. Efek intervensi tunda atau yang membaik dari waktu ke waktu pada citra tubuh bukanlah hal yang jarang dalam literatur [65-67]. Dengan mempelajari efek mediasi dari faktor risiko citra tubuh (misalnya internalisasi penampilan ideal) dalam menjelaskan efek tunda ini, dengan efek mediasi lengkap seperti yang didapatkan penelitian ini, temuan penelitian ini berkontribusi kepada diskusi lebih lanjut mengenai efek tunda dari suatu intervensi dan pentingnya pengukuran luaran lanjutan.

Di samping peran internalisasi sebagai mediator pada kepuasan tubuh, hipotesis peneliti bahwa internalisasi sebagai sebuah hasil akan menurun pada 1 hari dan 1 bulan pasca-intervensi telah terkonfirmasi, sehingga memperkuat gagasan awal bahwa intervensi berhasil menargetkan faktor risiko utama ini. Demikian pula bahwa ketidakpuasan warna kulit menurun pada 1 hari setelah intervensi seperti diperkirakan di awal; namun, efek ini tidak berlanjut sampai pada pemantauan lanjutan 1 bulan. Hal ini dapat disebabkan keterbatasan alat ukur ketidakpuasan warna kulit yang tervalidasi dan sesuai. Hal ini dapat juga dikarenakan intervensi ini tidak memiliki keampuhan yang cukup untuk memberikan perubahan yang berkelanjutan mengingat betapa dalamnya ketidakpuasan warna kulit tertanam pada perempuan Indonesia.

Bertentangan dengan hipotesis kami, intervensi ini tidak berdampak signifikan pada *trait mood* positif atau negatif. Ini dapat dikarenakan *Warna-Warni Waktu* dirancang khusus untuk menargetkan faktor risiko ketidakpuasan tubuh, bukan *mood*. Hasil serupa telah disebutkan dalam intervensi citra tubuh lainnya [8, 15, 54] yang menunjukkan kebutuhan pertimbangan yang lebih besar akan faktor risiko untuk

trait mood yang rendah selama pengembangan intervensi agar dapat memicu hasil signifikan dan berkelanjutan.

Terakhir, beberapa analisis eksplorasi post-hoc (lihat Lampiran Multimedia 10) untuk gambaran lengkap) menunjukkan bahwa peserta di kelompok intervensi dengan tingkat kepuasan tubuh yang lebih rendah pada awal menunjukkan peningkatan kepuasan tubuh yang jauh lebih besar dari pra-tes ke pasca-tes, serta dari pra-tes dan tindak lanjut jika dibandingkan dengan peserta dengan tingkat kepuasan tubuh yang lebih tinggi pada *baseline*. Demikian pula, peserta dengan tingkat kepuasan tubuh yang lebih rendah pada *baseline* menunjukkan penurunan internalisasi yang lebih besar baik antara pra-tes dan pasca-tes, serta dari pra-tes ke tindak lanjut bila dibandingkan dengan peserta dengan tingkat kepuasan tubuh yang lebih tinggi pada *baseline*. Hasil analisis post-hoc eksploratif ini mengindikasikan adanya potensi *ceiling effects* bagi para peserta yang memulai intervensi dengan tingkat kepuasan tubuh yang relatif tinggi. Di sisi lain, hasil ini juga menunjukkan potensi efikasi intervensi yang lebih tinggi untuk remaja perempuan yang mengalami tingkat kepuasan tubuh yang lebih rendah.

Efek dosis-respons

Kepatuhan intervensi sangat baik dengan peserta intervensi telah menonton sekitar 5 dari 6 video dan menyelesaikan sekitar 14 dari 18 kegiatan. Hasil kepatuhan ini jauh lebih tinggi daripada yang sering dihasilkan intervensi psikologis daring [68]. Tingkat kepatuhan yang tinggi mungkin disebabkan oleh jenis kelamin, yaitu perempuan cenderung lebih patuh daripada pria [68]; penerimaan dan kesukaan yang tinggi akan intervensi tersebut di antara kelompok sasaran [46]; keterlibatan pengguna akhir dalam pengembangan *Warna-Warni Waktu*, yaitu yang dikaitkan dengan kepatuhan yang kuat dalam intervensi eHealth lainnya [69, 70]; dan/atau ‘*buy in*’ yang kuat dari peserta karena kedekatan mereka dengan peneliti rekanan dan adanya insentif finansial dalam menyelesaikan aspek lain dari penelitian. Meskipun kepatuhan yang tinggi ideal untuk menguji keefektifan intervensi, hal tersebut menghalangi kemampuan untuk menjalankan efek dosis-respons yang dapat diandalkan. Distribusi yang amat condong ke tingkat keterlibatan yang tinggi berakibat pada kurangnya *power* saat membandingkan peserta berdasarkan

jumlah video yang ditonton [71]. Meskipun kami tidak menemukan efek dosis-respons pada hasil *trait*, penting untuk dicatat bahwa temuan yang non-signifikan mungkin saja disebabkan oleh kurangnya variasi dalam skor keterlibatan di kondisi intervensi.

Efektivitas Intervensi pada Hasil *State*

Secara keseluruhan, efektivitas *Warna-Warni-Waktu* pada hasil *state* adalah baik karena setiap video menghasilkan peningkatan yang cepat dan signifikan dalam *state* kepuasan tubuh dan *mood*, yang mengindikasikan bahwa setiap video berdampak positif bagi penonton walaupun hanya singkat.

Perbandingan *gain score* dalam analisis kumulatif menunjukkan bahwa peningkatan kepuasan tubuh berbasis *state* sangat kuat untuk video 1 (pengaturan adegan), 3 (menargetkan faktor perbandingan berbasis penampilan), 5 (menargetkan faktor pembicaraan tubuh sendiri/body talk), dan 6 (menyimpulkan cerita), serta video 6 untuk peningkatan *state mood*. Menariknya, dari empat video yang menargetkan faktor risiko spesifik untuk ketidakpuasan tubuh (video 2-5), dua yang menargetkan perubahan kognitif internal adalah yang paling efektif dalam menghasilkan perubahan *state* dalam kepuasan tubuh. Hal ini mungkin karena peserta merasa dapat mengambil kendali langsung dari proses berpikir mereka untuk melawan faktor-faktor risiko ketidakpuasan tubuh ini (yaitu, mereka telah belajar bagaimana memutus mata rantai perbandingan penampilan dan melihat tubuh mereka dengan lebih penuh kasih pada saat itu), sedangkan keterampilan yang dipelajari dalam video 2 (menargetkan faktor literasi media) dan video 4 (menargetkan *comparison based teasing*) membutuhkan waktu lebih lama untuk dipraktikkan dan bermanfaat. Video 6 juga sangat efektif dalam meningkatkan kepuasan tubuh dan suasana hati. Video terakhir ini menunjukkan dunia alternatif untuk masa depan distopia yang terlihat di video 1, di mana masyarakat hidup bebas dari tekanan penampilan akibat perubahan positif sehari-hari yang dilakukan oleh seorang individu (yaitu Putri). Sehingga tidak mengherankan jikas intervensi ini meningkatkan kepuasan tubuh dan suasana hati di antara penonton karena hal tersebut menunjukkan dampak dari perubahan di level individu.

Hal penting lainnya ialah yang signifikan dan progresif dalam skor pra dan pasca-*state body satisfaction* untuk video 1-6 (mendatar antara video 4 dan 5 untuk skor pra-video dan mendatar antara video 3 dan 4 untuk skor pasca-video). Terdapat pula peningkatan progresif skor pra- dan pasca-*state mood*, khususnya antara video 1 dan 3 untuk skor pra-video, yang bertahan hingga video 6, dan mendatar antara video 3 dan 5 untuk skor pasca-video. Alasan peningkatan skor *state* sebelum setiap video karena setelah menonton video pertama para peserta merasa semakin terlibat di dalam narasi *Warna-Warni Waktu* dan mengidentifikasikan diri dengan perjuangan karakter utama. Hal ini dapat menyebabkan peserta mengalami peningkatan kepuasan tubuh dan *mood* pada saat mereka menunggu menonton setiap video. Hasil tersebut menunjukkan bahwa efek antisipasi ini menjadi semakin kuat seiring berlanjutnya peserta mengikuti serial. Demikian pula, didapatkan skor pasca-video yang semakin tinggi dalam *state body statisfaction* dan *mood* ketika peserta menonton serial ini, yang juga berpotensi terkait dengan keakraban dan ketertarikan dengan karakter dan alur cerita. Penelitian-penelitian yang terkait dengan drama seri pendidikan-hiburan menyebutkan bahwa seri-seri tersebut secara positif memengaruhi perubahan sikap dan perilaku hidup sehat, yang sebagian disebabkan oleh emosi yang dipancing oleh cerita, pengulangan yang memfasilitasi identifikasi karakter, dan konversi karakter menjadi panutan bagi penonton [72]. Lebih jauh, model kemungkinan persuasi (*likelihood model of persuasion*) menunjukkan kemungkinan identifikasi karakter dan keterlibatan dengan alur cerita sebagai elemen utama dalam membuat penonton memproses dan meresapi pesan yang dirancang [73, 74], yang dapat menjelaskan efek positif progresif dan kumulatif dalam penelitian kami.

Kekuatan

Kekuatan penting dari penelitian kami meliputi retensi peserta yang sangat baik dan kepatuhan intervensi, hal yang tidak biasa untuk uji coba intervensi eHealth [31], sehingga ikut memperkuat validitas temuan. Selain itu, karena *Warna-Warni Waktu* dirancang untuk disampaikan melalui media sosial, hal ini melibatkan remaja perempuan Indonesia di situasi yang nyaman bagi mereka dan sesuai dengan kondisi mereka menghabiskan waktu sehari-hari, sehingga mereka yang membutuhkan intervensi

tidak perlu mengakses *platform* yang tidak dikenal atau mengunduh aplikasi tertentu, yang diketahui berkontribusi terhadap atrisi tinggi [31]. Skalabilitas intervensi kesehatan di LMIC umumnya memiliki banyak tantangan dan terbatas pada intervensi skala kecil dikarenakan kurangnya strategi yang jelas untuk skalabilitas sebelum disebarluaskan [75]. Pengembangan kolaboratif multi-disiplin dalam menjangkau remaja perempuan Indonesia dan evaluasi selanjutnya dari *Warna-Warni Waktu* didasarkan pada tujuan penyebarluasan skala besar; rencana awal kami untuk menjangkau ratusan ribu remaja perempuan di Indonesia pada tahun 2022 melalui pemasaran media sosial jelas merupakan kekuatan dari intervensi ini.

Keterbatasan

Ada beberapa bidang utama yang dapat diteliti di masa depan. Pertama, keterbatasan pendanaan menghalangi kami untuk mengevaluasi dampak independen dari video intervensi versus dampak gabungan dari video dan aktivitas tambahan setelah video. Ini adalah area untuk penelitian selanjutnya. Kedua, meskipun perbandingan sosial adalah mekanisme perubahan yang berpotensi penting dalam intervensi ini sesuai dengan *Tripartite Influence Model* [44], belum ada metode pengukuran relevan yang divalidasi untuk remaja atau dewasa muda Indonesia. Dengan demikian, akan lebih baik jika metode pengukuran yang sesuai, misalnya Skala *Physical Appearance Comparison* yang direvisi [76] atau Skala *Upward and Downward Appearance* [77] divalidasi dalam konteks Indonesia dan dilibatkan dalam studi replikasi. Ketiga, dampak individual dari setiap video sebaiknya lebih dievaluasi; namun, menimbang kepatuhan peserta yang sangat baik dalam studi kami menyebabkan tidak mungkin untuk mengidentifikasi efek dosis-respons. Keempat, yang digunakan dalam penelitian ini ialah kelompok kontrol tunggu *(waitlist)* karena tidak ada pilihan intervensi yang tepat bagi kelompok kontrol aktif *(active control)* yang , sehingga tidak mungkin menyembunyikan peserta kemungkinan intervensi yang akan mereka dapat. Jika intervensi serupa dan sesuai budaya seperti ini dikembangkan di Indonesia, akan lebih bermanfaat jika menggunakan kontrol aktif saat mereplikasi penelitian. Kelima, terdapat risiko bias seleksi karena peserta direkrut melalui orangtua yang telah terlibat dan tertarik dengan penelitian ini (yaitu melalui panel penelitian yang digunakan sebelumnya oleh peneliti rekanan) dan anak

perempuannya langsung terpilih bukan dengan diacak. Keenam, temuan pada tindak lanjut satu bulan untuk *Warna-Warni Waktu* cukup menggembirakan. Periode waktu tindak lanjut ini memang tipikal evaluasi intervensi citra tubuh karena memperhitungkan durasi kapan efeknya bisa terlihat [5, 14, 54, 78]. Namun, studi replikasi dengan tindak lanjut yang lebih lama akan lebih baik untuk menentukan efek jangka panjang *Warna-Warni Waktu* yang mungkin timbul. Terakhir, meskipun studi ini secara akurat mereproduksi kegiatan intervensi sebagaimana ditampilkan di media sosial untuk tujuan pengumpulan data kepatuhan, studi ini tidak memiliki validitas ekologis karena tidak mengevaluasi *Warna-Warni Waktu* di *platform* media sosial tempatnya akan disebarluaskan (yaitu Facebook, Instagram, dan Youtube). Studi menunjukkan bahwa peningkatan kepatuhan intervensi dan dampak tambahan dari pembelajaran bersama dapat terjadi ketika intervensi disebarluaskan di media sosial (misalnya [79, 80]). Selanjutnya, peran teman dalam situs jejaring sosial telah terbukti meningkatkan diseminasi dan menjadi penguat positif untuk intervensi *eHealth* [30, 81], sehingga mendukung evaluasi *Warna-Warni Waktu* di masa depan di lingkungan target kelompoknya.

Kesimpulan

Uji acak terkontrol paralel ini telah menunjukkan bahwa *Warna-Warni Waktu* adalah intervensi yang efektif dan terukur, yang dapat diterapkan di media sosial untuk meningkatkan kepuasan tubuh pada remaja perempuan Indonesia. Intervensi berbasis media sosial ini merupakan intervensi *eHealth* pertama sejenis. Dengan demikian, penelitian ini dapat dijadikan cetak biru untuk pengembangan intervensi dalam konteks LMIC lainnya di mana kehadiran tatap muka dan perorangan sulit dilakukan tetapi jangkauan internet tinggi. Mengingat kecilnya ukuran efek, *Warna-Warni Waktu* tidak boleh dianggap sebagai terapi untuk remaja perempuan Indonesia yang mengalami masalah terkait citra tubuh yang serius dan mungkin memerlukan perawatan yang lebih intensif. Sebaliknya, intervensi ini sebaiknya dianggap sebagai salah satu alat yang efektif yang dapat digunakan bersama dengan intervensi lain yang ditujukan untuk mengatasi masalah citra tubuh di kalangan remaja perempuan Indonesia.

Terima Kasih

Kami ingin berterima kasih secara khusus kepada Laura Baines (Girl Effect) dan Samantha Jackson (Percolate Galactic) atas kontribusi besar dalam penciptaan *Warna-Warni Waktu*. Kami juga sangat menghargai dan berterima kasih kepada Andika Wijaya yang telah menerjemahkan banyak materi yang digunakan dalam uji coba. Selain itu, kami mengucapkan terima kasih kepada Infinity CXT, lembaga penelitian Indonesia yang melakukan pengumpulan data. Terakhir, kami berterima kasih kepada para remaja perempuan Indonesia yang terlibat dalam penciptaan *Warna-Warni Waktu* untuk kontribusi yang sangat berharga dan mendalam. Studi ini didanai oleh dana penelitian dari Dove Self-Esteem Project (Unilever). Pemberi dana tidak mengambil bagian dalam analisis data, keputusan penerbitan, atau persiapan naskah. Dove Self-Esteem Project (Unilever) diizinkan untuk meninjau manuskrip dan menyarankan perubahan, tetapi para penulis secara eksklusif memiliki keputusan akhir tentang isi manuskrip. Pemikiran yang diungkapkan adalah dari para penulis dan belum tentu dari Unilever.

Konflik Kepentingan

PD dan SH adalah konsultan independen untuk programpendidikan global Dove Self-Esteem Project dan bagian dari dewan penasihat Dove Self-Esteem Project Global pada 2013-2016. PD adalah konsultan independen untuk Instagram yang dimiliki oleh Meta (perusahaan induk Facebook). Intervensi yang dievaluasi di sini dimiliki oleh Girl Effect. Para penulis menyatakan tidak ada konflik kepentingan lain sehubungan dengan proyek ini.

Referensi

Daftar lengkap referensi dapat dilihat di bagian *Reference* versi Bahasa Inggris setelah *Multimedia Appendix*.
