# Supplementary material for: Evaluating the Efficacy of a Social Media–Based Intervention (Warna-Warni Waktu) to Improve Body Image Among Young Indonesian Women: Parallel Randomized Controlled Trial
Source: J Med Internet Res. 2023 Apr 3;25:e42499. doi: 10.2196/42499 (PMC10131926; doi:10.2196/42499)
Supplement: Multimedia Appendix 2 [file jmir_v25i1e42499_app2.docx]

**Multimedia Appendix 2.** Missing items for each trait outcome per condition at each time point.

| Trait outcome measure | | T1^a^ | | T2^b^ | | T3^c^ | |
| --- | --- | --- | --- | --- | --- | --- | --- |
|  |  | Control  (N=923) | Int.^d^  (N=924) | Control  (N=923) | Int.^d^  (N=924) | Control  (N=923) | Int.^d^  (N=924) |
| **Primary outcome measure, N (%)** | |  |  |  |  |  |  |
|  | BESAA^e^ | 2  (0.01) | 4  (0.25) | 526 (3.35) | 895 (5.69) | 506 (3.22) | 523 (3.32) |
| **Secondary outcome measure, N (%)** | |  |  |  |  |  |  |
|  | Internalisation subscale of SATAQ-3^f^ | 2  (0.01) | 12  (0.1) | 373 (3.36) | 648 (5.84) | 360 (3.25) | 373 (3.36) |
|  | Positive affect subscale of PANAS-C^g^ | 64  (0.57) | 108  (0.97) | 420 (3.79) | 701 (6.32) | 371 (3.34) | 387 (3.49) |
|  | Negative affect subscale of PANAS-C^h^ | 49  (0.37) | 97  (0.74) | 495 (3.83) | 613 (4.73) | 422 (3.25) | 446 (3.44) |
|  | Skin shade dissatisfaction^i^ | 8  (0.43) | 10  (0.54) | 71  (3.84) | 116 (6.27) | 64  (3.46) | 65  (3.51) |

^a^Time 1, baseline.

^b^Time 2, 1 day post-intervention.

^c^Time 3, 1 month post-intervention.

^d^Intervention group.

^e^Body Esteem Scale for Adolescents and Adults (21 items).

^f^Sociocultural Attitudes Towards Appearance Questionnaire (12 items).

^g^Positive and Negative Affect Schedule for Children (12 items).

^h^Positive and Negative Affect Schedule for Children (14 items).

^i^2 items.
