# Supplementary material for: Evaluating the Efficacy of a Social Media–Based Intervention (Warna-Warni Waktu) to Improve Body Image Among Young Indonesian Women: Parallel Randomized Controlled Trial
Source: J Med Internet Res. 2023 Apr 3;25:e42499. doi: 10.2196/42499 (PMC10131926; doi:10.2196/42499)
Supplement: Multimedia Appendix 3 [file jmir_v25i1e42499_app3.docx]

**Multimedia Appendix 3.** The number of participants who completed at least 80% of items on each trait measure.

|  | | Control (N=923) | | | Intervention (N=924) | | |
| --- | --- | --- | --- | --- | --- | --- | --- |
| Measure | | T1^a^ | T2 | T3 | T1^a^ | T2 | T3 |
| **Primary outcome measure, n (%)** | |  | | | | | |
|  | BESAA^b^ | 923 (100) | 892 (96.6) | 891 (96.5) | 924 (100) | 871 (94.3) | 893 (96.64) |
| **Secondary outcome measure, n (%)** | |  | | | | | |
|  | Internalisation subscale of SATAQ^c^-3 | 923 (100) | 892 (96.6) | 893 (96.7) | 922 (99.8) | 870 (94.2) | 893 (96.64) |
|  | Positive affect subscale of PANAS-C^d^ | 916 (99.2) | 887 (96.1) | 891 (96.5) | 909 (98.3) | 865 (93.6) | 890 (96.3) |
|  | Negative affect subscale of PANAS-C^d^ | 920 (99.7) | 888 (96.2) | 893 (96.7) | 915 (99.0) | 866 (93.7) | 892 (96.5) |
|  | Skin shade satisfaction | 919 (99.6) | 887 (96.1) | 890 (96.4) | 918 (99.3) | 865 (93.6) | 890 (96.3) |

^a^Participants included in the intention-to-treat analysis.

^b^Body Esteem Scale for Adolescents and Adults.

^c^Sociocultural Attitudes Towards Appearance Questionnaire.

^d^Positive and Negative Affect Schedule for Children.
