# Supplementary material for: Evaluating the Efficacy of a Social Media–Based Intervention (Warna-Warni Waktu) to Improve Body Image Among Young Indonesian Women: Parallel Randomized Controlled Trial
Source: J Med Internet Res. 2023 Apr 3;25:e42499. doi: 10.2196/42499 (PMC10131926; doi:10.2196/42499)
Supplement: Multimedia Appendix 4 [file jmir_v25i1e42499_app4.docx]

**Multimedia Appendix 4.** Retention rates for state outcomes in the intervention group (N = 924).

|  | Body Satisfaction | | Mood | |
| --- | --- | --- | --- | --- |
|  | N | Retention rate (%) | N | Retention rate (%) |
| Pre-video 1 | 812 | 87.87 | 813 | 89.14 |
| Post-video 1 | 812 | 87.87 | 813 | 89.14 |
| Pre-video 2 | 802 | 86.79 | 802 | 86.79 |
| Post-video 2 | 802 | 86.79 | 802 | 86.79 |
| Pre-video 3 | 800 | 86.58 | 799 | 86.47 |
| Post-video 3 | 800 | 86.58 | 799 | 86.47 |
| Pre-video 4 | 799 | 86.47 | 799 | 86.47 |
| Post-video 4 | 799 | 86.47 | 799 | 86.47 |
| Pre-video 5 | 797 | 86.25 | 797 | 86.25 |
| Post-video 5 | 797 | 86.25 | 797 | 86.25 |
| Pre-video 6 | 796 | 86.15 | 795 | 86.03 |
| Post-video 6 | 796 | 86.15 | 795 | 86.03 |
