# Supplementary material for: Evaluating the Efficacy of a Social Media–Based Intervention (Warna-Warni Waktu) to Improve Body Image Among Young Indonesian Women: Parallel Randomized Controlled Trial
Source: J Med Internet Res. 2023 Apr 3;25:e42499. doi: 10.2196/42499 (PMC10131926; doi:10.2196/42499)
Supplement: Multimedia Appendix 5 [file jmir_v25i1e42499_app5.docx]

**Multimedia Appendix 5.** Complete participant ethnicity data.

|  | | Total  (N = 1847) | Control  (N = 923) | Intervention  (N = 924) |
| --- | --- | --- | --- | --- |
| **Ethnicity, n (%)** | |  |  |  |
|  | Acehnese | 5 (0.3) | 4 (0.4) | 1 (0.1) |
|  | Ambonese | 2 (0.1) | 1 (0.1) | 1 (0.1) |
|  | Ambonese, Belanda | 1 (0.05) | -- | 1 (0.1) |
|  | Ambonese, Minahasan | 1 (0.05) | -- | 1 (0.1) |
|  | Ambonese, Javanese | 1 (0.05) | 1 (0.1) | -- |
|  | Arabic, Buginese | 1 (0.05) | 1 (0.1) | -- |
|  | Banjarese | 10 (0.5) | 7 (0.8) | 3 (0.3) |
|  | Banjarese, Javanese | 2 (0.1) | 2 (0.2) | -- |
|  | Banjarese, Kutai | 1 (0.05) | -- | 1 (0.1) |
|  | Bantanese | 1 (0.05) | -- | 1 (0.1) |
|  | Bataknese | 74 (4.0) | 28 (3.0) | 46 (5.0) |
|  | Betawi | 84 (4.6) | 46 (5.0) | 38 (4.1) |
|  | Betawi, Javanese | 13 (0.7) | 12 (1.3) | 1 (0.1) |
|  | Betawi, Minangnese | 2 (0.1) | -- | 2 (0.2) |
|  | Betawi, Sundanese | 8 (0.4) | 2 (0.2) | 6 (0.7) |
|  | Betawi, Sundanese, Javanese | 1 (0.05) | 1 (0.1) | -- |
|  | Bimanese | 1 (0.05) | 1 (0.1) | -- |
|  | Bolaang Mongondow | 3 (0.2) | 2 (0.2) | 1 (0.1) |
|  | Buginese | 39 (2.1) | 19 (2.1) | 20 (2.2) |
|  | Buginese, Javanese | 4 (0.2) | 2 (0.2) | 2 (0.2) |
|  | Buginese, Makassarese | 4 (0.2) | 2 (0.2) | 2 (0.2) |
|  | Buton | 3 (0.2) | 1 (0.1) | 2 (0.2) |
|  | Buton, Javanese | 1 (0.05) | -- | 1 (0.1) |
|  | Chinese | 2 (0.1) | 1 (0.1) | 1 (0.1) |
|  | Chinese, Arabic | 1 (0.05) | -- | 1 (0.1) |
|  | Chinese, Batak | 1 (0.05) | -- | 1 (0.1) |
|  | Chinese, Malay | 1 (0.05) | -- | 1 (0.1) |
|  | Chinese, Sundanese | 1 (0.05) | -- | 1 (0.1) |
|  | Dayak | 1 (0.05) | -- | 1 (0.1) |
|  | Gorontalo | 2 (0.1) | 1 (0.1) | 1 (0.1) |
|  | Javanese | 665 (36.0) | 344 (37.3) | 321 (34.7) |
|  | Javanese, Acehnese | 1 (0.05) | -- | 1 (0.1) |
|  | Javanese, Dayak | 2 (0.1) | 1 (0.1) | 1 (0.1) |
|  | Javanese, Lampungese | 1 (0.05) | 1 (0.1) | -- |
|  | Javanese, Madura | 2 (0.1) | 1 (0.1) | 1 (0.1) |
|  | Javanese, Malay | 3 (0.2) | 2 (0.2) | 1 (0.1) |
|  | Javanese, Minangnese | 3 (0.2) | 1 (0.1) | 2 (0.2) |
|  | Javanese, Palembangese | 3 (0.2) | 1 (0.1) | 2 (0.2) |
|  | Javanese, Sangirese | 1 (0.05) | 1 (0.1) | -- |
|  | Javanese, Sundanese | 14 (0.8) | 4 (0.4) | 10 (1.1) |
|  | Javanese, Torajan | 1 (0.05) | 1 (0.1) | -- |
|  | Javanese, Banjarese, Buginese | 1 (0.05) | -- | 1 (0.1) |
|  | Javanese, Malay, Buginese | 1 (0.05) | -- | 1 (0.1) |
|  | Javanese, Sundanese, Buginese | 1 (0.05) | -- | 1 (0.1) |
|  | Karonese | 2 (0.1) | 2 (0.2) | -- |
|  | Komering | 2 (0.1) | 2 (0.2) | -- |
|  | Lampungese | 1 (0.05) | -- | 1 (0.1) |
|  | Madurese | 6 (0.3) | 1 (0.1) | 5 (0.5) |
|  | Makassarese | 47 (2.5) | 23 (2.5) | 24 (2.6) |
|  | Malay | 53 (2.9) | 26 (2.8) | 27 (2.9) |
|  | Malay, Arabic | 1 (0.05) | -- | 1 (0.1) |
|  | Malay, Minangnese | 1 (0.05) | -- | 1 (0.1) |
|  | Malay, Palembangese | 1 (0.05) | 1 (0.1) | -- |
|  | Malay, Karonese, Minangnese | 1 (0.05) | 1 (0.1) | -- |
|  | Mandailing | 7 (0.4) | 3 (0.3) | 4 (0.4) |
|  | Manderese | 1 (0.05) | 1 (0.1) | -- |
|  | Minahasan | 9 (0.5) | 5 (0.5) | 4 (0.4) |
|  | Minahasan, Sangirese | 1 (0.05) | 1 (0.1) | -- |
|  | Minangnese | 34 (1.8) | 17 (1.8) | 17 (1.8) |
|  | Minangnese, Acehnese | 1 (0.05) | 1 (0.1) | -- |
|  | Minangnese, Bataknese | 1 (0.05) | 1 (0.1) | -- |
|  | Minangnese, Bugisnese | 1 (0.05) | -- | 1 (0.1) |
|  | Nias | 1 (0.05) | 1 (0.1) | -- |
|  | Nias, Javanese | 1 (0.05) | -- | 1 (0.1) |
|  | Palembangese | 68 (3.7) | 30 (3.3) | 38 (4.1) |
|  | Sangirese | 1 (0.05) | -- | 1 (0.1) |
|  | Semendo | 1 (0.05) | 1 (0.1) | -- |
|  | Sundanese | 255 (13.8) | 123 (13.3) | 132 (14.3) |
|  | Sundanese, Banjarese | 1 (0.05) | -- | 1 (0.1) |
|  | Sundanese, Buginese | 1 (0.05) | 1 (0.1) | -- |
|  | Sundanese, Minangnese | 3 (0.2) | 2 (0.2) | 1 (0.1) |
|  | Sundanese, Buginese, Palembangese | 1 (0.05) | -- | 1 (0.1) |
|  | Sundanese, Javanese, Bataknese | 1 (0.05) | -- | 1 (0.1) |
|  | Torajan | 2 (0.1) | 1 (0.1) | 1 (0.1) |
|  | Didn’t know | 140 (7.6) | 79 (8.6) | 61 (6.6) |
|  | Incorrect response (eg, city of residence) | 148 (8.0) | 65 (7.0) | 83 (9.0) |
|  | Didn’t respond | 83 (4.5) | 44 (4.8) | 39 (4.3) |
