# Supplementary material for: Evaluating the Efficacy of a Social Media–Based Intervention (Warna-Warni Waktu) to Improve Body Image Among Young Indonesian Women: Parallel Randomized Controlled Trial
Source: J Med Internet Res. 2023 Apr 3;25:e42499. doi: 10.2196/42499 (PMC10131926; doi:10.2196/42499)
Supplement: Multimedia Appendix 6 [file jmir_v25i1e42499_app6.docx]

## **Multimedia Appendix 6**. Number of minutes to complete each time point survey.

|  | Control  (N = 923) | Intervention  (N = 924) | *t* test (*df*)^b^ | *P* value |
| --- | --- | --- | --- | --- |
| T1^a^ survey, mean (SD) | 30.17 (71.02) | 33.75 (83.66) | -0.99 (1845) | .32 |
| T2^c^ survey, mean (SD) | 26.95 (87.07) | 38.15 (122.03) | -2.22 (1761) | .026 |
| T3^d^ survey, mean (SD) | 22.14 (77.20) | 19.23 (66.19) | 0.87 (1845) | .38 |

^a^Time 1, baseline.

^b^2-tailed.

^c^Time 2, 1 day post-intervention.

^d^Time 3, 1 month post-intervention.
