# Supplementary material for: Evaluating the Efficacy of a Social Media–Based Intervention (Warna-Warni Waktu) to Improve Body Image Among Young Indonesian Women: Parallel Randomized Controlled Trial
Source: J Med Internet Res. 2023 Apr 3;25:e42499. doi: 10.2196/42499 (PMC10131926; doi:10.2196/42499)
Supplement: Multimedia Appendix 7 [file jmir_v25i1e42499_app7.docx]

**Multimedia Appendix 7.** Intervention adherence: Videos viewed and activities completed.

|  | | |
| --- | --- | --- |
| Video | Intervention participants^a^ | Intervention participants (%) |
| 1 | 814 | 88.09 |
| 2 | 803 | 86.90 |
| 3 | 801 | 86.68 |
| 4 | 803 | 86.90 |
| 5 | 797 | 86.25 |
| 6 | 797 | 86.25 |
|  |  |  |
| Activity |  |  |
| 1 | 735 | 79.54 |
| 2 | 748 | 80.95 |
| 3 | 729 | 78.89 |
| 4 | 754 | 81.60 |
| 5 | 736 | 79.65 |
| 6 | 715 | 77.38 |
| 7 | 755 | 81.70 |
| 8 | 754 | 81.60 |
| 9 | 737 | 79.76 |
| 10 | 754 | 81.60 |
| 11 | 766 | 82.90 |
| 12 | 728 | 78.78 |
| 13 | 737 | 79.76 |
| 14 | 741 | 80.19 |
| 15 | 731 | 79.11 |
| 16 | 730 | 79.0 |
| 17 | 719 | 77.81 |
| 18 | 789 | 85.38 |

^a^N=924

|  | | |
| --- | --- | --- |
| Video |  |  |
| 1 |  |  |
| 2 |  |  |
| 3 |  |  |
| 4 |  |  |
| 5 |  |  |
| 6 |  |  |
|  | | |
| Activity |  |  |
|  |  |  |
|  |  |  |
|  |  |  |
|  |  |  |
|  |  |  |
|  |  |  |
|  |  |  |
|  |  |  |
|  |  |  |
|  |  |  |
|  |  |  |
|  |  |  |
|  |  |  |
|  |  |  |
|  |  |  |
