# Supplementary material for: Evaluating the Efficacy of a Social Media–Based Intervention (Warna-Warni Waktu) to Improve Body Image Among Young Indonesian Women: Parallel Randomized Controlled Trial
Source: J Med Internet Res. 2023 Apr 3;25:e42499. doi: 10.2196/42499 (PMC10131926; doi:10.2196/42499)
Supplement: Multimedia Appendix 8 [file jmir_v25i1e42499_app8.docx]

**Multimedia Appendix 8**. Trait outcomes as per linear mixed models (LMMs) executed with 21 dimensions and an unstructured covariance structure.

| **Trait Body Satisfaction** | | | | |
| --- | --- | --- | --- | --- |
| −2 restricted log likelihood | | 2778.823 | | |
|  | | | | |
|  | | *F* test (*df*) | P value (REML)^a^ | Partial η2 |
| Type III test of fixed effects | | | | |
|  | Group | 1.28 (1,1803.89) | .26 | 0.0007 |
|  | Time (T2, T3) | 46.47 (1,1743.05) | <.001 | 0.0259 |
|  | Group×Time | 1.26 (1,1743.05) | .26 | 0.0007 |
|  | Baseline (covariate) | 2776.98 (1,1804.98) | <.0001 | 0.6060 |
|  | Group×Covariate | 0.81 (1,1804.98) | .37 | 0.0004 |
|  | Time×Covariate | 43.07 (1,1744.18) | <.001 | 0.0241 |
|  | Group×Time×Covariate | 0.15 (1,1744.18) | .70 | 0.0001 |
|  | | Chi-square (*df*) | Critical chi-square | *P* value |
| −2 log likelihood test for effect of group | | 19.45‬ (4) | 9.49 | <.001 |
| **Trait internalization** | | | | |
| −2 restricted log likelihood | | 5574.82 | | |
|  | | | | |
|  | | *F* test (*df*) | *P* value (REML) | Partial η2 |
| Type III test of fixed effects | | | | |
|  | Group | 2.00 (1,1808.10) | .16 | 0.0011 |
|  | Time (T2, T3) | 15.55 (1,1748.60) | <.001 | 0.0088 |
|  | Group×Time | 4.92 (1,1748.60) | .03 | 0.0028 |
|  | Baseline (covariate) | 1816.57 (1,1805.26) | <.001 | 0.5015 |
|  | Group×Covariate | 0.64 (1,1805.26) | .42 | 0.0003 |
|  | Time×Covariate | 15.21 (1,1746.10) | <.001 | 0.0086 |
|  | Group×Time×Covariate | 6.62 (1,1746.10) | .01 | 0.0037 |
|  | | Chi-square (*df*) | Critical chi-square | *P* value |
| −2 log likelihood test for effect of group | | 45.06 (4) | 9.49 | <.001 |
| **Trait skin dissatisfaction** | | | | |
| −2 restricted log likelihood | | 8789.92 | | |
|  | | | | |
|  | | *F* test (*df*) | *P* value (REML) | Partial η2 |
| Type III test of fixed effects | | | | |
|  | Group | 1.33 (1, 1810.38) | .25 | 0.0007 |
|  | Time (T2, T3) | 3.17 (1, 1764.73) | .07 | 0.0017 |
|  | Group×Time | 0.19 (1, 1764.73) | .66 | 0.0001 |
|  | Baseline (covariate) | 1007.41 (1, 1812.04) | <.001 | 0.3573 |
|  | Group×Covariate | 0.60 (1, 1812.04) | .44 | 0.0003 |
|  | Time×Covariate | 6.37 (1, 1766.30) | .01 | 0.0035 |
|  | Group×Time×Covariate | 2.25 (1, 1766.30) | .13 | 0.0012 |
|  | | Chi-square (*df*) | Critical chi-square | *P* value |
| −2 log likelihood test for effect of group | | 11.2 (4)‬ | 9.49 | .03 |
| **Trait positive mood** | | | | |
| −2 restricted log likelihood | | 4490.78 | | |
|  | | | | |
|  | | *F* test (*df*) | *P* value (REML) | Partial η2 |
| Type III test of fixed effects | | | | |
|  | Group | 0.958 (1,1785.34) | .33 | 0.0005 |
|  | Time (T2, T3) | 11.56 (1,1729.25) | <.001 | 0.0066 |
|  | Group×Time | 1.362 (1,1729.25) | .24 | 0.0007 |
|  | Baseline (covariate) | 1917.33 (1,1788.42) | <.001 | 0.5173 |
|  | Group×Covariate | 0.93 (1,1788.42) | .33 | 0.0005 |
|  | Time×Covariate | 10.77 (1,1732.60) | .001 | 0.0061 |
|  | Group×Time×Covariate | 0.77 (1,1732.60) | .38 | 0.0004 |
|  | | | | |
|  | | Chi-square (*df*) | Critical chi-square | *P* value |
| −2 log likelihood test for effect of group | | 4.48 (4) | 9.49 | .41 |
| **Trait negative mood** | | | | |
| −2 restricted log likelihood | | 4574.02 | | |
|  | | | | |
|  | | *F* test (*df*) | *P* value (REML) | Partial η2 |
| Type III test of fixed effects | | | | |
|  | Group | 0 (1,1812.85) | .97 | 0.0000 |
|  | Time (T2, T3) | 0.67 (1,1759.46) | .41 | 0.0004 |
|  | Group×Time | 0.44 (1,1759.46) | .51 | 0.0002 |
|  | Baseline (covariate) | 1773.33 (1,1812.47) | <.001 | 0.4945 |
|  | Group×Covariate | 0 (1,1812.47) | .99 | 0.0000 |
|  | Time×Covariate | 0.041 (1,1759.19) | .84 | 0.00002 |
|  | Group×Time×Covariate | 1.60 (1,1759.19) | .20 | 0.0009 |
|  | | | | |
|  | | Chi-square (*df*) | Critical chi-square | *P* value |
| −2 log likelihood test for effect of group | | 9.28 (4) | 9.49 | .06 |

^a^Restricted maximum likelihood.
