# Supplementary material for: Evaluating the Efficacy of a Social Media–Based Intervention (Warna-Warni Waktu) to Improve Body Image Among Young Indonesian Women: Parallel Randomized Controlled Trial
Source: J Med Internet Res. 2023 Apr 3;25:e42499. doi: 10.2196/42499 (PMC10131926; doi:10.2196/42499)
Supplement: Multimedia Appendix 9 [file jmir_v25i1e42499_app9.docx]

**Multimedia Appendix 9.** Cumulative analyses for state outcomes.

| State Body Satisfaction | | | *F* test (*df*) | | *P* value |
| --- | --- | --- | --- | --- | --- |
| **2×6 ANOVA (Within-subjects effects: Greenhouse-Geisser)** | | | | | |
|  | Pre-Post | | 502.6 (1,662) | | <.001 |
|  | Videos | | 150.08 (1,662) | | <.001 |
|  | Pre-Post×Videos | | 4.86 (1,662) | | <.001 |
| **Trends for interaction effect** | | | | | |
|  | Linear | | 1.48 (1,662) | | .22 |
|  | Quadratic | | 8.85 (1,662) | | .003 |
|  | Cubic | | 0.20 (1,662) | | .65 |
| Adjacent points *t* tests for gain scores | Values, mean (SD) | Gain score comparisons |  | *t* test (*df*) | *P* value^a^ |
| Gain score for video 1 | 6.93 (15.5) | Video 1 vs video 2 | 764 | 2.86 | .004 |
| Gain score for video 2 | 4.94 (10.5) | Video 2 vs video 3 | 763 | −1.96 | .050 |
| Gain score for video 3 | 5.75 (10.6) | Video 3 vs video 4 | 766 | 4.17 | <.001 |
| Gain score for video 4 | 4.01 (8.8) | Video 4 vs video 5 | 761 | −2.74 | .006 |
| Gain score for video 5 | 5.22 (9.8) | Video 5 vs video 6 | 760 | −0.78 | .432 |
| Gain score for video 6 | 5.68 (10.4) |  |  | | |
| RM ANOVA prevideo scores (within-subjects effects: Sphericity assumed) | | | *F* test (*df*) | | *P* value |
| Videos | | | 120.30 (5,3685) | | <.001 |
| **Repeated contrasts** | | | | | |
|  | Video 1 vs video 2 | | 71.93 (1,737) | | <.001 |
|  | Video 2 vs video 3 | | 52.05 (1,737) | | <.001 |
|  | Video 3 vs video 4 | | 12.03 (1,737) | | <.001 |
|  | Video 4 vs video 5 | | 3.20 (1,737) | | .057 |
|  | Video 5 vs video 6 | | 10.10 (1,737) | | .002 |
| RM ANOVA postvideo scores (within-subjects effects: Sphericity assumed) | | | *F* test (*df*) | | *P* value |
| Videos | | | 118.43 (5,3325) | | <.001 |
| **Repeated contrasts** | | | | | |
|  | Video 1 vs video 2 | | 46.62 (1,665) | | <.001 |
|  | Video 2 vs video 3 | | 73.10 (1,665) | | <.001 |
|  | Video 3 vs video 4 | | 0.46 (1,665) | | .50 |
|  | Video 4 vs video 5 | | 26.10 (1,665) | | <.001 |
|  | Video 5 vs video 6 | | 20.61 (1,665) | | <.001 |
| State Mood | | | *F* test (*df*) | | *P* value |
| **2×6 ANOVA (within-subjects effects: Greenhouse-Geisser)** | | | | | |
|  | Pre-post | | 408.13 (1,664) | | <.001 |
|  | Videos | | 48.14 (1,664) | | <.001 |
|  | Pre-post×videos | | 11.16 (1,664) | | <.001 |
| **Trends for interaction effect** | | | | | |
|  | Linear | | 3.77 (1,664) | | .053 |
|  | Quadratic | | 30.22 (1,664) | | <.001 |
|  | Cubic | | 5.86 (1,664) | | .016 |
| Adjacent points *t* tests for gain scores | Values, mean (SD) | Gain score comparisons | *t* test (*df*) | | *P* value^a^ |
| Gain score for video 1 | 4.31 (12.2) | Video 1 vs video 2 | 1.25 (765) | | .21 |
| Gain score for video 2 | 3.75 (10.6) | Video 2 vs video 3 | −1.34 (763) | | .18 |
| Gain score for video 3 | 4.35 (9.6) | Video 3 vs video 4 | 0.16 (766) | | .87 |
| Gain score for video 4 | 4.07 (9.3) | Video 4 vs video 5 | −0.93 (761) | | .35 |
| Gain score for video 5 | 4.47 (9.5) | Video 5 vs video 6 | −2.70 (760) | | .007 |
| Gain score for video 6 | 5.75 (11.7) | —^b^ | —^b^ | | —^b^ |
| RM ANOVA prevideo scores (within-subjects effects: Sphericity assumed) | | | *F* test (*df*) | | *P* value |
| Videos | | | 46.67 (5,3690) | | <.001 |
| **Repeated contrasts** | | | | | |
|  | Video 1 vs video 2 | | 37.60 (1,738) | | <.001 |
|  | Video 2 vs video 3 | | 17.02 (1,738) | | .36 |
|  | Video 3 vs video 4 | | 0.84 ( 1,738) | | .38 |
|  | Video 4 vs video 5 | | 0.78 (1,738) | | .38 |
|  | Video 5 vs video 6 | | 0.76 (1,738) | | —^b^ |
| RM ANOVA postvideo scores (within-subjects effects: Sphericity assumed) | | | *F* test (*df*) | | *P* value |
| Videos | | | 33.81 (5,3330) | | <.001 |
| **Repeated contrasts** | | | | | |
|  | Video 1 vs video 2 | | 4.80 (1,666) | | .029 |
|  | Video 2 vs video 3 | | 24.52 (1,666) | | <.001 |
|  | Video 3 vs video 4 | | 0.23 (1,666) | | .63 |
|  | Video 4 vs video 5 | | 2.402 (1,666) | | .12 |
|  | Video 5 vs video 6 | | 12.90 (1,666) | | <.001 |

^a^2-sided.

^b^Not applicable.
