# Supplementary material for: Evaluating the Efficacy of a Social Media–Based Intervention (Warna-Warni Waktu) to Improve Body Image Among Young Indonesian Women: Parallel Randomized Controlled Trial
Source: J Med Internet Res. 2023 Apr 3;25:e42499. doi: 10.2196/42499 (PMC10131926; doi:10.2196/42499)
Supplement: Multimedia Appendix 10 [file jmir_v25i1e42499_app10.docx]

**Multimedia Appendix 10.** Exploratory post-hoc analyses testing the differential effect of the intervention on participants with higher vs lower levels of body satisfaction at baseline.

| Differential scores | Low body satisfaction at baseline  n = 348 | High body satisfaction at baseline  n = 323 | Independent sample t-tests |
| --- | --- | --- | --- |
| T1-T2 | | | |
| Delta body satisfaction | 0.12 (0.40) | −0.15 (0.40) | t (669) = 8.76, p(two-sided)<.001,  95% C.I.: .21, 0.33 |
| Delta internalization | −0.26 (0.65) | −0.10 (0.72) | t (669) = −3.05, p(two-sided)<.01,  95% C.I.: −.26, −.06 |
| Delta skin dissatisfaction | −0.18 (0.97) | −0.16 (1.06) | t (663) = −.19, p(two-sided)=.848,  95% C.I.: −.17, .14 |
| T1-T3 | | | |
| Delta body satisfaction | 0.21 (0.46) | −0.15 (0.44) | t (682) = 10.44, p(two-sided)<.001,  95% C.I.: .29, .43 |
| Delta internalization | −0.32 (0.71) | -0.07 (0.71) | t (682) = −4.57, p(two-sided)<.001,  95% C.I.: -.35, −.14 |
| Delta skin dissatisfaction | −0.15 (1.06) | −0.19 (1.06) | t (679) = .466, p(two-sided)=.64,  95% C.I.: −.12, .19 |

- In the intervention group only, we created a binary variable indicating either low and high Body satisfaction at T1. Participants with a T1 body satisfaction score falling within the first 40% of the T1 body satisfaction distribution were assigned to the ‘Low body satisfaction at baseline’ group. Participants falling in the last 40% of the distribution were assigned to the ‘High body satisfaction at baseline’ group. The central 20% of the T1 body satisfaction distribution was excluded from the analyses. Note there are no standardized cut-offs for high and low body satisfaction for the Body Esteem Scale for Adolescents and Adults.
- We calculated differential outcome variables by subtracting T1 scores from either T2 or T3 scores for Body satisfaction, Internalization, and Skin dissatisfaction.
- Next, we compared the two aforementioned groups on the differential scores running independent sample t-tests as presented in the table above.
- The results illustrated that lower levels of body satisfaction at baseline presented significantly larger improvement in body satisfaction from T1 and T2 as well as from T1 and T3 when compared to participants with higher levels of body satisfaction at baseline. Similarly, participants with lower levels of body satisfaction at baseline showed larger drops in both internalization both between T1 and T2 as well as from T1 to T3 when compared to participants with higher levels of body satisfaction at baseline. There were no significant differences between groups for differential scores of skin dissatisfaction.
